# Design, Synthesis and Bioactivity Evaluation of Novel 2-(pyrazol-4-yl)-1,3,4-oxadiazoles Containing an Imidazole Fragment as Antibacterial Agents

**DOI:** 10.3390/molecules28062442

**Published:** 2023-03-07

**Authors:** Hongwu Liu, Shan Yang, Ting Li, Siyue Ma, Peiyi Wang, Guoqing Wang, Shanshan Su, Yue Ding, Linli Yang, Xiang Zhou, Song Yang

**Affiliations:** National Key Laboratory of Green Pesticide, Key Laboratory of Green Pesticide and Agricultural Bioengineering, Ministry of Education, Center for R&D of Fine Chemicals of Guizhou University, Guiyang 550025, China

**Keywords:** imidazole derivatives, antibacterial activity, plant bacterial disease, antibacterial mechanism, cell membrane

## Abstract

Imidazole alkaloids, a common class of five-membered aromatic heterocyclic compounds, exist widely in plants, animals and marine organisms. Because of imidazole’s extensive and excellent biological and pharmacological activities, it has always been a topic of major interest for researchers and has been widely used as an active moiety in search of bioactive molecules. To find more efficient antibacterial compounds, a series of novel imidazole-fragment-decorated 2-(pyrazol-4-yl)-1,3,4-oxadiazoles were designed and synthesized based on our previous works via the active substructure splicing principle, and their bioactivities were systematically evaluated both in vitro and in vivo. The bioassays showed that some of the target compounds displayed excellent in vitro antibacterial activity toward three virulent phytopathogenic bacteria, including *Xanthomonas oryzae* pv. *oryzae* (*Xoo*), *Xanthomonas axonopodis* pv. *citri* (*Xac*) and *Pseudomonas syringae* pv. *actinidiae* (*Psa*), affording the lowest EC_50_ values of 7.40 (**7c**), 5.44 (**9a**) and 12.85 (**9a**) μg/mL, respectively. Meanwhile, compound **7c** possessed good in vivo protective and curative activities to manage rice bacterial leaf blight at 200 μg/mL, with control efficacies of 47.34% and 41.18%, respectively. Furthermore, compound **9a** showed commendable in vivo protective and curative activities to manage kiwifruit bacterial canker at 200 μg/mL, with control efficacies of 46.05% and 32.89%, respectively, which were much better than those of the commercial bactericide **TC** (31.58% and 17.11%, respectively). In addition, the antibacterial mechanism suggested that these new types of title compounds could negatively impact the cell membranes of phytopathogenic bacteria cells and cause the leakage of the intracellular component, thereby leading to the killing of bacteria. All these findings confirm that novel 2-(pyrazol-4-yl)-1,3,4-oxadiazoles containing an imidazole fragment are promising lead compounds for discovering new bactericidal agents.

## 1. Introduction

**Figure 1:**
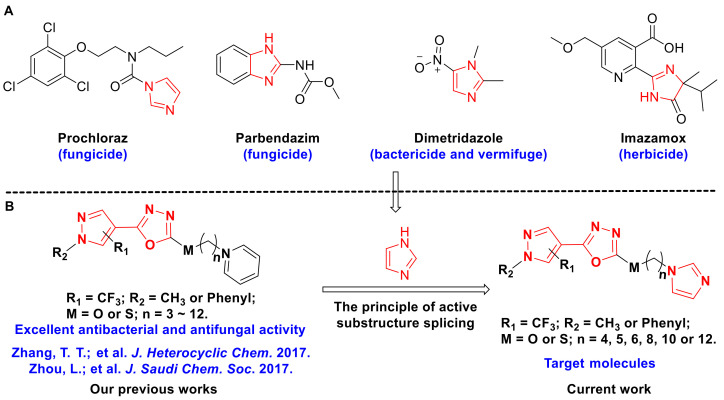
(**A**) Some important commercial agents containing imidazole moieties. (**B**) The design strategy for target molecules [37,38].

Imidazole alkaloids, a common class of five-membered aromatic heterocyclic compounds containing two intersite nitrogen atoms, exist widely in plants, animals, microorganisms and marine organisms [1]. Imidazole is a dominant skeleton in drug development that exists in the core structure of many medicines and pesticides and has been extensively explored by scientific researchers [2,3]. As a good pharmacodynamic group, imidazole can synergically enhance drug efficacy, improve in vitro and in vivo bioactivity, and reduce biotoxicity, and it also possesses excellent bioavailability, good tissue penetration and relatively low adverse reactions [1,2,3]. To date, imidazoles have become one of the most important skeletons in medicine and pesticide discovery, and many novel bioactive molecules have been reported based on this advantageous framework, such as antibacterial [4,5], antifungal [6], antiviral [7], anti-inflammatory [8], anticancer [9], antioxidant [10] and anti-Alzheimer’s disease [11]. Encouragingly, a number of imidazole core-containing medicines and pesticides have been successfully developed, especially in agricultural fields, and imidazole derivatives are widely applied as fungicides, herbicides and insecticides (Figure 1). For example, prochloraz, carbendazim, imazalil, thiabendazole, triflumizole, cyazofamid and pefurazoate are used to control plant fungal diseases [12,13,14,15,16,17], and some of them are also used as fungicides in coatings, synthetic resins, paper products, metal products and household appliances [18]. Meanwhile, dimetridazole and parbendazole are veterinary vermifuges and growth-promoting agents in the breeding industry, as well as feed additives for pigs and chickens [19,20,21]. Moreover, imazamox, imazameth and imazapyr are frequently used herbicide varieties in weeding controls [22,23]. All these applications suggest that imidazole is a good pharmacophore for developing novel bioactive molecules.

Meanwhile, 2-(pyrazol-4-yl)-1,3,4-oxadiazole moiety, is found in many functional molecules and displays broad bioactivities, such as antibacterial [24,25,26,27,28], antifungal [24,25,26,27], antiobesity [29], anticancer [30], antituberculosis [26], antimalarial [26], anti-inflammatory [31], antiviral [32] and DNA photocleaving [33]. The synthesis of this heterocyclic framework has attracted increasing attention in recent years. In our previous studies, some series of novel 2-(pyrazol-4-yl)-1,3,4-oxadiazole derivatives were proven to have excellent antimicrobial activity against devastating phytopathogenic bacteria and fungi [34,35], suggesting that the 2-(pyrazol-4-yl)-1,3,4-oxadiazole unit is the core pharmacophore in developing new agrochemicals. To extend the application potential of the 2-(pyrazol-4-yl)-1,3,4-oxadiazole pharmacophore in the field of agrochemical development, and probe the underlying action mechanism, new types of 2-(pyrazol-4-yl)-1,3,4-oxadiazole derivatives containing an imidazole moiety were prepared, and their application potential to stimulate new agrochemical discovery assessed.

Encouraged by the above investigations and to continue our research on the exploration of more effective antibacterial agents, a series of novel imidazole-tailed 2-(pyrazol-4-yl)-1,3,4-oxadiazoles were designed (Figure 1) by the widely adopted principle—active substructure splicing [34,35,36]. Furthermore, the in vitro and in vivo bioassays of the target compounds were evaluated against three devastating phytopathogenic bacteria via the turbidimetric test and pot experiment, respectively. Meanwhile, the antibacterial mechanism was studied using the mutually supportive assays of scanning electron microscopy (SEM) and propidium iodide (PI) fluorescent staining.

## 2. Results and Discussion

### 2.1. Chemistry

**Scheme 1:**
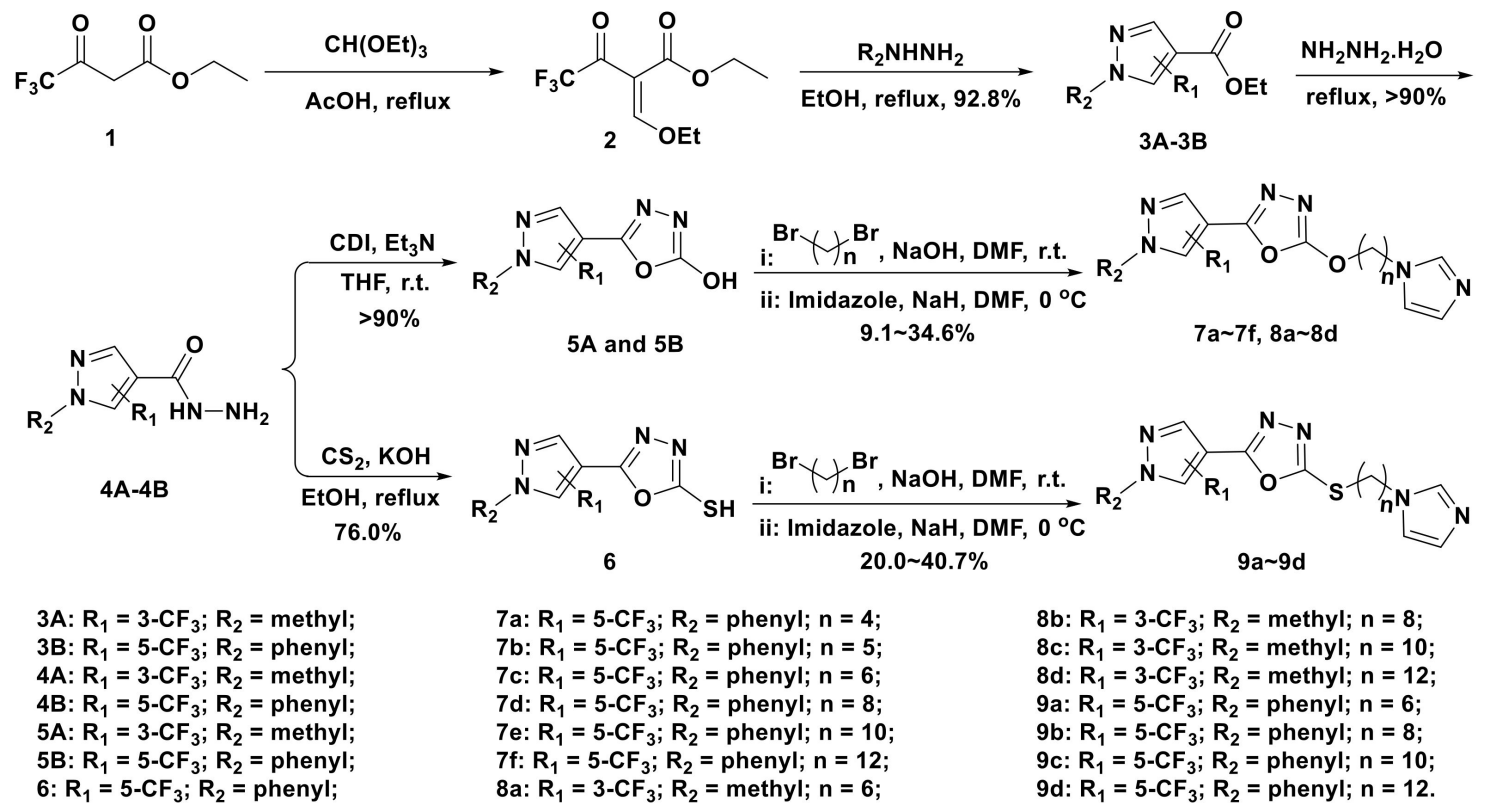
The synthetic route for target compounds **7a**~**7f**, **8a**~**8d** and **9a**~**9d**.

The general organic synthesis of novel 2-(pyrazol-4-yl)-1,3,4-oxadiazoles containing an imidazole moiety is illustrated in Scheme 1. According to our previously described protocols [37,38], the key intermediates 5-(1-phenyl-5-(trifluoromethyl)-1*H*-pyrazol-4-yl)-1,3,4-oxadiazol-2-ol (**5A**), 5-(1-methyl-3-(trifluoromethyl)-1*H*-pyrazol-4-yl)-1,3,4-oxadiazol-2-ol (**5B**) and 5-(1-phenyl-5-(trifluoromethyl)-1*H*-pyrazol-4-yl)-1,3,4-oxadiazole-2-thiol (**6**) were successfully prepared using ethyl 4,4,4-trifluoroacetoacetate (**1**) as the starting material via four steps, including aldol condensation, ring-closing, hydrazinolysis and second ring-closing reactions, respectively. Finally, target compounds **7a**–**7f** were generated after intermediate **5B** underwent continuous nucleophilic substitution with the relevant dibromoalkane and imidazole under a strong alkali atmosphere. Similarly, target compounds **8a**–**8d** and **9a**–**9d** were successfully obtained from intermediates **5A** and **6**, respectively, via the same operations as target compounds **7a–7f**.

Furthermore, all the structures of the title compounds were identified by NMR and HRMS. In the ^1^H NMR spectra of the synthesized title compounds, three single peaks (s) stably occurred in the range of *δ* 7.51–7.40, 7.06–6.98 and 6.91–6.88 ppm, which belonged to protons of the imidazole ring. Other peaks in the range of *δ* 6.90–8.20 ppm were contributed by the pyrazol group and phenyl group, respectively. The triplet peak (t) between 4.00 ppm and 3.90 ppm was the CH_2_ group, which connected with the imidazole moiety. Meanwhile, other triplet peaks in the ranges of *δ* 3.82–3.72 and 3.30–3.20 ppm were the signals of the O-CH_2_- (compounds **7a**–**7f** and **8a**–**8d**) and S-CH_2_- protons (compounds **9a**–**9d**), respectively. Specifically, the singlet peaks at *δ* 4.09–4.07 ppm on the spectra of compounds **8a**–**8d** were the protons of the CH_3_ group. The peaks in the range from *δ* 2.00–1.00 ppm, were for the protons of alkyl linker. In the ^13^C NMR spectra, the quartet (q) peaks at *δ* 130.9–129.6 and 119.5–119.0 ppm showed the carbon of CF_3_ and CCF_3_, respectively. Moreover, the characteristic peaks of pyrazol-C_4_ and C_5_ of compounds **8a**–**8d** and pyrazol-C_3_ and C_4_ in target compounds **7a**–**7f** and **9a**–**d** appeared at *δ* 139.0–138.8 and 109.2–108.2 ppm, respectively. The characteristic peaks of the imidazole moiety were signaled at *δ* 137.3–137.0, 129.5–129.2 and 118.9–118.0 ppm, respectively. Notably, two high peaks at *δ* ~130.0 and ~125.5 ppm in the spectra of target compounds **7a**–**7f** and **9a**–**d**, respectively, were validated as being the carbons of phenyl. The peaks that occurred at *δ* 48.0–26.0 ppm were mainly contributed by the signals of alkyl linker; distinctively, the CH_3_ of target compounds **8a**–**8d** were also signaled at this region ranging from *δ* 39.9 ppm to 39.8 ppm, and showed as a quartet. In ^19^F NMR spectra, the CF_3_ signals of compounds **7a**–**7f** and **9a**–**9d** mainly occurred at *δ* −55.8 or −55.7 ppm, whereas these CF_3_ signal appeared in the range from *δ* −58.0 ppm to −57.8 ppm for compounds **8a**–**8d**. Moreover, in HR(ESI)MS spectra, the exact molecular weights of all the synthesized target compounds were found according to the theoretical values, which stated that the designed molecules had been successfully synthesized. In addition, the purities of the active compounds were >97% by HPLC verification. The above-mentioned analysis declared that the title compounds were successful prepared, and characterization details can be found in the Supplementary Materials.

### 2.2. In Vitro Bioassays against Phytopathogenic Bacteria

#### 2.2.1. Preliminary In Vitro Bioassays

**Figure 2:**
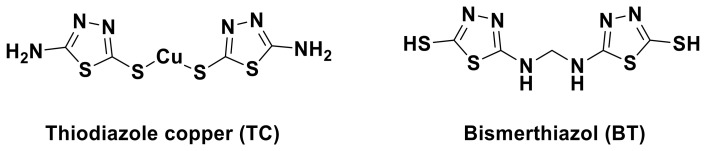
The chemical structure of commonly used bactericides bismerthiazol (**BT**) and thiodiazole copper (**TC**).

Generally, the target compounds were evaluated for their preliminary in vitro bioactivities against three devastating phytopathogenic bacteria (*Xoo*, *Xac* and *Psa*) via the turbidimetric test [39,40,41,42]. The commonly used bactericides bismerthiazol (**BT**) and thiodiazole copper (**TC**) (Figure 2) were chosen as positive controls [43,44]. As displayed in Table 1, most of the title compounds displayed remarkable antibacterial activity toward *Xoo* and *Xac* with complete inhibition of the growth of bacterial cells at 100 and 50 μg/mL, respectively. Notably, the inhibition rates were 100% for compounds **7a**, **7b**, **7c**, **7d**, **8c**, **8d**, **9a** and **9b** against *Xoo* and for compounds **7b**, **8b**, **8c** and **9a** against *Xac*. For *Psa*, most of the target compounds exhibited only moderate inhibitory activity, with inhibition rates mainly ranging from 46.87~78.45% and 40.21~65.87% at 100 and 50 μg/mL, respectively. In particular, compounds **7a**, **7b**, **8c** and **9a** provided relatively better inhibition rates of 56.93%, 63.90%, 61.51% and 65.87% at 50 μg/mL, respectively, which were better than those of the commercial bactericides thiodiazole copper (**TC**, 46.57%) and bismerthiazol (**BT**, 3.17%). These results indicated that most of the prepared imidazole derivatives showed more comfortable antibacterial activity than frequently used bactericides.

#### 2.2.2. The 50% Effective Concentration (EC_50_) Test

Furthermore, to assess the specific antibacterial activity, active compounds with inhibition rates over 50% at 50 μg/mL and the positive controls **BT** and **TC** were chosen to determine the EC_50_ via our previously reported method [39,40]. The bioassay (Table 2) results showed that most of the target compounds exhibited excellent antibacterial activity and high selectivity toward *Xoo*, affording active EC_50_ values of compounds **7a**, **7c**, **7d**, **7e**, **8d** and **9b** of 7.73, 7.40, 7.95, 8.78, 8.44 and 8.13 μg/mL, respectively, which were much higher than those of the commercial bactericides **TC** (76.81 μg/mL) and **BT** (31.94 μg/mL). Additionally, compounds **7b**, **8b**, **8c**, **9a** and **9c** also presented high anti-*Xoo* activity with EC_50_ values of 10.23, 20.25, 14.71, 12.40 and 27.26 μg/mL, respectively. Moreover, derivatives **8c** and **9a** showed outstanding antibacterial activity toward *Xac* with EC_50_ values of 8.72 and 5.44 μg/mL, which were better than those of **TC** (66.98 μg/mL) and **BT** (50.51 μg/mL), respectively. For anti-*Psa*, most of the title compounds afforded high EC_50_ values over 50 μg/mL, except for compounds **7a**, **7b**, **8c** and **9a**, which afforded moderate active EC_50_ values of 40.71, 28.40, 35.24 and 12.85 μg/mL, respectively. All these results demonstrated that most of the designed 2-(pyrazol-4-yl)-1,3,4-oxadiazoles showed moderate to good antibacterial activity against *Xoo*, *Xac* and *Psa*, affording the most active EC_50_ values of 7.40 (**7c**), 5.44 (**9a**) and 12.85 (**9a**) μg/mL, respectively, which were more effective than those of the commercial bactericides **BT** (31.94, 50.51 and 114.76 μg/mL, respectively) and **TC** (76.81, 66.98 and 74.98 μg/mL, respectively). Additionally, the prepared 2-(pyrazol-4-yl)-1,3,4-oxadiazoles possessed obvious selectivity for *Xanthomonas* (*Xoo* and *Xac*).

The EC_50_ values were also transformed from mass concentrations to molar concentrations, and a similar regularity in the bioactivity of the title compounds was performed. For instance, compound **7c** exhibited the lowest EC_50_ value of 16.55 μM toward *Xoo*, which was significantly better than that of **BT** (97.85 μM) and **TC** (234.16 μM). Furthermore, compound **9a** had outstanding bactericidal effects toward *Xac* and *Psa*, giving EC_50_ values of 11.76 and 27.75 μM, respectively, which were superior to **BT** (154.73 and 351.56 μM, respectively) and **TC** (204.21 and 228.59 μM, respectively).

In addition, according to the statistical analysis, the in vitro EC_50_ values showed significant differences toward all the tested pathogens between the active compounds and the positive controls (**BT** and **TC**). These outstanding results declared that the title compounds had great potential for controlling intractable phytopathogenic bacteria.

#### 2.2.3. Structure–Activity Relationship (SAR) Analysis

The preliminary SAR analysis showed that the antibacterial activity of 5-(1-phenyl-5-(trifluoromethyl)-1*H*-pyrazol-4-yl)-1,3,4-oxadiazole-2-ol derivatives decorated with an imidazole moiety **7a**~**7f** presented a fluctuation trend with the increase in the length of the alkyl linker from 4 to 12, affording the best EC_50_ values (7.40 and 11.22 μg/mL) toward *Xoo* and *Xac* when the carbon number of the alkyl linker was 6 (**7c**), respectively. For anti-*Psa*, compounds **7a** (*n* = 6) and **7b** (*n* = 8) showed moderate potencies, with EC_50_ values of 40.71 and 28.40 μg/mL, respectively, whereas the ability was sharply reduced when the alkyl linker was over 6 (compounds **7c**~**7f**) with EC_50_ values over 50 μg/mL. Thus, appropriate alkyl chain length was beneficial for enhancing antibacterial potency due to the title compounds possessing suitable hydrophobicity. All these results demonstrated that a short alkyl linker is more beneficial for bioactivity than a long alkyl linker, which suggested that the hydrophilicity of the compound had a significant effect on the bioactivity. These molecules might interact with the cell membrane via electrostatic interactions and enter inside the bacterial cell via the endocytosis. Then hydrophobic fragments (alkyl chain) would penetrate the bacterial membrane, disrupt the function of the cell membranes (such as the permeability, etc.) and cause the leakage of intracellular material, thereby leading to the death of bacterial cells. This possible hypothesis is proposed referring to the previous study [45].

To investigate the effect of the position (R_1_) and variety (R_2_) of the pyrazole ring on the antibacterial ability, four (1-methyl-3-(trifluoromethyl)-1*H*-pyrazol-4-yl)-1,3,4-oxadiazol-2-ol derivatives decorated with an imidazole moiety **8a**~**8d** were synthesized based on target compounds **7c**~**7f**. As shown in Table 1 and Table 2, the antibacterial competence on anti-*Xac* was sharply decreased when the site of the -CF_3_ group was transferred from 5- to 3- and the phenyl was replaced by methyl, such as compound **7c** (EC_50_ = 11.22 μg/mL, *n* = 6) > compound **8a** (EC_50_ > 50 μg/mL, *n* = 6). Similarly, the same results were summarized for compounds **8b**~**8d** against *Xoo* when the length of the alkyl linker was increased from 8 to 12, which indicated that the 5-(1-phenyl-5-(trifluoromethyl)-1*H*-pyrazol-4-yl)-1,3,4-oxadiazole-2-ol moiety was more favorable for enhancing the anti-*Xoo* ability than the 1-methyl-3-(trifluoromethyl)-1*H*-pyrazol-4-yl)-1,3,4-oxadiazol-2-ol moiety. The above findings stated that the increased lipophilic property of R_2_ was of more benefit for the bioactivity than that of a low lipophilic group. Meanwhile, when the alkyl length was *n* = 8, 10 and 12 (compounds **8b**~**8d**), a partly enhanced antibacterial potency appeared toward *Xac* and *Psa*, compared to that of the relative compounds **7d**~**7f**, with improved EC_50_ values ranging from 11.22 and > 50 μg/mL to 8.72 and 35.24 μg/mL, respectively. These results suggest that the position (R_1_) and variety (R_2_) of the substituent on the pyrazole ring have an indeterminate influence on the bactericidal activity, which also relates to the carbon number of the alkyl linker.

To investigate the effect of the O atom of the 1,3,4-oxadiazol-2-ol moiety on the antibacterial potential, four imidazole-tailed 5-(1-phenyl-5-(trifluoromethyl)-1*H*-pyrazol-4-yl)-1,3,4-oxadiazole-2-thiol derivatives **9a**~**9d** were synthesized based on target compounds **7c**~**7f**. As shown in Table 1 and Table 2, a depressed potency against *Xoo* was observed when the O atom was replaced by the S atom, especially the compounds with the alkyl chain of *n* equals 6 and 10, e.g., **7c** (EC_50_ = 7.40 μg/mL, *n* = 6) > **9a** (EC_50_ = 12.40 μg/mL, *n* = 6) and **7e** (EC_50_ = 8.78 μg/mL, *n* = 10) > **9c** (EC_50_ = 27.26 μg/mL, *n* = 10), respectively; and the comparable activities were displayed between **7d** (EC_50_ = 7.95 μg/mL, *n* = 8) and **9b** (EC_50_ = 8.13 μg/mL, *n* = 8), **7f** (66.30% at 100 μg/mL, *n* = 12) and **9d** (58.57% at 100 μg/mL, *n* = 12), respectively, indicating that the O atom on the 1,3,4-oxadiazol-2-ol moiety was more beneficial for exerting anti-*Xoo* activity. For anti-*Xac* and anti-*Psa*, the enhanced antibacterial power of the S-atom-containing compound **9a** (EC_50_ values were 5.44 and 12.85 μg/mL, respectively) was observed, compared to the relative O-atom-containing compound **7c** (EC_50_ values were 11.22 and >50 μg/mL, respectively) when the alkyl length was *n* = 6, but the EC_50_ values were increased when the alkyl lengths were 8, 10 and 12. Overall, it is worth noting that a globally reduced tendency in the antibacterial potential of compounds **9a**~**9d** with an increase in the alkyl length from 6 to 12 showed that the decrease in molecular water solubility was unfavorable to bioactivity; also, the S and O atoms presented an uncertainly effect on the bioactivities toward different bacterial species.

Furthermore, these EC_50_ values were transformed from mass concentrations to molar concentrations. The antibacterial activities of compounds **7a**~**7f** also presented an undulant trend when the length of the alkyl linker increased from 4 to 12, affording their active EC_50_ values that ranged from 16.55 to >100 μM toward *Xoo*, 25.09 to >100 μM toward *Xac* and 65.56 to >100 μM toward *Psa*, respectively. This indicates that the too-long length of alkyl linkers was unfavorable to increase the antibacterial competence, e.g., the compound **7f** (*n* =12) provided EC_50_ values over 100 μM toward all the tested bacteria. In addition, the antibacterial competence on anti-*Xac* and *Xoo* was sharply decreased when the position of the -CF_3_ group was transferred from 5- to 3- and the phenyl was replaced by methyl, such as compound **7c** (EC_50_ = 16.55 and 25.09 μM, respectively, *n* = 6) >compound **8a** (EC_50_ were over 100 μM, *n* = 6). However, the significantly improved potency toward *Xac* was observed when the alkyl linker was *n* = 8 and 10, e.g., **7d** (EC_50_ > 100 μM, *n* = 8) < **8b** (EC_50_ = 26.40 μM, *n* = 8), and **7e** (EC_50_ = 72.79 μM, *n* = 10) < **8c** (EC_50_ = 19.76 μM, *n* = 10). As for anti-*Psa*, compound **8c** showed the enhanced power with the moderate EC_50_ value of 79.87 μM. The results suggested that the position (R_1_) and variety (R_2_) of the substituent on the pyrazole ring and the length of alkyl linker possessed an uncertain influence on the bactericidal activity. Finally, a globally reduced tendency against *Xoo* was observed when the O atom was replaced by the S atom, especially the compounds with the alkyl chain of *n* equals 6 and 10 where the activity decreased significantly, e.g., **7c** (EC_50_ = 16.55 μM, *n* = 6) < **9a** (EC_50_ = 26.77 μM, *n* = 6), and **7e** (EC_50_ = 17.46 μM, *n* = 10) < **9c** (EC_50_ = 52.51 μM, *n* = 10). In addition, the compounds possessed an equivalent activity in the alkyl chain of 8 (**7d** and **9b**). For anti-*Xac* and anti-*Psa* activities, the S-atom-containing compound **9a** (EC_50_ values were 11.76 and 27.75 μM, respectively) displayed enhanced antibacterial power to the relative O-atom-containing compound **7c** (EC_50_ values were 25.09 against *Xoo* and >100 μM toward *Psa*, respectively) with the alkyl length of *n* = 6, but the bioactivities were sharply weakened with the increased alkyl lengths, which declared that the decreased water solubility of the molecule was unfavorable to bioactivity, and the S and O atom possessed an uncertainly effect on the bioactivities. 

The ADME properties of compounds **7c** and **9a** were assessed by using the ADMETlab 2.0 software [46]. The predicted results are displayed in Table S2; these two compounds had acceptable physicochemical properties, ADMET, and drug-like properties. For instance, compounds **7c** and **9a** had better safety in some aspects (such as AMES toxicity, eye corrosion, eye irritation, etc.). Interestingly, compounds **7c** and **9a** were shown to meet the Lipinski rule and Golden Triangle. These results suggest that these two compounds possess good pharmacokinetic characteristics for new agrochemical discovery.

### 2.3. In Vivo Bioassays of Compound **7c** against Rice Bacterial Blight 

**Figure 3:**
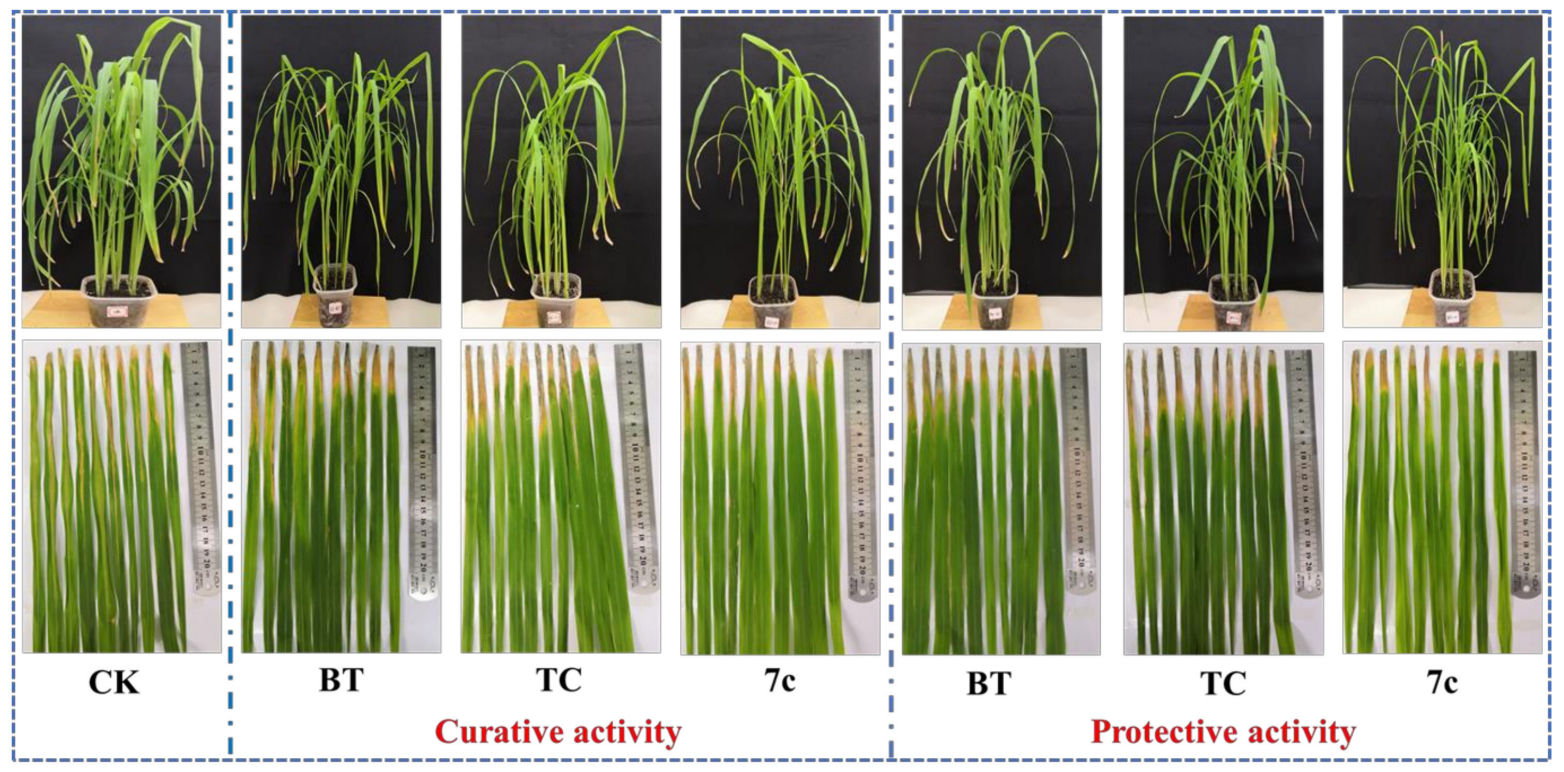
In vivo antibacterial activities of compound **7c** against rice bacterial blight at 200 μg/mL via pot experiments. **BT** and **TC** were used as positive controls under the same conditions.

Based on the in vitro bioassays, the active compound **7c** (the lowest EC_50_ value of 7.40 μg/mL toward *Xoo*) was chosen to evaluate the in vivo antibacterial effects against rice bacterial blight via the pot experiment [39]. The results (Figure 3 and Table 3) showed that compound **7c** possessed good in vivo protective and curative activities to manage rice bacterial leaf blight at 200 μg/mL, with control efficacies of 47.34% and 41.18%, respectively, which were much better than those of the commercial bactericide **TC** (35.12% and 37.50%, respectively) and partly superior to **BT** (48.28% and 31.37%, respectively). In particular, the designed compounds showed low phytotoxicity toward plants due to the compounds not causing any lesions or necrosis on the rice leaves and stem. These results indicate that compound **7c** possesses promising applications for controlling rice bacterial blight and could be considered as a lead molecule to develop novel agricultural bactericides.

### 2.4. In Vivo Bioassays of Compound **9a** against Kiwifruit Bacterial Canker

**Figure 4:**
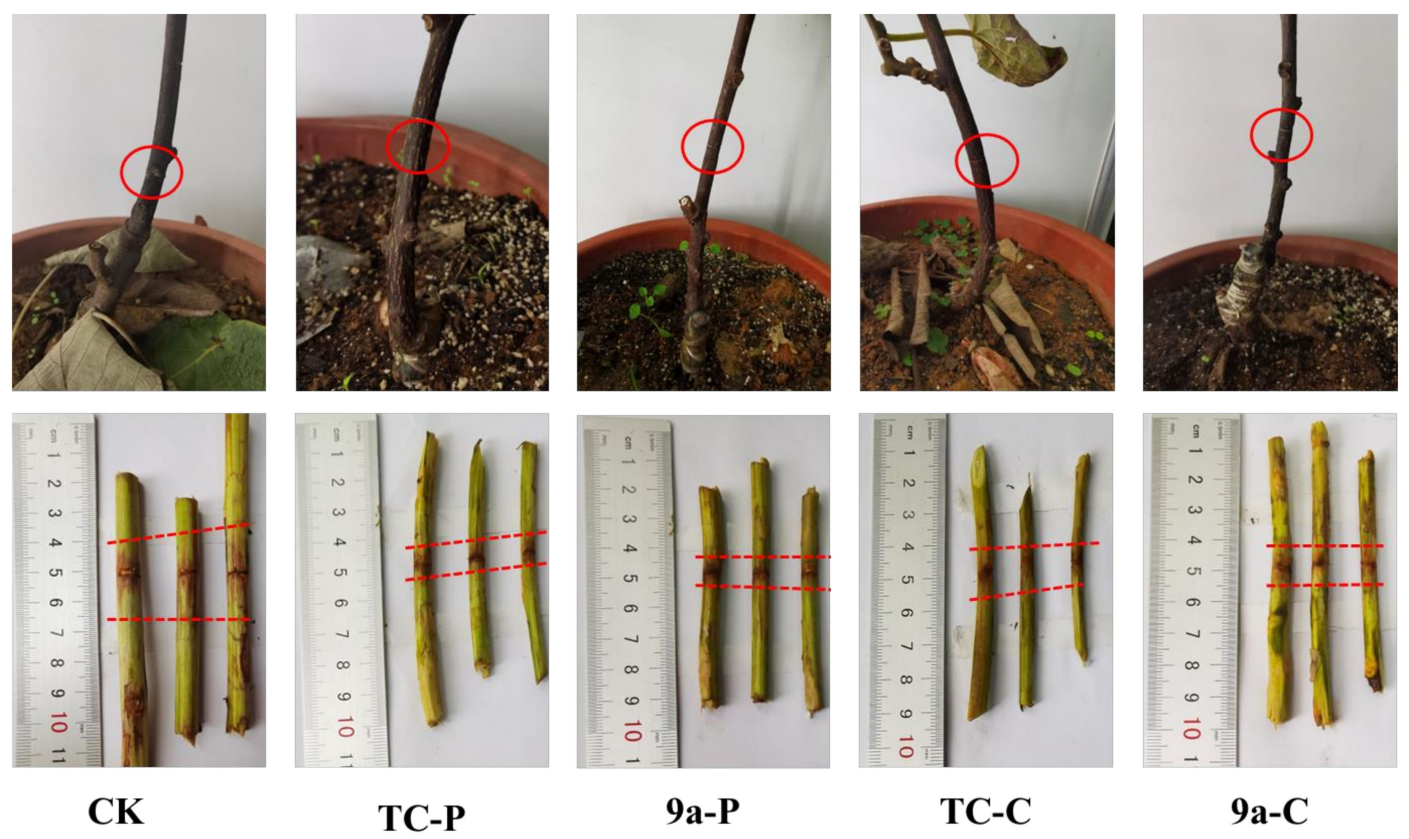
In vivo antibacterial activities of compound **9a** against kiwifruit bacterial canker at 200 μg/mL via pot experiments. **TC** was used as the positive control under the same conditions. The gray areas between the red lines are the scab of kiwifruit bacterial canker.

Based on the in vitro bioassays, the active compound **9a** (afforded with the lowest EC_50_ value of 12.85 μg/mL toward *Psa*) was chosen to assess the in vivo anti-*Psa* activity via the pot experiment [47,48,49]. The results (Table 4) showed that compound **9a** presented commendable protective and curative activities against kiwifruit bacterial canker at 200 μg/mL with control efficiencies of 46.05% and 32.89%, respectively, which were much better than those of the commercial bactericide **TC** (31.58% and 17.11%, respectively). For the observations at 14 days after inoculation (Figure 4), severe blackening with pyogenic exudate was observed on the wounds of negative controls (red circle). In contrast, only a little white exudate was discovered around the wounds without signs of obvious deterioration after treatment with compounds **9a** and **TC**, which indicated that our designed molecular skeleton was a promising core to develop a novel bactericidal agent for controlling kiwifruit bacterial canker.

### 2.5. Growth Effect of Compound **7c** toward Xoo

**Figure 5:**
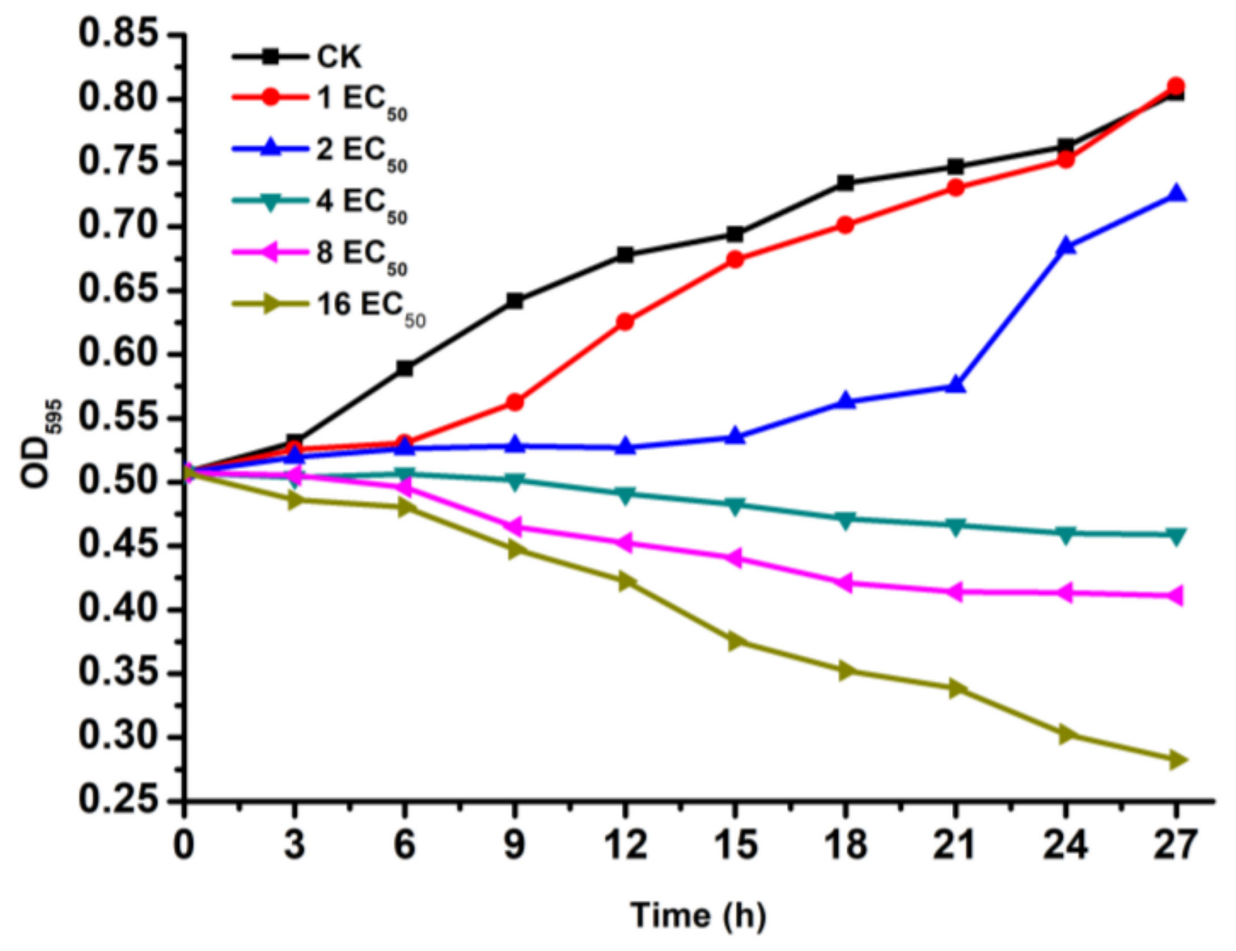
The growth of *Xoo* (initial OD_595_ = 0.5) effected by compound **7c** at the concentrations of 1 × EC_50_ (7.40 μg/mL), 2 × EC_50_ (14.80 μg/mL), 4 × EC_50_ (29.60 μg/mL), 8 × EC_50_ (59.20 μg/mL) and 16 × EC_50_ (118.20 μg/mL), respectively. The optical density of the cultures was monitored by detecting OD_595_.

To investigate the probable action mechanism of the designed molecules, the growth effect assay was carried out according to the previously reported method with some modifications [50]. The results (Figure 5) displayed that the bacterial growth was slightly restrained after treatment with 1 EC_50_ and 2 EC_50_ of compound **7c** at an early stage (0–12 h), whereas a rapid increase growth rate was observed after 12 h and kept similar OD_595_ values with the CK after 24 h. However, after incubating with the 4 EC_50_ compounds, the growth curve showed a significant downward trend. All these findings suggest that compound **7c** showed a bacteriostatic effect toward *Xoo* at the low concentrations (<4 EC_50_), whereas a bactericidal effect was displayed at the high dosages (>4 EC_50_).

### 2.6. Morphological Observation of Xoo Cells by Scanning Electron Microscopy (SEM)

**Figure 6:**
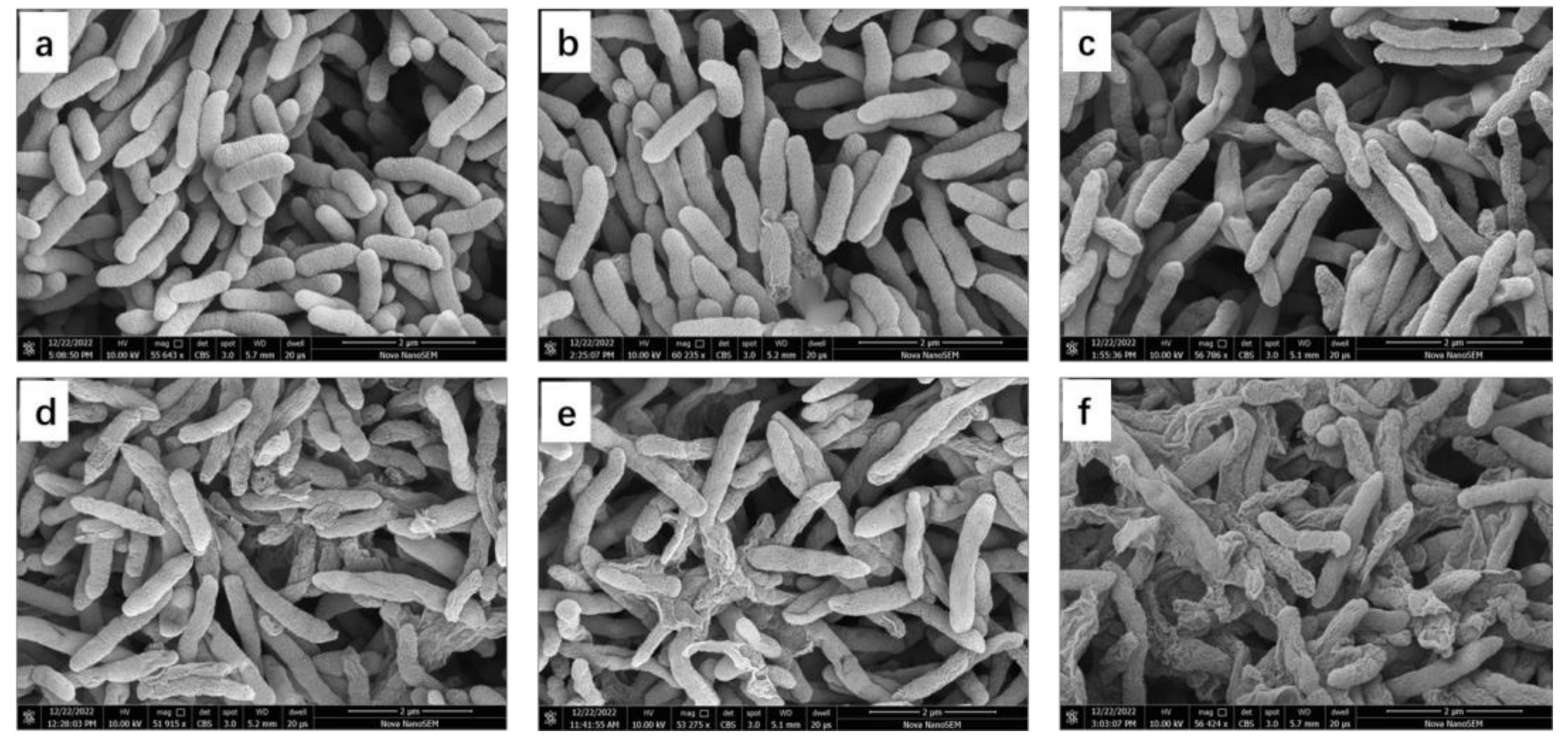
SEM images of *Xoo* after incubation with different concentrations of compound **7c**. (**a**) CK (0 μg/mL); (**b**) 1 × EC_50_ (7.40 μg/mL); (**c**) 2 × EC_50_ (14.80 μg/mL); (**d**) 4 × EC_50_ (29.60 μg/mL); (**e**) 8 × EC_50_ (59.2 μg/mL); and (**f**) 16 × EC_50_ (118.2 μg/mL). Scale bars are 2 μm.

To observe the morphological changes and cell membrane integrity of phytopathogens after treatment with our synthesized compounds, SEM technology was employed according to our reported method [40]. As displayed in Figure 6, the morphology and cell membrane integrity of *Xoo* cells were obviously affected after incubation with compound **7c** for 15 h. Clearly, the cells in the negative controls had a full surface with a uniform, complete and regular shape. Comparatively, partial collapse and shrinking occurred in small amounts of *Xoo* cells when treated with a low concentration of compound **7c** at 7.40 μg/mL (1 × EC_50_) (Figure 6b), and more serious damage was observed at the increased concentrations (Figure 6c–e), with most of the cells collapsing, shrinking, distorting and flattening at 14.80 μg/mL (2 × EC_50_), 29.60 μg/mL (4 × EC_50_) and 59.2 μg/mL (8 × EC_50_). In particular, almost all *Xoo* cells were severely damaged at the drug dose of 118.2 μg/mL (16 × EC_50_) (Figure 6f), with the morphology changing to one of shrinking, collapsing and breaking, and leakage holes appearing on the surface of the majority of the *Xoo* cell membrane. All these observations indicate that compound **7c** has a strong impact on the morphology and cell membrane of *Xoo* cells.

### 2.7. Membrane Permeability Changes by Propidium Iodide (PI) Staining Experiment

**Figure 7:**
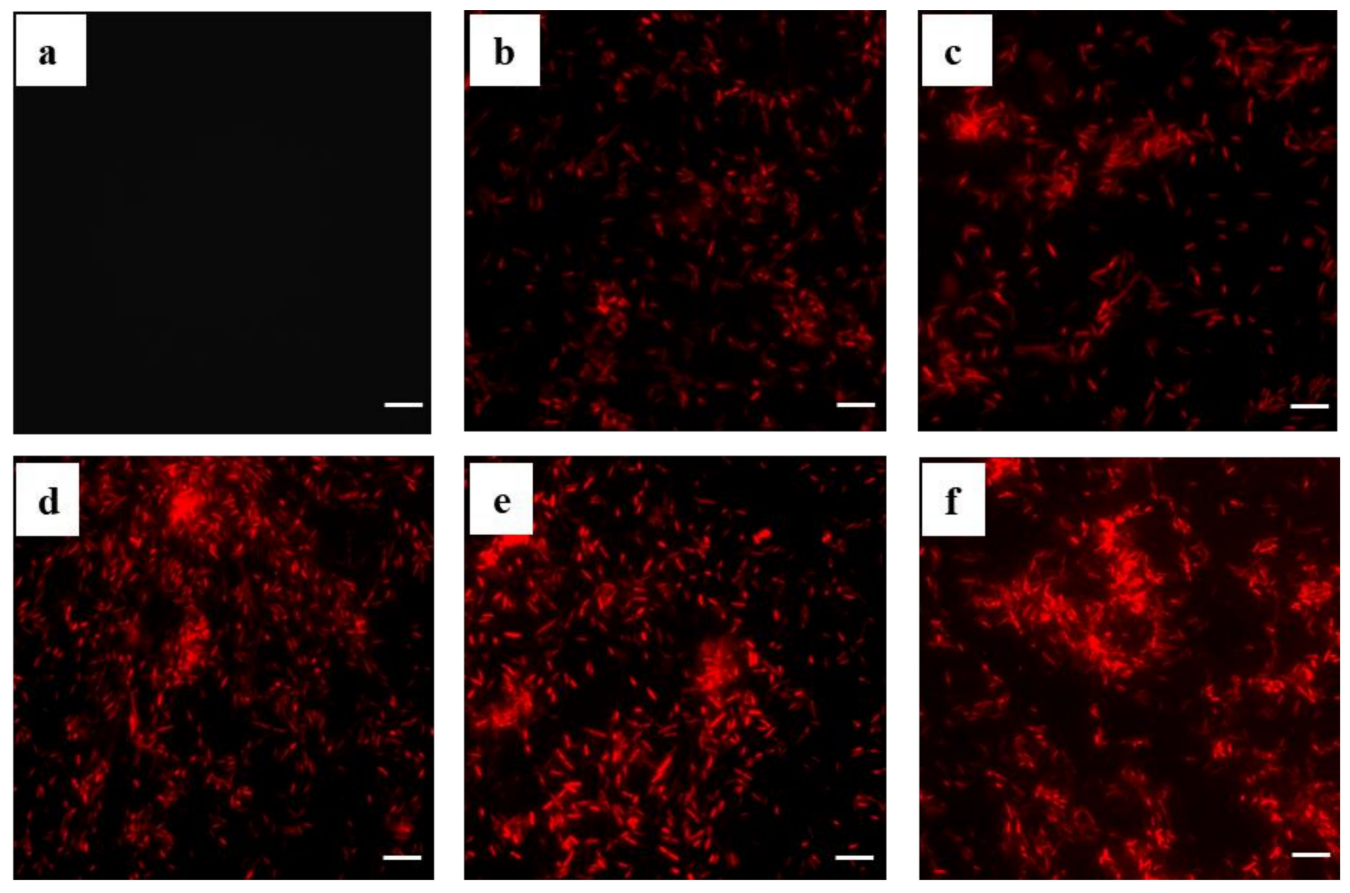
Membrane permeability changes in *Xoo* cells by propidium iodide (PI) staining assay after treatment with different concentrations of compound **7c** for 15 h. (**a**) CK (0 μg/mL); (**b**) 1 × EC_50_ (7.40 μg/mL); (**c**) 2 × EC_50_ (14.80 μg/mL); (**d**) 4 × EC_50_ (29.60 μg/mL); (**e**) 8 × EC_50_ (59.2 μg/mL); and (**f**) 16 × EC_50_ (118.2 μg/mL). Scale bars are 10 *μ*m. All images were captured on an Olympus BX53 microscope (Olympus, Tokyo, Japan).

Permeability of the cell membrane has important physiological functions for the movement of water inside and outside the cell, the exchange of various substances, and the maintenance of pH and osmotic pressure [51,52]. To study the membrane permeability of the tested phytopathogenic bacteria affected by our designed compounds, the *Xoo* cells were detected by using a typical PI staining assay [53], in which the nonfluorescent dye PI can bind to DNA and RNA in cells with a damaged cell membrane and produce strong red fluorescence, but it cannot pass through cells with intact cell membranes, so the intensity and distribution of red fluorescence indirectly reflects the membrane permeability [54]. Clearly, the progressively elevated red fluorescence intensity and increased number of fluorescent cells (Figure 7b–f) showed that enhanced membrane permeability of *Xoo* cells occurred after incubation with compound **7c** for 15 h when compared with the cells in the negative control (Figure 7a). All these findings demonstrate that our designed 2-(pyrazol-4-yl)-1,3,4-oxadiazoles could change the cell membrane permeability of *Xoo* and cause the dysfunction of cell metabolism, which might be a key factor in revealing outstanding antibacterial potency. All these findings are in accordance with the outcomes of SEM observation.

## 3. Materials and Methods

### 3.1. Instruments and Chemicals

NMR spectra were collected on a 400 MHz Bruker Biospin-AG-400 instrument (BRUKER OPTICS, Fällanden, Switzerland) or a 500 MHz JEOLECX-500 instrument (JEOL, Tokyo, Japan), the CDCl_3_ and TMS were used as the solvent and internal standard, respectively, as well parts per million (ppm) and Hz represented chemical shifts and coupling constants (*J*), respectively. High resolution mass spectrometry (HRMS) spectra were recorded on Thermo Scientific Q Exactive (UItiMate 3000, Thermo Scientific, Waltham, MA, USA); the methanol (LC-MS, 99.9%) and electrospray ionization (ESI) in positive ion mode were used as the solvents and ionization, respectively. Scanning electron microscopy (SEM) images were produced via an FEI Nova NanoSEM 450 (FEI, Hillsboro, OR, USA). Fluorescent images were taken on Olympus BX53 microscope (Olympus, Japan). Optical density (OD) was detected on a Cytation™5 multi-mode reader (BioTek Instruments, Inc., Winooski, VT, USA). High performance liquid chromatography (HPLC) was performed on an Agilent 1260 InfinityⅡinstrument (Agilent Technologies Inc., Santa Clara, CA, USA); the chemicals ethyl 4,4,4-trifluoroacetoacetate (98%), phenylhydrazine (98%), methylhydrazinium sulphate (98%), *N*, *N*’-Carbonyldiimidazole (CDI, 98%), imidazole (99%) and 1, n-dibromo-substituted alkane (97%~99%) were purchased from energy chemical of Sahn Chemical Technology (Shanghai) Co., LTD (Shanghai, China); carbon disulfide (>99.9%) was obtained from Shanghai Aladdin Biochemical Technology Co., LTD (Shanghai, China); the other reagents of absolute ethyl alcohol, acetic acid, tetrahydrofuran, dichloromethane were accessed from commercial resources in analytically pure state.

### 3.2. Synthesis

#### 3.2.1. General Synthetic Protocols for Target Compounds **7a**~**7f**, **8a**~**8d** and **9a**~**9d**

As depicted in Scheme 1, the intermediates **2**–**6** were obtained according to our previously reported methods [37,38]. For the details, see Supplementary Materials.

#### 3.2.2. General Synthetic Protocols for Target Compounds **7a**~**7f**, **8a**~**8d** and **9a**~**9d**

Intermediate compound 5-(1-phenyl-5-(trifluoromethyl)-1*H*-pyrazol-4-yl)-1,3,4-oxadiazol-2-ol (**5A**), [5-(1-methyl-3-(trifluoromethyl)-1*H*-pyrazol-4-yl)-1,3,4-oxadiazol-2-ol (**5B**) or 5-(1-phenyl-5-(trifluoromethyl)-1*H*-pyrazol-4-yl)-1,3,4-oxadiazole-2-thiol (**6**)] (1.01 mM), NaOH (1.50 mM) and DMF (10.0 mL) were stirred at room temperature for 20 min. Then, the corresponding dibromo alkane (1.41 mM) was slowly dropped into the reaction system and continually reacted at room temperature for another 2 h. After that, the reaction was diluted with ethyl acetate, washed by saturated ammonium chloride solution, dried using anhydrous sodium sulfate and evaporated under vacuum. Subsequently, the crude product was added into a mixture of imidazole (1.00 mM), NaH (1.20 mM) and DMF (2 mL) under ice bath condition, and reacted at room temperature for 4 h. After that, the reaction was diluted with ethyl acetate, washed by saturated ammonium chloride solution, dried by anhydrous sodium sulfate and evaporated under vacuum. Finally, the target compounds were purified by column chromatography on a silica gel using CH_2_Cl_2_ and CH_3_OH (30:1) as the eluant to afford the desired products **7a**~**7f**, **8a**~**8d** and **9a**~**9d**, respectively. All the NMR and HRMS spectra for target compounds are displayed in the Supplementary Materials.

### 3.3. Bioassays

#### 3.3.1. Methods for General Bioassays

The in vitro bioassays against three phytopathogenic bacteria *Xoo*, *Xac* and *Psa*, in vivo pot experiment for managing rice bacterial leaf blight and SEM imaging experiment were according to our reported methods [39,40,41,42]. All the detailed descriptions are displayed in Supplementary Materials.

#### 3.3.2. In Vivo Antibacterial Bioassay of Compound **9a** against Kiwifruit Bacterial Canker

The in vitro bioassays of the active compound **9a** against kiwifruit bacterial canker were carried out according to our reported method with some modifications [45,46,47]. The commercial bactericide thiodiazole copper (**TC**, 20% suspending agent) and an equivalent DMSO were used as the positive and negative (CK) controls, respectively. Briefly, the healthy kiwifruit pot with smooth surface were cleaned up using a degreasing cotton soaked with water. Then, three wounds were made with 1 mm width and down to xylem using a sterilized knife on each plant. For the protective assay, 10 μL of drug solution (200 μg/mL) or DMSO solution were added into the corresponding wounds, then 10 μL of Psa bacterial suspension (OD_595_ = 0.1) was inoculated at 24 h after addition. For the curative assay, there was only a time change on adding drug solution and *Psa* bacterial suspension. After 30 min for each operation, all the treatments were cultured in a climate chamber (95% RH) under 14 h lighting at 14 °C and 10 h dark at 10 °C. The length of lesion was measured 14 days after inoculation. All treatments were carried out in triplicate. The control efficiencies (*I*) were calculated by the following equation:

Corrected lesion length (cm) = measured lesion length − 1.0

Control efficiency *I* (%) = (C − T)/C × 100

In the equation, C and T are the average corrected length of lesion of the negative control and the treatment group, respectively.

#### 3.3.3. Growth Effect Assay of Compound **7c** against Xoo

To further know the probable action of the designed molecules, we performed an in vitro growth curve assay on *Xoo* via the previously reported method with some modifications [50]. Firstly, some bacterial colony was incubated into fresh NB broth at 28 °C. After overnight growth, the cultures were adjusted to the OD_595_ value of ~0.5 by sterile NB broth, then 200 *μ*L of the adjusted *Xoo* solutions was added into a 96-well plate and supplemented with compound 7c at concentrations of 1 EC_50_, 2 EC_50_, 4 EC_50_, 8 EC_50_ and 16 EC_50_, respectively. The plates only containing the adjusted *Xoo* solutions were used as the blank controls. After that, the samples were incubated in a Cytation™ 5 multi-mode readers at 28 °C for 27 h, and the OD_595_ values were detected every 3 h. Finally, the growth curve was drawn by using origin 8.

#### 3.3.4. Membrane Permeability Changes by Propidium Iodide (PI) Staining Experiment

In this assay, the method was according to the guidebook of a commercial PI staining kit. Briefly, compound **7c** was added into 2 mL *Xoo* solution with OD_595_ of 0.2 to give the final concentrations of 1 × EC_50_ (7.40 μg/mL), 2 × EC_50_ (14.80 μg/mL), 4 × EC_50_ (29.60 μg/mL), 8 × EC_50_ (59.2 μg/mL) and 16 × EC_50_ (118.2 μg/mL), respectively, and the equivalent volume of DMSO was used as blank control. Then, all the treatments were incubated in a constant temperature shaker under the conditions of 28 °C and 180 rpm for 14 h. After that, all the samples were washed with PBS (10 mM, pH = 7.2) 3 times and resuspended in 100 μL PBS. Subsequently, the bacterial cells were stained with 10 μL PI stain (20 μg/mL) for 30 min, and then washed by PBS 2 times to remove the extracellular stain. Finally, all the treated *Xoo* cells were visualized on an Olympus BX53 microscope under red fluorescent channel with exposure time of 2 s.

#### 3.3.5. Data and Statistical Analysis

The data of in vitro antibacterial assay were analyzed by using excel 2013 (Microsoft Corporation, Washington, DC, USA) and shown as the average mean ± standard deviation (SD) of three replicate data [55]; the detailed processes are described in Supplementary Materials. In addition, Duncan’s multiple range test of one-way analysis of variance (ANOVA, *p* = 0.05) was conducted in SPSS ver. 25.0 statistical software (SPSS Inc., Chicago, IL, USA) for statistical analysis, and the EC_50_ values and in vivo bioassay results of different treatments were considered statistically significant when *p*-value was < 0.05 [56].

## 4. Conclusions

In summary, fourteen novel 2-(pyrazol-4-yl)-1,3,4-oxadiazoles decorated with an imidazole moiety were designed and synthesized via a four-step reaction, and their structures were identified by ^1^H NMR, ^13^C NMR, ^19^F NMR and HRMS. The in vitro bioassays revealed that most of the target compounds showed moderate to good antibacterial activity against three intractable phytopathogenic bacteria, *Xoo*, *Xac* and *Psa*, with the lowest EC_50_ values of 7.40 (**7c**), 5.44 (**9a**) and 12.85 (**9a**) μg/mL, respectively, which were more powerful than those of the commercial bactericides **BT** (31.94, 50.51 and 114.76 μg/mL, respectively) and **TC** (76.81, 66.98 and 74.98 μg/mL, respectively). The structure–activity relationship (SAR) analysis showed that the alkyl chain, the position (R_1_) and variety (R_2_) of the substituent on the pyrazole ring, and the S and O atom on 1,3,4-oxadiazole ring presented crucial effects on the bioactivities. Furthermore, the pot experiments showed that compound **7c** possessed good in vivo protective and curative activities to manage rice bacterial leaf blight at 200 μg/mL, with control efficacies of 47.34% and 41.18%, respectively, which were much better than those of the commercial bactericide **TC** (35.12% and 37.50%, respectively) and partly superior to **BT** (48.28% and 31.37%, respectively). Meanwhile, compound **9a** presented commendable in vivo protective and curative activities against kiwifruit bacterial canker at 200 μg/mL, with control efficiencies of 46.05% and 32.89%, respectively, which were much better than those of the commercial bactericide **TC** (31.58% and 17.11%, respectively). Finally, the growth effect assay, SEM observations and PI staining experiment mutually verified that these designed molecules could negatively impact the cell membrane of phytopathogenic bacteria cells and cause the leakage of the intracellular component, thereby leading to the killing of bacteria. All these findings indicate that the imidazole-decorated 2-(pyrazol-4-yl)-1,3,4-oxadiazoles **7c** and **9a** can be considered as lead molecules to develop novel agricultural bactericides.

## Figures and Tables

**Table 1:** Inhibition effect (%) of target compounds **7a**~**7f**, **8a**~**8d** and **9a**~**9d** against phytopathogenic bacteria *Xoo*, *Xac*, and *Psa*.

Compd.	*Xoo* ^a^		*Xac* ^a^		*Psa* ^a^	
	100 μg/mL	50 μg/mL	100 μg/mL	50 μg/mL	100 μg/mL	50 μg/mL
**7a**	100	100	100	88.54 ± 1.24	76.40 ± 2.59	56.93 ± 2.47
**7b**	100	100	100	100	70.20 ± 3.23	63.90 ± 3.38
**7c**	100	100	90.59 ± 1.03	86.44 ± 0.77	62.94 ± 3.47	46.51 ± 0.21
**7d**	100	100	86.13 ± 0.43	49.72 ± 0.57	59.89 ± 2.43	44.99 ± 0.29
**7e**	80.73 ± 3.50	71.90 ± 1.21	82.41 ± 2.21	67.68 ± 1.67	49.47 ± 2.50	43.84 ± 3.02
**7f**	66.30 ± 3.57	24.45 ± 3.97	22.29 ± 1.67	16.78 ± 0.81	59.31 ± 1.74	45.85 ± 2.17
**8a**	21.34 ± 1.73	0	46.44 ± 3.47	36.41± 6.33	47.56 ± 0.76	26.84 ± 2.75
**8b**	100	78.27 ± 1.09	100	100	72.49 ± 2.23	44.98 ± 1.62
**8c**	100	100	100	100	72.64 ± 1.02	61.51 ± 0.21
**8d**	100	100	67.18 ± 2.90	38.95 ± 2.61	64.18 ± 4.45	47.85 ± 3.38
**9a**	100	100	100	100	78.45 ± 3.72	65.87 ± 1.25
**9b**	100	100	88.05 ± 1.78	87.37 ± 2.41	46.13 ± 0.20	16.71 ± 2.43
**9c**	55.70 ± 4.40	60.88 ± 0.33	19.69 ± 1.02	18.95 ± 1.54	54.87 ± 0.20	40.21 ± 3.58
**9d**	58.57 ± 2.41	27.03 ± 3.96	45.94 ± 0.17	39.07 ± 0.93	37.07 ± 1.96	32.66 ± 4.86
**TC**	51.04 ± 2.14	38.81 ± 2.25	57.94 ± 2.67	36.17 ± 0.30	57.31 ± 0.80	46.57 ± 1.99
**BT**	100	100	99.84 ± 0.27	49.45 ± 2.16	24.73 ± 0.23	3.17 ± 3.12

^a^ The inhibition rates were expressed as average values ± standard deviation (SD) of three replicate data.

**Table 2:** EC_50_ values of target compounds **7a**~**7f**, **8a**~**8d** and **9a**~**9d** against *Xoo*, *Xac* and *Psa*.

Comp.	*Xoo*		*Xac*		*Psa*	
	EC_50_ (μg/mL) ^a^	EC_50_ (μM) ^a^	EC_50_ (μg/mL)	EC_50_ (μM)	EC_50_ (μg/mL)	EC_50_ (μM)
**7a**	7.73 ± 1.25 f	18.44 ± 1.51 e	41.69 ± 0.59 c	99.47 ± 1.42 c	40.71 ± 0.09 c	97.13 ± 0.21 c
**7b**	10.23 ± 0.20 def	23.61 ± 0.46 de	30.26 ± 1.85 d	69.87 ± 4.28 d	28.40 ± 1.53 d	65.56 ± 3.90 d
**7c**	7.40 ± 1.26 f	16.55 ± 2.81 e	11.22 ± 0.09 e	25.09 ± 0.19 e	>50	>100
**7d**	7.95 ± 0.12 f	16.72 ± 0.27 e	>50	>100	>50	>100
**7e**	8.78 ± 0.31 ef	17.46 ± 0.70 e	36.63 ± 0.71 cd	72.79 ± 1.42 d	>50	>100
**7f**	>50	>100	>50	>100	>50	>100
**8a**	>50	>100	>50	>100	>50	>100
**8b**	20.25 ± 0.41 c	49.01 ± 0.99 c	10.91 ± 0.36 e	26.40 ± 0.84 e	>50	>100
**8c**	14.71 ± 0.22 d	33.33 ± 0.50 d	8.72 ± 1.20 e	19.76 ± 1.00 e	35.24 ± 1.58 cd	79.87 ± 3.58 cd
**8d**	8.44 ± 0.54 ef	17.99 ± 0.78 e	>50	>100	>50	>100
**9a**	12.40 ± 0.13 de	26.77 ± 0.25 de	5.44 ± 0.39 e	11.76 ± 0.65 e	12.85 ± 1.42 e	27.75 ± 3.07 e
**9b**	8.13 ± 0.51 f	16.56 ± 0.72 e	10.84 ± 0.32 e	22.08 ± 0.83 e	>50	>100
**9c**	27.26 ± 1.65 b	52.51 ± 3.17 c	>50	>100	>50	>100
**9d**	>50	>100	>50	>100	>50	>100
**TC**	76.81 ± 2.22 a	234.16 ± 6.76 a	66.98 ± 0.49 a	204.21 ± 1.49 a	74.98 ± 3.49 b	228.59 ± 10.64 b
**BT**	31.94 ± 3.59 b	97.85 ± 4.25 b	50.51 ± 2.08 b	154.73 ± 6.35 b	114.76 ± 3.93 a	351.56 ± 12.03 a

^a^ The EC_50_ values were expressed as average values ± standard deviation (SD) of three replicate data. The statistical analysis was conducted by ANOVA under the condition of equal variances assumed (*p* > 0.05) and equal variances not assumed (*p* < 0.05). Different lowercase letters indicate the control efficiencies with significant differences among different treatment groups at *p* < 0.05. The toxic regression equation and correlation coefficient (R^2^) are displayed in Supplementary Materials.

**Table 3:** In vivo control efficiencies (14 days after inoculation) of compound **7c** against rice bacterial blight at 200 μg/mL.

Chemicals	Protective Activity			Curative Activity		
	Morbidity (%)	Disease Index (%)	Control Efficiency (%) ^a^	Morbidity (%)	Disease Index (%)	Control Efficiency (%) ^a^
**7c**	100	39.78	47.34 a	100	44.44	41.18 a
**BT**	100	39.08	48.28 a	100	51.85	31.37 b
**TC**	100	49.02	35.12 b	100	47.22	37.50 a
**CK**	100	75.56		100	75.56	

^a^ Statistical analysis was conducted by ANOVA under the condition of equal variances assumed (*p* > 0.05) and equal variances not assumed (*p* < 0.05). Different lowercase letters indicate the control efficiencies with significant differences among different treatment groups at *p* < 0.05.

**Table 4:** In vivo control efficiencies (14 days after inoculation) of compound **9a** toward kiwifruit bacterial canker at 200 μg/mL.

Chemicals ^a^	Measured Lesion Length (mm)			Corrected Lesion Length (mm)			Control Efficiency (%) ^b^
**9a-P**	9	7	7.5	8	6	6.5	46.05 ± 8.22 a
**9a-C**	9.5	9	10	8.5	8	9	32.89 ± 3.95 b
**TC-P**	9.5	9.5	10	8.5	8.5	9	31.58 ± 2.28 b
**TC-C**	9.5	13	12	8.5	12	11	17.11 ± 5.58 c
**CK**	15	14.5	11.5	14	13.5	10.5	

^a^ -P and -C represent the protective efficiency and curative efficiency, respectively. ^b^ Statistical analysis was conducted by ANOVA under the condition of equal variances assumed (*p* > 0.05) and equal variances not assumed (*p* < 0.05). Different lowercase letters indicate the control efficiencies with significant differences among different treatment groups at *p* < 0.05.

## Data Availability

Not applicable.

## Supplementary Materials

The following supporting information can be downloaded at: https://www.mdpi.com/article/10.3390/molecules28062442/s1. Figures S1–S56 show the ^1^H NMR, ^13^C NMR, ^19^F NMR and HRMS spectra of target compounds **7a**~**7f**, **8a**~**8d** and **9a**~**9d**. Figures S57–S58 and Table S1 show the HPLC spectra of target compounds **7c** and **9a**, respectively. Table S2 shows the results of ADME properties’ prediction of compounds **7c** and **9a**. Table S3 shows the toxic regression equation and correlation coefficient (R^2^) of active compounds against *Xoo*, *Xac* and *Psa*.

## Sample Availability

Samples of the compounds are available from the authors.

## References

[1] Jin Z. (2011). Muscarine, imidazole, oxazole, and thiazole alkaloids. Nat. Prod. Rep..

[2] Tolomeu H.V., Fraga C.A.M. (2023). Naturally occurring and synthetic imidazoles: Their chemistry and their biological activities. Molecules.

[3] Zhang L., Peng X.M., Damu G.L.V., Geng R.X., Zhou C.H. (2014). Comprehensive review in current developments of imidazole-based medicinal chemistry. Med. Res. Rev..

[4] Slassi S., Aarjane M., Amine A. (2023). Novel triazole derivatives possessing imidazole: Synthesis, spectroscopic characterization (FT-IR, NMR, UV-Vis), DFT studies and antibacterial *in vitro* evaluation. J. Mol. Struc..

[5] Liu H.B., Lauro G., O’Connor R.D., Lohith K., Kelly M., Colin P., Bifulco G., Bewley C.A. (2017). Tulongicin, an antibacterial tri-indole alkaloid from a deep-water *Topsentia* sp. sponge. J. Nat. Prod..

[6] Fan L., Wei Y., Chen Y., Jiang S., Xu F., Zhang C., Wang H., Shao X. (2023). Epinecidin-1, a marine antifungal peptide, inhibits *Botrytis cinerea* and delays gray mold in postharvest peaches. Food Chem..

[7] Kovalishyn V., Zyabrev V., Kachaeva M., Ziabrev K., Keith K., Harden E., Hartline C., James S.H., Brovarets V. (2021). Design of new imidazole derivatives with anti-HCMV activity: QSAR modeling, synthesis and biological testing. J. Comput. Aided Mol. Des..

[8] Kong T.T., Zhang C.M., Liu Z.P. (2013). Recent developments of p38*α* MAP kinase inhibitors as anti-inflammatory agents based on the imidazole scaffolds. Curr. Med. Chem..

[9] Basaleh A.S., Alomari F.Y., Sharfalddin A.A., Al-Radadi N.S., Domyati D., Hussien M.A. (2022). Theoretical investigation by DFT and molecular docking of synthesized oxidovanadium (IV)-based imidazole drug complexes as promising anticancer agents. Molecules.

[10] Wei L., Li Q., Chen Y., Zhang J., Mi Y., Dong F., Lei C., Guo Z. (2019). Enhanced antioxidant and antifungal activity of chitosan derivatives bearing 6-O-imidazole-based quaternary ammonium salts. Carbohyd. Polym..

[11] Poyraz S., Döndas H.A., Sansano J.M., Belveren S., Yamali C., Ülger M., Döndas N.Y., Saglık B.N., Pask C.M. (2023). *N*-Benzoylthiourea-pyrrolidine carboxylic acid derivatives bearing an imidazole moiety: Synthesis, characterization, crystal structure, *in vitro* ChEs inhibition, and antituberculosis, antibacterial, antifungal studies. J. Mol. Struct..

[12] Andreu Rico A., Sabater C., Castillo M.Á. (2016). Lethal and sub-lethal effects of five pesticides used in rice farming on the earthworm *Eisenia fetida*. Ecotox. Environ. Saf..

[13] Hao W., Zhong G., Hu M., Luo J., Weng Q., Rizwan-ul-Haq M. (2010). Control of citrus postharvest green and blue mold and sour rot by tea saponin combined with imazalil and prochloraz. Postharvest Biol. Technol..

[14] European Food Safety Authority (2014). Conclusion on the peer review of the pesticide risk assessment of the active substance thiabendazole. EFSA J..

[15] Wang Y., Fu L., Xu Z., Ji S., Zhuang P. (2022). Determination of Cyazofamid and Its Metabolite in Oily Agricultural Products with HPLC-MS/MS. J. Chromatogr. Sci..

[16] Song Y., Xu D., Lu H., He L., Chen L., Shao J., Xu C., Mu W., Liu F. (2016). Baseline sensitivity and efficacy of the sterol biosynthesis inhibitor triflumizole against *Botrytis cinerea*. Australas. Plant Pathol..

[17] Liu B., Zhu F., Huang Y., Wang Y., Yu F., Fan B., Yao J. (2010). Screening Rules for Leads of Fungicides, Herbicides, and Insecticides. J. Agric. Food Chem..

[18] Bourakadi K.E., Mekhzoum M.E.M., Qaiss A.E.K., Bouhfid R. (2020). Recent advances in the synthesis and applications of thiabendazole derivatives: A short review. Curr. Org. Chem..

[19] Cronly M., Behan P., Foley B., Malone E., Regan L. (2009). Rapid confirmatory method for the determination of 11 nitroimidazoles in egg using liquid chromatography tandem mass spectrometry. J. Chromatogr. A.

[20] Kumbhakar N.K., Sanyal P.K., Rawte D., Kumar D., Pal S. (2016). Influence of co-administration of parbendazole on the disposition kinetics and efficacy of albendazole in goats. Indian J. Small Rumin..

[21] Karuppiah C., Babulal S.M., Chen T.-W., Chen S.-M., Hsu L.-F., Al Farraj D.A., Ramaraj S.K., Elshikh M.S., Yang C.C. (2022). A novel ammonium zinc molybdate layered double hydroxide nanoflakes/vapor grown carbon fibers nanomaterials based electrocatalyst for the monitoring of dimetridazole drug in real samples. J. Environ. Chem. Eng..

[22] Yadav R., Bhullar M.S., Kaur S., Kaur T., Jhala A.J. (2017). Weed control in conventional soybean with pendimethalin followed by imazethapyr plus imazamox/quizalofop-p-ethyl. Can. J. Plant Sci..

[23] Lamberth C. (2021). Imidazole chemistry in crop protection. Heterocycles.

[24] Ningaiah S., Bhadraiah U.K., Doddaramappa S.D., Keshavamurthy S., Javarasetty C. (2014). Novel pyrazole integrated 1,3,4-oxadiazoles: Synthesis, characterization and antimicrobial evaluation. Bioorg. Med. Chem. Lett..

[25] Prakash O., Kumar M., Kumar R., Sharma C., Aneja K.R. (2010). Hypervalent iodine(III) mediated synthesis of novel unsymmetrical 2,5-disubstituted 1,3,4-oxadiazoles as antibacterial and antifungal agents. Eur. J. Med. Chem..

[26] Karad S.C., Purohit V.B., Avalani J.R., Sapariya N.H., Raval D.K. (2016). Design, synthesis and characterization of fluoro substituted novel pyrazole nucleus clubbed with 1,3,4-oxadiazole scaffolds and their biological applications. RSC Adv..

[27] Shamroukh A.H., Rashad A.E., Ali H.S., Awad S.M. (2014). Studies on the reactivity of amino-1-(6-phenyl-pyridazin-3-yl)-1H-pyrazole-4-carboxylic acid hydrazide towards some reagents for biological evaluation. J. Heterocycl. Chem..

[28] Rai N.P., Narayanaswamy V.K., Shashikanth S., Arunachalam P.N. (2009). Synthesis, characterization and antibacterial activity of 2-[1-(5-chloro-2-methoxy-phenyl)-5-methyl-1*H*-pyrazol-4-yl]-5-(substituted-phenyl)-[1,3,4]oxadiazoles. Eur. J. Med. Chem..

[29] Lee S.H., Seo H.J., Kim M.J., Kang S., Lee S.H., Ahn K., Lee M.W., Han H.K., Kim J., Lee J. (2009). Pentacycle derivatives as cannabinoid CB1 receptor ligands. Bioorg. Med. Chem. Lett..

[30] Abu-Zaied M.A., El-Telbani E.M., Elgemeie G.H., Nawwar G.A.M. (2011). Synthesis and in vitro anti-tumor activity of new oxadiazole thioglycosides. Eur. J. Med. Chem..

[31] El-Miligy M.M., Hazzaa A.A., El-Messmary H., Nassra R.A., El-Hawash S.A. (2017). New benzothiophene derivatives as dual COX-1/2 and 5-LOX inhibitors: Synthesis, biological evaluation and docking study. Future Med. Chem..

[32] Selvakumar B., Vaidyanathan S.P., Madhuri S., Elango K.P. (2017). Synthesis and antiviral activity of sulfonohydrazide and 1,3,4-oxadiazole derivatives of 6,6-dimethyl-9-oxo-4,5,6,7,8,9-hexahydropyrazolo[5,1-b] quinazoline. J. Chem. Res..

[33] Kumar M., Kumar V., Beniwal V. (2015). Synthesis of some pyrazolylaldehyde N-isonicotinoyl hydrazones and 2,5-disubstituted 1,3,4-oxadiazoles as DNA photocleaving agents. Med. Chem. Res..

[34] Zhang X.M., Yang Z., Xu H., Liu Y., Yang X., Sun T., Lu X., Shi F., Yang Q., Chen W. (2022). Synthesis, Antifungal Activity, and 3D-QASR of Novel 1,2,3,4-Tetrahydroquinoline Derivatives Containing a Pyrimidine Ether Scaffold as Chitin Synthase Inhibitors. J. Agric. Food Chem..

[35] Zeng F., Lu T., Wang J., Nie X., Xiong W., Yin Z., Peng D. (2022). Design, Synthesis and Bioactivity Evaluation of Coumarin–BMT Hybrids as New Acetylcholinesterase Inhibitors. Molecules.

[36] Wu H., Lu X., Xu J., Zhang X., Li Z., Yang X., Ling Y. (2022). Design, Synthesis and Fungicidal Activity of N-(thiophen-2-yl) Nicotinamide Derivatives. Molecules.

[37] Zhou L., Wang P.Y., Zhou J., Shao W.B., Fang H.S., Wu Z.B., Yang S. (2017). Antimicrobial activities of pyridinium-tailored pyrazoles bearing 1,3,4-oxadiazole scaffolds. J. Saudi Chem. Soc..

[38] Zhang T.T., Wang P.Y., Zhou J., Shao W.B., Fang H.S., Zhou X., Wu Z.B. (2017). Antibacterial and antifungal activities of 2-(substituted ether)-5-(1-phenyl-5-(trifluoromethyl)-1H-pyrazol-4-yl)-1,3,4-oxadiazole derivatives. J. Heterocycl. Chem..

[39] Zhao Y.L., Huang X., Liu L.W., Wang P.Y., Long Q.S., Tao Q.Q., Li Z., Yang S. (2019). Identification of racemic and chiral carbazole derivatives containing an isopropanolamine linker as prospective surrogates against plant pathogenic bacteria: *In vitro* and *in vivo* assays and quantitative proteomics. J. Agric. Food Chem..

[40] Wang M.W., Zhu H.H., Wang P.Y., Zeng D., Wu Y.Y., Liu L.W., Wu Z.B., Li Z., Yang S. (2019). Synthesis of thiazolium-labeled 1, 3, 4-oxadiazole thioethers as prospective antimicrobials: *In vitro* and *in vivo* bioactivity and mechanism of action. J. Agric. Food Chem..

[41] Wang F., Yang B.X., Zhang T.H., Tao Q.Q., Zhou X., Wang P.Y., Yang S. (2022). Novel 1, 3, 4-Oxadiazole Thioether and Sulfone Derivatives Bearing a Flexible *N*-Heterocyclic Moiety: Synthesis, Characterization, and Anti-microorganism Activity. Arab. J. Chem..

[42] Song Y.L., Liu H.W., Yang Y.H., He J.J., Yang B.X., Yang L.L., Zhou X., Liu L.W., Wang P.Y., Yang S. (2022). Novel 18β-glycyrrhetinic acid amide derivatives show dual-acting capabilities for control of plant bacterial diseases through ROS-mediated antibacterial efficiency and activation of plant defense responses. J. Integr. Agric..

[43] Zhu X.F., Xu Y., Peng D., Zhang Y., Huang T.T., Wang J.X., Zhou M.G. (2013). Detection and characterization of bismerthiazol-resistance of *Xanthomonas oryzae* pv. Oryzae. Crop. Prot..

[44] Feng C., Zhang C., Kong F., Wang J. (2014). Synthesis of thiodiazole copper microcapsules and release behavior of inhibiting R. solanacearum. RSC Adv..

[45] Wang P.Y., Wang M.W., Zeng D., Xiang M., Rao J.R., Liu Q.Q., Liu L.W., Wu Z.B., Li Z., Song B.A. (2019). Rational Optimization and Action Mechanism of Novel Imidazole (or Imidazolium)-Labeled 1,3,4-Oxadiazole Thioethers as Promising Anti-bacterial Agents against Plant Bacterial Diseases. J. Agric. Food Chem..

[46] Xiong G., Wu Z., Yi J., Fu L., Yang Z., Hsieh C., Yin M., Zeng X., Wu C., Lu A. (2021). ADMETlab 2.0: An integrated online platform for accurate and comprehensive predictions of ADMET properties. Nucleic Acids Res..

[47] Liu H.W., Ji Q.T., Ren G.G., Wang F., Su F., Wang P.Y., Zhou X., Wu Z.B., Li Z., Yang S. (2020). Antibacterial functions and proposed modes of action of novel 1,2,3,4-tetrahydro-*β*-carboline derivatives that Possess an attractive 1,3-diaminopropan-2-ol pattern against rice bacterial blight, kiwifruit bacterial canker, and citrus bacterial canker. J. Agric. Food Chem..

[48] Huang X., Liu H.W., Long Z.Q., Li Z.X., Zhu J.J., Wang P.Y., Qi P.Y., Liu L.W., Yang S. (2021). Rational optimization of 1,2,3-triazole-tailored carbazoles as prospective antibacterial alternatives with significant *in vivo* control efficiency and unique mode of action. J. Agric. Food Chem..

[49] Wang F., Liu H.W., Zhang L., Liu S.T., Zhang J.R., Zhou X., Wang P.Y., Yang S. (2022). Discovery of novel rost-4-ene derivatives as potential plant activators for preventing phytopathogenic bacterial infection: Design, synthesis and biological studies. Pest Manag. Sci..

[50] Dong Z., Xing S., Liu J., Tang X., Ruan L., Sun M., Tong Y., Peng D. (2018). Isolation and characterization of a novel phage Xoo-sp2 that infects *Xanthomonas oryzae* pv. oryzae. J. Gen. Virol..

[51] Faerber N., Reitler J., Kamenac A., Westerhausen C. (2022). Shear stress induced lipid order and permeability changes of giant unilamellar vesicles. Biochim. Biophys. Acta BBA Gen. Subj..

[52] Hammoudi Halat D., Younes S., Mourad N., Rahal M. (2022). Allylamines, Benzylamines, and Fungal Cell Permeability: A Review of Mechanistic Effects and Usefulness against Fungal Pathogens. Membranes-Basel..

[53] Bor B., Cen L., Agnello M., Shi W., He X. (2016). Morphological and physiological changes induced by contact-dependent interaction between *Candida albicans* and *Fusobacterium nucleatum*. Sci. Rep..

[54] Khan N.A., Rashid F., Jadoon M.S.K., Jalil S., Khan Z.A., Orfali R., Perveen S., Al-Taweel A., Iqbal J., Shahzad S.A. (2022). Design, Synthesis, and Biological Evaluation of Novel Dihydropyridine and Pyridine Analogs as Potent Human Tissue Nonspecific Alkaline Phosphatase Inhibitors with Anticancer Activity: ROS and DNA Damage-Induced Apoptosis. Molecules.

[55] Li J.L., Liu X.Y., Xie J.T., Di Y.L., Zhu F.X. (2014). A Comparison of Different Estimation Methods for Fungicide EC_50_ and EC_95_ Values. J. Phytopathol..

[56] Duncan D.B. (1955). Multiple range and multiple F tests. Biometrics.

