# Supplementary material for: Design, Synthesis and Bioactivity Evaluation of Novel 2-(pyrazol-4-yl)-1,3,4-oxadiazoles Containing an Imidazole Fragment as Antibacterial Agents
Source: Molecules. 2023 Mar 7;28(6):2442. doi: 10.3390/molecules28062442 (PMC10058659; doi:10.3390/molecules28062442)
Supplement: Supplementary file 1 [file molecules-28-02442-s001.zip › molecules-2219392-supplementary.pdf]

Supplementary Materials

Design, Synthesis and Bioactivity Evaluation of Novel 2-(pyrazol-4-yl)-1,3,4-oxadiazoles Decorated with Imidazole Fragment as Antibacterial Agents

Content

1. General synthetic protocols for target compounds **7a~7f**, **8a~8d** and **9a~9d**..............2
2. ^{1}H NMR, ^{13}C NMR, ^{19}F NMR and HRMS spectra of target compounds **7a~7f**, **8a~8d** and **9a~9d** (**Figure S1-S56**)..12
3. *In Vitro* Antibacterial Bioassay of target compounds against *Xoo*, *Xac*, and *Psa*.....40
4. *In Vivo* Bioassay of Compound **7c** against Rice Bacterial Leaf Blight.......................41
5. Morphological observation of *Xoo* cells by scanning electron microscopy (SEM) ...43
6. Determination of purity of active compounds **7c** and **9a**……………………….……43
7. The ADME properties prediction of compounds **7c** and **9a**……………………...…45
8. The toxic regression equation and correlation coefficient (R^2) of active compounds against *Xoo*, *Xac* and *Psa*……………………………………………………….45

1. The synthesis for title compounds target compounds **7a~7f**, **8a~8d** and **9a~9d**

1.1 The synthetic protocols for intermediates **3**

Firstly, the starting material ethyl 4,4,4-trifluoroacetoacetate **1** (186.03 mmol), triethyl orthoformate (372.05 mmol) and glacial acetic acid (558.08 mmol) were successively added into a 250 mL reaction flask, then reacted at 120 °C for 12 h. After that, the reaction mixture was cooled to room temperature. Subsequently, the solvent and excess triethyl orthoformate were distilled under pressure to obtain crude intermediate **2**. Secondly, the 20.73 g of crude intermediate **2** was dissolved by absolute

ethyl alcohol (150 mL), then phenylhydrazine (90.62mmol) was slowly added. The mixture was reflux for 5 h. After that, the solvent was distilled under pressure to obtain the crude product and further purified by flash column chromatography on a silica gel using ethyl acetate and petroleum ether (1: 20) as the eluant to afford the desired products **3B** as a yellow oil, yield 83.2%. ^{1}H NMR (500 MHz, DMSO-d_6) δ 8.31 (s, 1H, pyrazole-H), 7.65-7.54 (m, 5H, benzene-H), 4.32(q, 2H, J = 7.2 Hz, CH_2), 1.31(t, 3H, J = 7.2 Hz, CH_3).

1.2 The synthetic protocols for intermediates **4B**

The intermediate **3B** (30 mmol) and hydrazine hydrate (60 mmol) were successively added into a 250 mL reaction flask, then the mixture was reacted under reflux for 5 h. After that, the reaction mixture was cooled to room temperature, and the precipitated solid was filtered via vacuum and washed by water (3×5 mL) to obtain the intermediates **4B**. A white solid, yield 92.8%, m.p. 146~148 ºC; ^{1}H NMR (500 MHz, DMSO-d_6) δ 9.73 (s, 1H, NH), 8.05 (s, 1H, pyrazole H), 7.73-7.41 (m, 5H, benzene H), 4.50 (s, 2H, NH2); ^{13}C NMR (125 MHz, DMSO-d_6) δ 160.3, 139.9, 139.4, 130.5, 129.9, 126.5, 120.3, 119.8 (q, $^1J_{C\text{-}F}$ = 270.9 Hz, CF_3).

1.3 General synthesis protocols for intermediates **5B**

Intermediate **4B** (14.80 mmol) was dissolved in a mixture of THF (20.0 mL), and *N*,*N*'-carbonyldimidazole (17.76 mmol), and then triethylamine (6.0 mL) was added. The mixture was stirred at room temperature for 6 hours. After that, THF was removed under reduce pressure. The crude residue was further purified by flash column chromatography on a silica gel and using CH_2Cl_2 and CH_3OH (100: 1) as the eluant to

afford the desired products **5B** as white soild, yield 90.8%, m.p. 81~83 ºC ; ^1H NMR (500 MHz, DMSO-d_6) δ 8.33 (s, 1H, pyrazole-H), 7.65-7.48 (m, 5H, benzene-H); ^{13}C NMR (126 MHz, DMSO-d_6) δ 177.9, 154.2, 141.1, 138.8, 130.9, 129.9, 126.6, 119.3 (q, $^1J_{C\text{-}F}$ = 270.9 Hz, CF_3), 108.0.

For the synthesis protocols of intermediates **3A**, **4A** and **5A** were consistent to intermediates **3B**, **4B** and **5B**, respectively. And the NMR date information for the key intermediates **5A** as following: ^1H NMR (500 MHz, $CDCl_3$) δ 7.88 (s, 1H, pyrazole-H), 4.10 (s, 3H, CH_3); ^{13}C NMR (126 MHz, $CDCl_3$) δ 154.6, 148.8, 139.1, 130.0 (q, $^2J_{C\text{-}F}$ = 40.7 Hz, $\underline{C}CF_3$), 119.5 (q, ^1J = 269.1 Hz, CF_3), 108.1, 39.9.

1.4 The synthesis protocol for intermediate **6**

Intermediate **5B** (25.91 mmol) was added into 60 mL ethanol containing KOH (25.91 mmol), then CS_2 (51.82 mmol) was added dropwise. The mixture was stirred at room temperature for 6 hours. After that, the solvent was removed under reduced pressure, and the residue was re-dissolved in 100 mL ethanol containing KOH (25.91 mmol). Later, the mixture was heated to reflux for 10 h. Finally, the solvent was removed under reduced pressure, and re-dissolved in 40 mL water, then acidified with conc. HCl solution. The precipitates were filtered, washed with water, and dried under infrared light to give intermediate **6** as a white soild, yield 76.0%, m.p. 169~170 ºC; ^1H NMR (500 MHz, DMSO-d_6) δ 8.43 (s, 1H, pyrazole-H), 7.72-7.46 (m, 6H, SH & benzene-H); ^{13}C NMR (126 MHz, DMSO-d_6) δ 177.9, 154.2, 141.1, 138.9, 130.9, 129.9, 126.6, 119.4 (q, $J_{C\text{-}F}$ = 270.9 Hz, CF_3), 108.04.

1.5 General synthetic protocols for target compounds **7a~7f**, **8a~8d** and **9a~9d**

As depicted in **Scheme 1**, the intermediate **2**-**6** were obtained according to our previously reported methods. Further, intermediate compound 5-(1-phenyl-5-(trifluoromethyl)-1*H*-pyrazol-4-yl)-1,3,4-oxadiazol-2-ol (**5A**) [5-(1-methyl-3-(trifluoromethyl)-1*H*-pyrazol-4-yl)-1,3,4-oxadiazol-2-ol (**5B**) or 5-(1-phenyl-5-(trifluoromethyl)-1*H*-pyrazol-4-yl)-1,3,4-oxadiazole-2-thiol (**6**)] (1.01 mM), NaOH (1.50 mM) and DMF (10.0 mL) were stirred at room temperature for 20 min. Then, the corresponding dibromo alkane (1.41 mM) was slowly dropped into the reaction system and continually reacted at room temperature for another 2 h. After that, the reaction was diluted with ethyl acetate, washed by saturated ammonium chloride solution, dried using anhydrous sodium sulfate and evaporated under vacuum. Subsequently, the crude product was added into a mixture of imidazole (1.00 mM), NaH (1.20 mM) and DMF (2 mL) under ice bath condition, and reacted at room temperature for 4 h. After that, the reaction was diluted with ethyl acetate, washed by saturated ammonium chloride solution, dried by anhydrous sodium sulfate, and evaporated under vacuum. Finally, the target compounds were purified by column chromatography on a silica gel using CH_2Cl_2 and CH_3OH (30:1) as the eluant to afford the desired products **7a**~**7f**, **8a**~**8d** and **9a**~**9d**, respectively.

*2-(4-(1H-imidazol-1-yl)butoxy)-5-(1-phenyl-5-(trifluoromethyl)-1H-pyrazol-4-yl)-1,3,4-oxadiazole (7**a**)*

A yellow liquid, yield 21.0%. ^{1}H NMR (500 MHz, $CDCl_3$) δ 8.06 (s, 1H, Pyrazole-H), 7.57 – 7.37 (m, 6H, imidazole-H, phenyl-H), 7.06 (s, 1H, imidazole-H), 6.91 (s, 1H, imidazole-H), 4.00 (t, J = 6.9 Hz, 2H, CH_2), 3.82 (t, J = 6.6 Hz, 2H, CH_2), 1.93 – 1.84

(m, 2H, CH_2), 1.80 (tt, *J* = 13.4, 6.5 Hz, 2H, CH_2); ^{13}C NMR (101 MHz, $CDCl_3$) δ 153.2, 146.9, 140.0, 138.9, 137.2, 130.5 (q, $^2J_{C\text{-}F}$ = 40.5 Hz, <u>C</u>CF_3), 130.3, 129.8, 129.4, 125.9, 119.1 (q, $^1J_{C\text{-}F}$ = 271.7 Hz, CF_3), 118.8, 109.0, 46.3, 45.3, 28.0, 25.4; ^{19}F NMR (471 MHz, $CDCl_3$) δ -55.7 (CF_3); HRMS (ESI) $[M+H]^+$ calcd for $C_{19}H_{18}O_2N_6F_3$: 419.1438, found: 419.1430.

*2-((5-(1H-imidazol-1-yl)pentyl)oxy)-5-(1-phenyl-5-(trifluoromethyl)-1H-pyrazol-4-yl)-1,3,4-oxadiazole (7**b**)*

A yellow liquid, yield 14.0%; ^1H NMR (500 MHz, $CDCl_3$) δ 8.07 (s, 1H, Pyrazole-H), 7.57 – 7.39 (m, 6H, imidazole-H, phenyl-H), 7.05 (s, 1H, imidazole-H), 6.91 (s, 1H, imidazole-H), 3.94 (t, *J* = 7.1 Hz, 2H, CH_2), 3.79 (t, *J* = 6.9 Hz, 2H, CH_2), 1.89 – 1.76 (m, 4H, $2CH_2$), 1.43 – 1.33 (m, 2H, CH_2); ^{13}C NMR (126 MHz, $CDCl_3$) δ 153.2, 146.7, 140.0, 138.9, 137.2, 130.4 (q, $^2J_{C\text{-}F}$ = 40.5 Hz, <u>C</u>CF_3),130.3 , 129.4, 125.9, 120.2, 119.0 (q, $^1J_{C\text{-}F}$ = 264.1 Hz, CF_3), 109.0, 46.9, 45.7, 30.6, 27.7, 23.5; ^{19}F NMR (471 MHz, $CDCl_3$) δ -55.7 (CF_3); HRMS (ESI) $[M+H]^+$ calcd for $C_{20}H_{20}O_2N_6F_3$: 433.1594, found: 433.1584.

*2-((6-(1H-imidazol-1-yl)hexyl)oxy)-5-(1-phenyl-5-(trifluoromethyl)-1H-pyrazol-4-yl)-1,3,4-oxadiazole (7**c**)*

A yellow liquid, yield 23.3%; ^1H NMR (500 MHz, $CDCl_3$) δ 8.06 (s, 1H, Pyrazole-H), 7.58 – 7.48 (m, 3H, imidazole-H, phenyl-H), 7.46 – 7.39 (m, 3H, imidazole-H, phenyl-H), 7.03 (s, 1H, imidazole-H), 6.88 (s, 1H, imidazole-H), 3.91 (t, *J* = 7.1 Hz, 2H, CH_2), 3.77 (t, *J* = 7.0 Hz, 2H, CH_2), 1.78 (dt, *J* = 14.1, 7.0 Hz, 4H, $2CH_2$), 1.47 – 1.29 (m, 4H, $2CH_2$); ^{13}C NMR (101 MHz, $CDCl_3$) δ 153.3, 146.6, 134.0, 138.9, 137.1,

130.7 (q, $^{2}J_{C-F}$ = 39.5 Hz, <u>C</u>CF3), 130.2, 129.5, 129.4, 125.9, 119.1 (q, $^{1}J_{C-F}$ = 271.2 Hz, CF_3), 118.8, 109.1, 46.9, 45.9, 31.0, 28.0, 26.1, 25.9; ^{19}F NMR (471 MHz, $CDCl_3$) δ -55.7 (CF_3); HRMS (ESI) $[M+H]^+$ calcd for $C_{21}H_{22}O_2N_6F_3$: 447.1751, found: 447.1742.

*2-((8-(1H-imidazol-1-yl)octyl)oxy)-5-(1-phenyl-5-(trifluoromethyl)-1H-pyrazol-4-yl)-1,3,4-oxadiazole (7**d**)*

A yellow liquid, yield 9.1%; ^{1}H NMR (500 MHz, $CDCl_3$) δ 8.06 (s, 1H, Pyrazole-H), 7.58 – 7.47 (m, 3H, imidazole-H, phenyl-H), 7.45 (dd, J = 6.7, 2.8 Hz, 3H, imidazole-H, phenyl-H), 7.03 (s, 1H, imidazole-H), 6.89 (s, 1H, imidazole-H), 3.91 (t, J = 7.1 Hz, 2H, CH_2), 3.78 (t, J = 7.1 Hz, 2H, CH_2), 1.77 (tt, J = 14.4, 7.1 Hz, 4H, $2CH_2$), 1.39 – 1.27 (m, 8H, $4CH_2$); ^{13}C NMR (101 MHz, $CDCl_3$) δ 153.3, 146.6, 140.0, 138.9, 137.2, 130.9 (q, $^{2}J_{C-F}$ = 44.4 Hz, <u>C</u>CF3), 130.2, 129.5, 129.4, 125.9, 119.1 (q, $^{1}J_{C-F}$ = 272.1 Hz, CF_3), 118.9, 109.2, 47.1, 46.1, 31.1, 29.0, 28.9, 28.2, 26.5, 26.3; ^{19}F NMR (471 MHz, $CDCl_3$) δ -55.7 (CF_3); HRMS (ESI) $[M+H]^+$ calcd for $C_{23}H_{26}O_2N_6F_3$: 475.2034, found: 475.2054.

*2-((10-(1H-imidazol-1-yl)decyl)oxy)-5-(1-phenyl-5-(trifluoromethyl)-1H-pyrazol-4-yl)-1,3,4-oxadiazole (7**e**)*

A yellow liquid, yield 10.0%; ^{1}H NMR (500 MHz, $CDCl_3$) δ 8.08 (s, 1H, Pyrazole-H), 7.55 – 7.51 (m, 3H, imidazole-H, phenyl-H), 7.48 – 7.43 (m, 3H, imidazole-H, phenyl-H), 7.05 (s, 1H, imidazole-H), 6.90 (s, 1H, imidazole-H), 3.92 (t, J = 7.2 Hz, 2H, CH_2), 3.79 (t, J = 7.1 Hz, 2H, CH_2), 1.80 – 1.76 (m, 4H, $2CH_2$), 1.38 – 1.25 (m, 12H, $6CH_2$); ^{13}C NMR (101 MHz, $CDCl_3$) δ 153.3, 146.6, 140.0, 139.0, 137.2, 130.8 (q, $^{2}J_{C-F}$ = 40.6 Hz, <u>C</u>CF3), 130.3, 129.5, 125.9, 119.0 (q, $^{1}J_{C-F}$ = 272.2 Hz, CF_3), 118.1,

109.3, 47.2, 46.2, 31.2, 29.8, 29.4, 29.1, 29.1, 28.3, 26.7, 26.4; ^{19}F NMR (471 MHz, $CDCl_3$) δ -55.7 (CF_3); HRMS (ESI) $[M+H]^+$ calcd for $C_{25}H_{30}O_2N_6F_3$: 503.2377, found: 503.2367.

*2-((12-(1H-imidazol-1-yl)dodecyl)oxy)-5-(1-phenyl-5-(trifluoromethyl)-1H-pyrazol-4-yl)-1,3,4-oxadiazole (**7f**)*

A yellow liquid, yield 34.6%; ^{1}H NMR (400 MHz, $CDCl_3$) δ 8.07 (s, 1H, Pyrazole-H), 7.56 – 7.42 (m, 6H, imidazole-H, phenyl-H), 7.04 (s, 1H, imidazole-H), 6.89 (s, 1H, imidazole-H), 3.90 (t, J = 7.2 Hz, 2H, CH_2), 3.79 (t, J = 7.1 Hz, 2H, CH_2), 1.78 (td, J = 14.3, 7.2 Hz, 4H, $2CH_2$), 1.37 – 1.22 (m, 16H, $8CH_2$); ^{13}C NMR (101 MHz, $CDCl_3$) δ 153.3, 146.5, 140.0, 139.0, 137.2, 130.4 (q, $^2J_{C\text{-}F}$ = 40.4 Hz, $\underline{C}CF_3$), 130.2, 129.4, 125.3, 119.1 (q, $^1J_{C\text{-}F}$ = 272.7 Hz, CF_3), 118.9, 109.3, 47.1, 46.2, 31.1, 29.5, 29.2, 29.1, 28.2, 26.6, 26.4; ^{19}F NMR (471 MHz, $CDCl_3$) δ -55.8 (CF_3); HRMS (ESI) $[M+H]^+$ calcd for $C_{27}H_{34}O_2N_6F_3$: 531.2690, found: 531.2678.

*2-((6-(1H-imidazol-1-yl)hexyl)oxy)-5-(1-methyl-3-(trifluoromethyl)-1H-pyrazol-4-yl)-1,3,4-oxadiazole (**8a**)*

A yellow liquid, yield 10.5%; ^{1}H NMR (500 MHz, $CDCl_3$) δ 7.86 (s, 1H, Pyrazole-H), 7.47 (s, 1H, imidazole-H), 7.06 (s, 1H, imidazole-H), 6.91 (s, 1H, imidazole-H), 4.09 (dd, J = 2.6, 1.2 Hz, 3H, CH_3), 3.93 (t, J = 7.1 Hz, 2H, CH_2), 3.76 (t, J = 7.0 Hz, 2H, CH_2), 1.86–1.68 (m, 4H, $2CH_2$), 1.46–1.29 (m, 4H, $2CH_2$); ^{13}C NMR (101 MHz, $CDCl_3$) δ 153.3, 146.7, 146.7, 138.8, 129.7 (q, $^2J_{C\text{-}F}$ = 40.8 Hz, $\underline{C}CF_3$), 120.8, 119.5 (q,$^1J_{C\text{-}F}$ = 270.6 Hz, CF_3), 118.1, 108.2, 47.0, 45.8, 39.9 (q, $^5J_{C\text{-}F}$ = 2.7 Hz, NCH_3), 31.0, 28.0, 26.1, 25.9; ^{19}F NMR (471 MHz, $CDCl_3$) δ -57.8 (CF_3); HRMS (ESI) $[M+H]^+$

calcd for $C_{16}H_{20}O_2N_6F_3$: 385.1594, found: 385.1584.

*2-((8-(1H-imidazol-1-yl)octyl)oxy)-5-(1-methyl-3-(trifluoromethyl)-1H-pyrazol-4-yl)-1,3,4-oxadiazole (**8b**)*

A yellow liquid, yield 9.2%; ^{1}H NMR (500 MHz, $CDCl_3$) δ 7.83 (s, 1H, Pyrazole-H), 7.43 (s, 1H, imidazole-H), 7.02 (s, 1H, imidazole-H), 6.87 (s, 1H, imidazole-H), 4.07 (d, J = 1.3 Hz, 3H, CH_3), 3.89 (t, J = 7.1 Hz, 2H, CH_2), 3.73 (t, J = 7.1 Hz, 2H, CH_2), 1.65-1.80 (m, 4H, 2CH_2), 1.31 – 1.23 (m, 8H, 4CH_2); ^{13}C NMR (101 MHz, $CDCl_3$) δ 153.3, 146.6, 138.8, 137.1, 130.1 (q, $^2J_{C-F}$ = 37.4 Hz, $\underline{C}CF_3$), 129.4, 119.4 (q,$^1J_{C-F}$ = 276.1 Hz, CF_3), 118.8, 108.3, 47.1, 46.0, 39.9 (q, $^5J_{C-F}$ = 2.7 Hz, NCH_3), 31.1, 28.9, 28.9, 28.1, 26.5, 26.2; ^{19}F NMR (471 MHz, $CDCl_3$) δ -58.0 (CF_3); HRMS (ESI) $[M+H]^+$ calcd for $C_{18}H_{24}O_2N_6F_3$: 413.1907, found:413.1897.

*2-((10-(1H-imidazol-1-yl)decyl)oxy)-5-(1-methyl-3-(trifluoromethyl)-1H-pyrazol-4-yl)-1,3,4-oxadiazole (**8c**)*

A yellow liquid, yield 27.3%; ^{1}H NMR (500 MHz, $CDCl_3$) δ 7.82 (s, 1H, Pyrazole-H), 7.43 (s, 1H, imidazole-H), 7.01 (s, 1H, imidazole-H), 6.87 (s, 1H, imidazole-H), 4.15 – 3.98 (s, 3H, CH_3), 3.89 (t, J = 7.0 Hz, 2H, CH_2), 3.73 (t, J = 7.1 Hz, 2H, CH_2), 1.66 (d, J = 4.3 Hz, 4H, 2CH_2), 1.27 (d, J = 17.3 Hz, 12H, 6CH_2); ^{13}C NMR (101 MHz, $CDCl_3$) δ 153.3, 146.5, 138.8, 137.1, 129.7 (q, $^2J_{C-F}$ = 38.4 Hz, $\underline{C}CF_3$), 129.3, 119.4 (q,$^1J_{C-F}$ = 269.3 Hz, CF_3), 118.8, 108.2, 47.1, 46.1, 39.8 (q, $^5J_{C-F}$ = 2.7 Hz, NCH_3), 31.1, 29.3, 29.0, 28.9, 28.1, 26.6, 26.3; ^{19}F NMR (471 MHz, $CDCl_3$) δ -58.0 (CF_3); HRMS (ESI) $[M+H]^+$ calcd for $C_{20}H_{28}O_2N_6F_3$: 441.2220, found: 441.2211.

2-((12-(1H-imidazol-1-yl)dodecyl)oxy)-5-(1-methyl-3-(trifluoromethyl)-1H-

pyrazol-4-yl)-1,3,4-oxadiazole ***(8d)***

A yellow liquid, yield 14.3%; ^{1}H NMR (500 MHz, $CDCl_3$) δ 7.86 (s, 1H, Pyrazole-H), 7.47 (s, 1H, imidazole-H), 7.05 (s, 1H, imidazole-H), 6.91 (s, 1H, imidazole-H), 4.09 (s, 3H, CH_3), 3.94 (t, J = 7.2 Hz, 2H, CH_2), 3.76 (t, J = 7.2 Hz, 2H, CH_2), 1.79 (dd, J = 17.1, 12.3 Hz, 4H, $2CH_2$), 1.30 (d, J = 17.9 Hz, 16H, $8CH_2$); ^{13}C NMR (101 MHz, $CDCl_3$) δ 153.2, 146.5, 138.7, 137.1, 129.6 (q, $^2J_{C\text{-}F}$ = 40.4 Hz, $\underline{C}CF_3$), 129.2, 119.3 (q,$^1J_{C\text{-}F}$ = 272.7 Hz, CF_3), 118.8, 108.2, 47.1, 46.0, 39.8 (q, $^5J_{C\text{-}F}$ = 2.8 Hz, NCH_3), 31.1, 29.4, 29.4, 29.4, 29.4, 29.0, 28.1, 26.5, 26.3; ^{19}F NMR (471 MHz, $CDCl_3$) δ -58.0 (CF_3); HRMS (ESI) $[M+H]^+$ calcd for $C_{22}H_{32}O_2N_6F_3$: 469.2533, found: 469.2526.

2-((6-(1H-imidazol-1-yl)hexyl)thio)-5-(1-phenyl-5-(trifluoromethyl)-1H-pyrazol-4-yl)-1,3,4-oxadiazole ***(9a)***

A yellow liquid, yield 40.7%; ^{1}H NMR (500 MHz, $CDCl_3$) δ 8.22 (s, 1H, Pyrazole-H), 7.70 (s, 1H, imidazole-H), 7.53 (ddd, J = 12.6, 5.5, 3.0 Hz, 5H, phenyl-H), 7.14 (s, 1H, imidazole-H), 6.95 (s, 1H, imidazole-H), 3.98 (t, J = 7.1 Hz, 2H, CH_2), 3.28 (t, J = 7.3 Hz, 2H, CH_2), 1.84 (dt, J = 20.0, 7.3 Hz, 4H, $2CH_2$), 1.52 (dd, J = 15.5, 7.5 Hz, 2H, CH_2), 1.38 (dd, J = 15.4, 8.1 Hz, 2H, CH_2); ^{13}C NMR (101 MHz, $CDCl_3$) δ 165.3, 158.6, 140.5, 138.8, 137.1, 130.4 (q, $^2J_{C\text{-}F}$ = 40.3 Hz, $\underline{C}CF_3$), 130.2, 129.3, 129.3, 128.6, 125.8, 119.1 (q, $^1J_{C\text{-}F}$ = 271.2 Hz, CF_3), 119.0, 108.5, 47.2, 32.3, 30.8, 29.1, 28.0, 26.0; ^{19}F NMR (471 MHz, $CDCl_3$) δ -55.8 (CF_3); HRMS (ESI) $[M+H]^+$ calcd for $C_{21}H_{22}ON_6F_3S$: 463.1522, found: 463.1510.

2-((8-(1H-imidazol-1-yl)octyl)thio)-5-(1-phenyl-5-(trifluoromethyl)-1H-pyrazol-4-yl)-1,3,4-oxadiazole ***(9b)***

A yellow liquid, yield 20.0%; ^{1}H NMR (500 MHz, $CDCl_3$) δ 8.20 (s, 1H, Pyrazole-H), 7.57 – 7.50 (m, 3H, imidazole-H, phenyl-H), 7.48 (dd, J = 6.5, 4.7 Hz, 3H, imidazole-H, phenyl-H), 7.05 (s, 1H, imidazole-H), 6.90 (s, 1H, imidazole-H), 3.92 (t, J = 7.1 Hz, 2H, CH_2), 3.34 – 3.16 (m, 2H, CH_2), 1.90 – 1.70 (m, 4H, $2CH_2$), 1.45 (dt, J = 14.1, 6.9 Hz, 2H, CH_2), 1.39 – 1.23 (m, 6H, $3CH_2$); ^{13}C NMR (101 MHz, $CDCl_3$) δ 165.5, 158.7, 140.6, 139.0, 137.2, 130.6 (q, $^2J_{C\text{-}F}$ = 40.5 Hz, <u>C</u>CF_3),130.2, 129.4, 126.0, 119.2 (q,$^1J_{C\text{-}F}$ = 272.0 Hz, CF_3), 118.9, 108.7, 47.1, 32.7, 31.1, 29.4, 29.0, 28.9, 28.5, 26.6; ^{19}F NMR (471 MHz, $CDCl_3$) δ -55.8 (CF_3); HRMS (ESI) $[M+H]^+$ calcd for $C_{23}H_{25}ON_6F_3S$: 491.1835, found: 491.1825.

*2-((10-(1H-imidazol-1-yl)decyl)thio)-5-(1-phenyl-5-(trifluoromethyl)-1H-pyrazol-4-yl)-1,3,4-oxadiazole (**9c**)*

A yellow liquid, yield 27.2%; ^{1}H NMR (400 MHz, $CDCl_3$) δ 8.20 (s, 1H, Pyrazole-H), 7.56–7.50 (m, 3H, imidazole-H, phenyl-H), 7.49–7.43 (m, 3H, imidazole-H, phenyl-H), 7.04 (s, 1H, imidazole-H), 6.89 (s, 1H, imidazole-H), 3.90 (t, J = 7.1 Hz, 2H, CH_2), 3.33–3.17 (m, 2H, CH_2), 1.80 (ddd, J = 20.6, 14.3, 7.1 Hz, 4H, $2CH_2$), 1.50–1.38 (m, 2H, CH_2), 1.28 (s, 10H, $5CH_2$); ^{13}C NMR (101 MHz, $CDCl_3$) δ 165.5, 158.6, 140.6, 138.9, 137.1, 130.5 (q, $^2J_{C\text{-}F}$ = 40.4 Hz, <u>C</u>CF_3), 130.2, 129.4, 129.3, 125.9, 119.2 (q, $^1J_{C\text{-}F}$ = 271.2 Hz, CF_3), 118.8, 108.7, 47.1, 32.7, 31.2, 29.4, 29.3, 29.1, 29.0, 28.6, 26.6; ^{19}F NMR (376 MHz, $CDCl_3$) δ -55.8 (CF_3); HRMS (ESI) $[M+H]^+$ calcd for $C_{25}H_{30}ON_6F_3S$: 519.2148, found: 519.2142.

*2-((12-(1H-imidazol-1-yl)dodecyl)thio)-5-(1-phenyl-5-(trifluoromethyl)-1H-pyrazol-4-yl)-1,3,4-oxadiazole (**9d**)*

A yellow liquid, yield 30.7%; ^1H NMR (400 MHz, $CDCl_3$) δ 8.20 (s, 1H, Pyrazole-H), 7.54 – 7.45 (m, 6H, imidazole-H, phenyl-H), 7.07 (s, 1H, imidazole-H), 6.90 (s, 1H, imidazole-H), 3.92 (t, J = 7.2 Hz, 2H, CH_2), 3.31 – 3.20 (m, 2H, CH_2), 1.87 – 1.75 (m, 4H, $2CH_2$), 1.50 – 1.39 (m, 2H, CH_2), 1.32 – 1.24 (m, 14H, $7CH_2$); ^{13}C NMR (101 MHz, $CDCl_3$) δ 165.6, 158.5, 140.5, 138.9, 137.1, 130.5 (q, $^2J_{C-F}$ = 40.6 Hz, $\underline{C}CF_3$), 130.1, 129.4, 129.3, 125.8, 119.1 (q, $^1J_{C-F}$ = 271.7 Hz, CF_3), 118.9, 108.6, 47.2, 32.7, 31.1, 29.5, 29.5, 29.4, 29.4, 29.3, 29.0, 29.0, 28.6, 26.5; ^{19}F NMR (376 MHz, $CDCl_3$) δ -55.8 (CF_3); HRMS (ESI) $[M+H]^+$ calcd for $C_{27}H_{34}ON_6F_3S$: 547.2461, found: 547.2452.

2. ^{1}H NMR, ^{13}C NMR, ^{19}F NMR and HRMS spectra of target compounds **7a~7f**, **8a~8d** and **9a~9d**

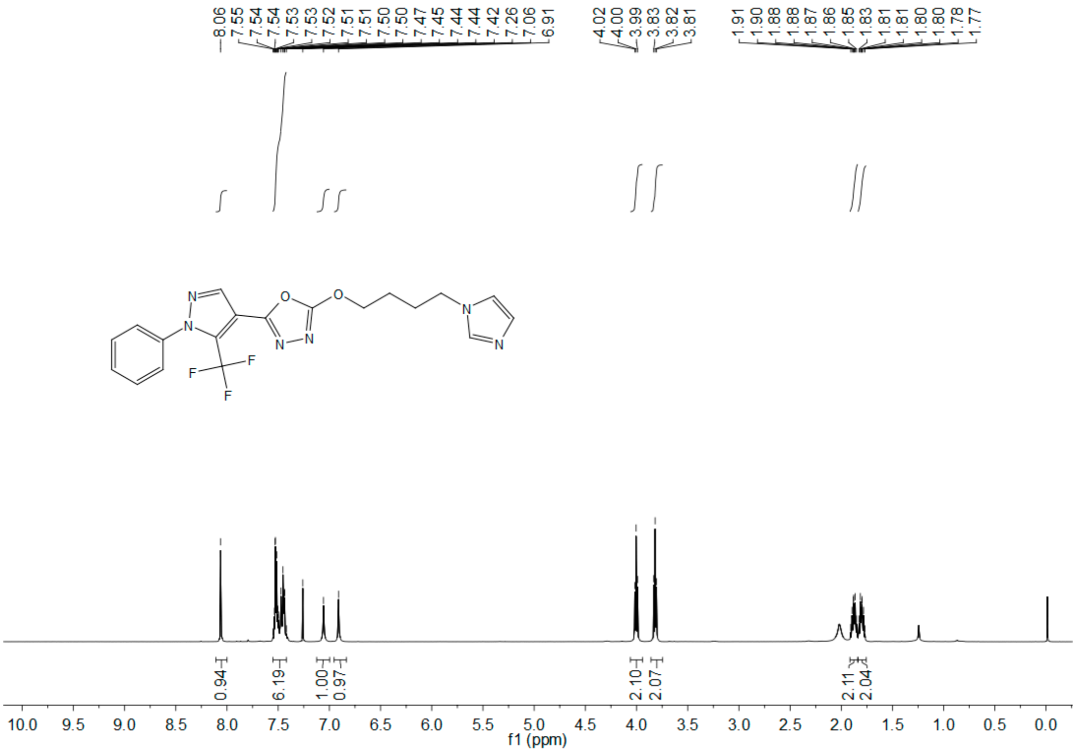

Figure S1. ^{1}H NMR spectrum of target compound **7a.**

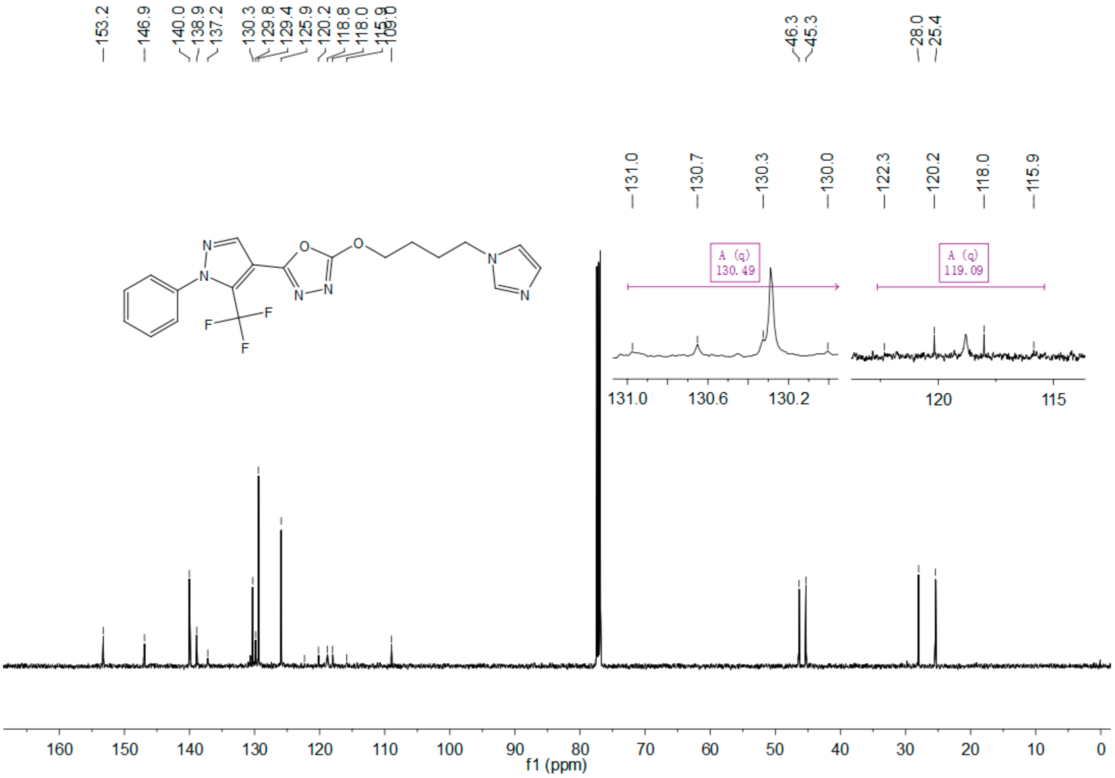

Figure S2. ^{13}C NMR spectrum of target compound **7a.**

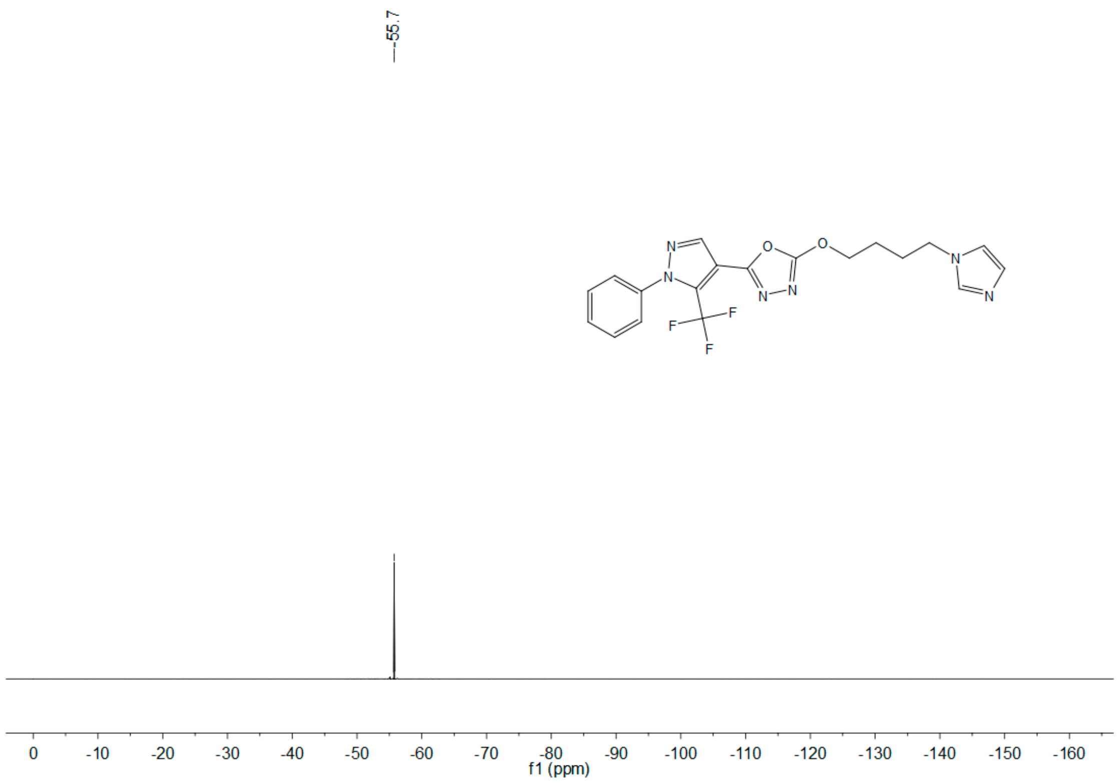

Figure S3. ^{19}F NMR spectrum of target compound **7a.**

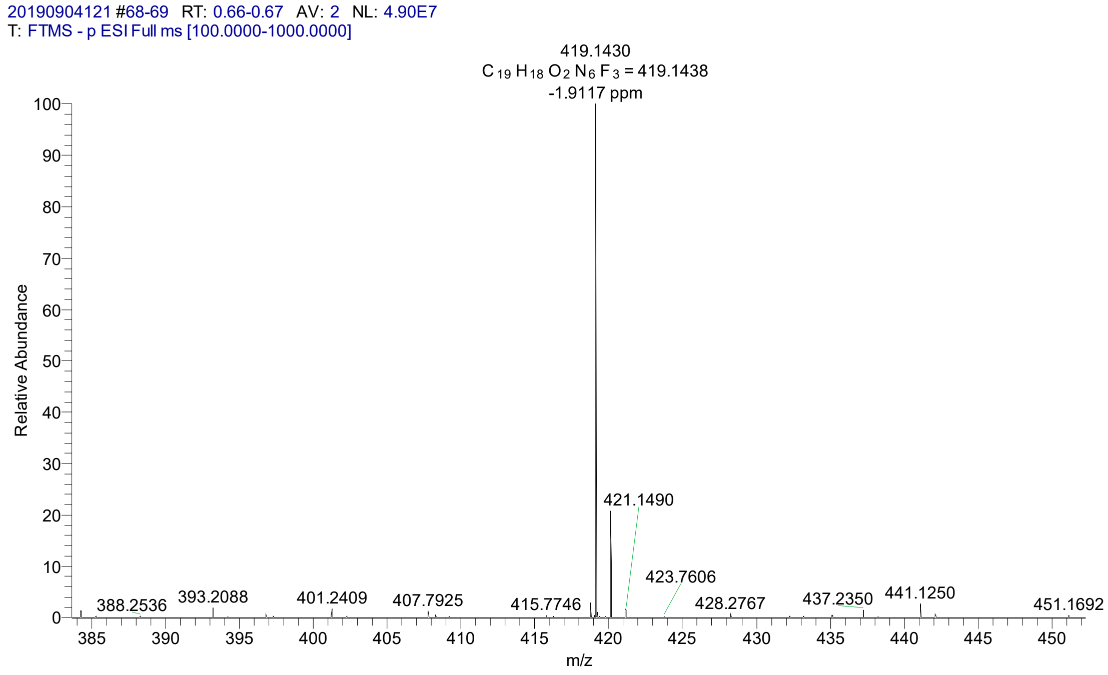

Figure S4. HRMS spectrum of target compound **7a.**

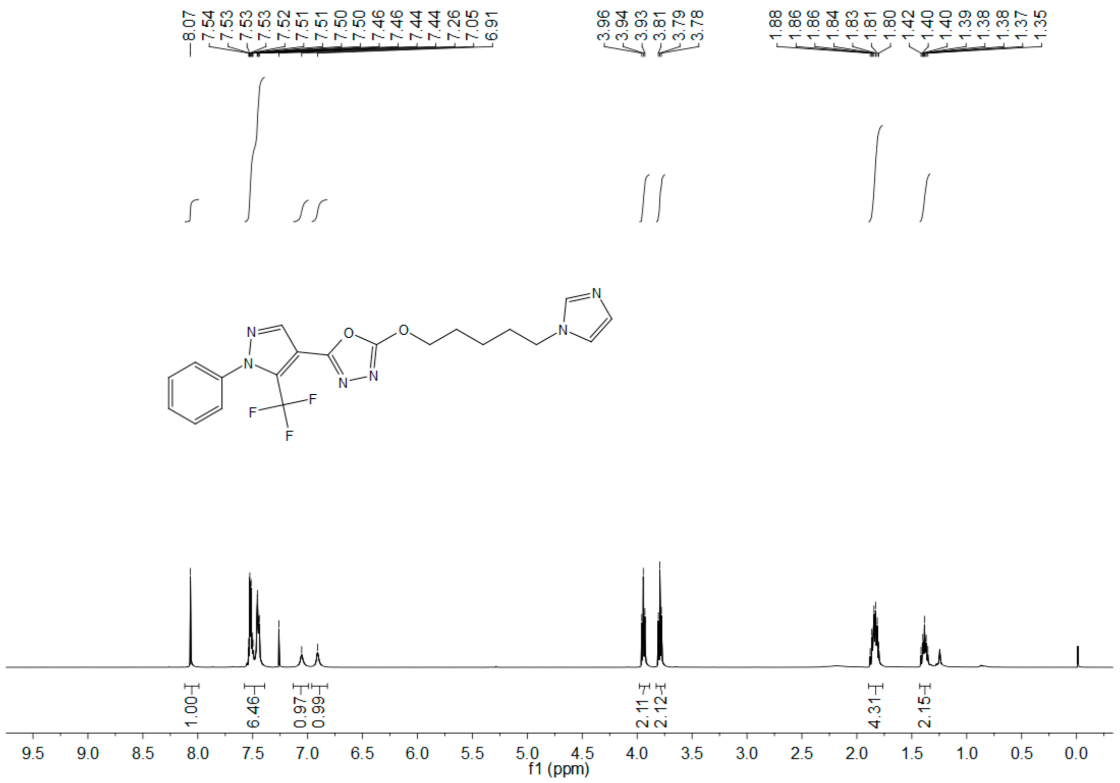

Figure S5. ^{1}H NMR spectrum of target compound **7b.**

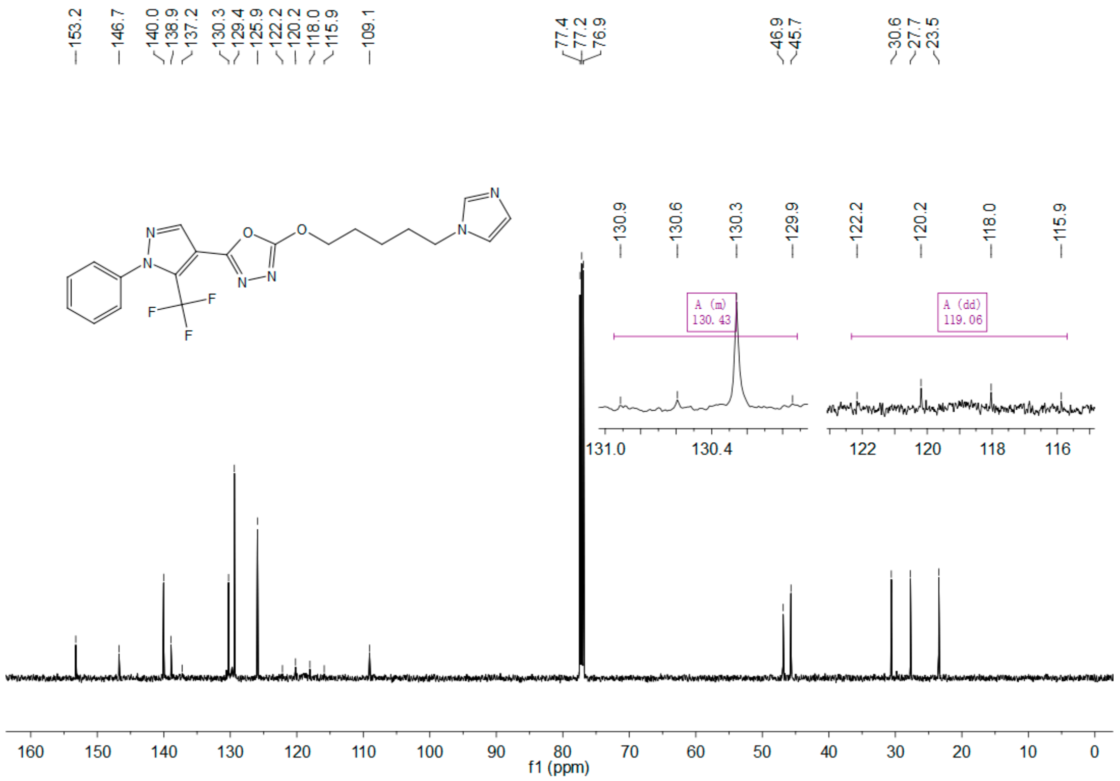

Figure S6. ^{13}C NMR spectrum of target compound **7b.**

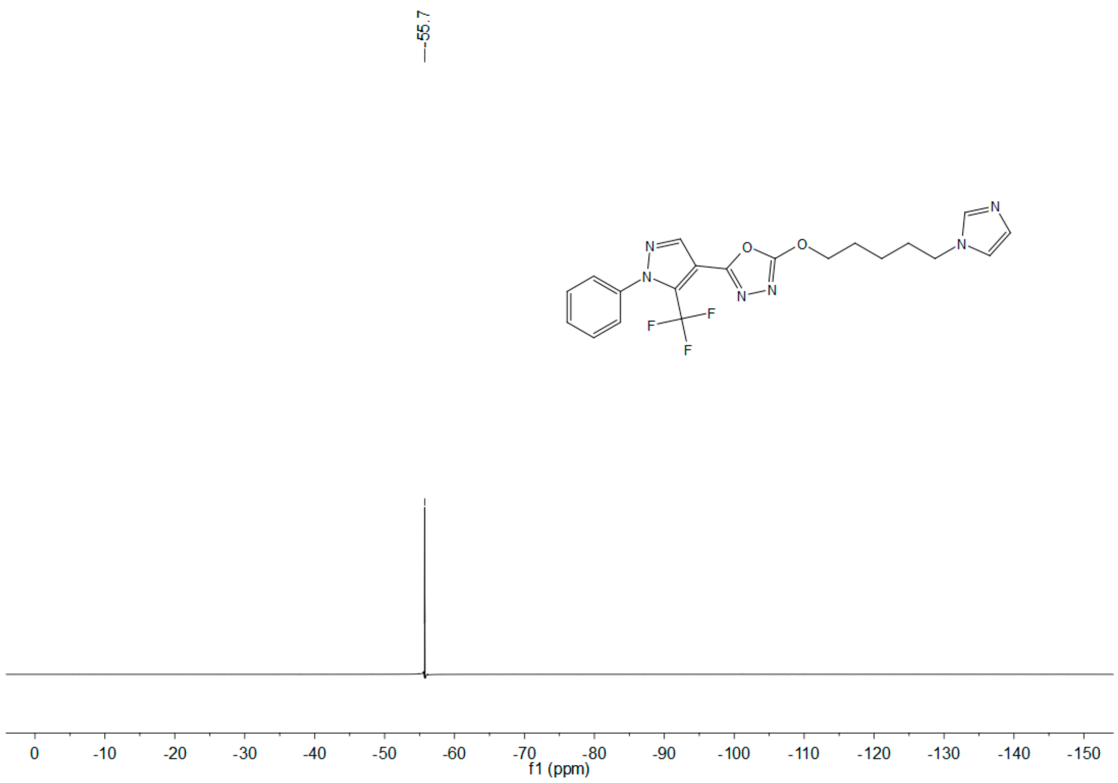

Figure S7. ^{19}F NMR spectrum of target compound **7b.**

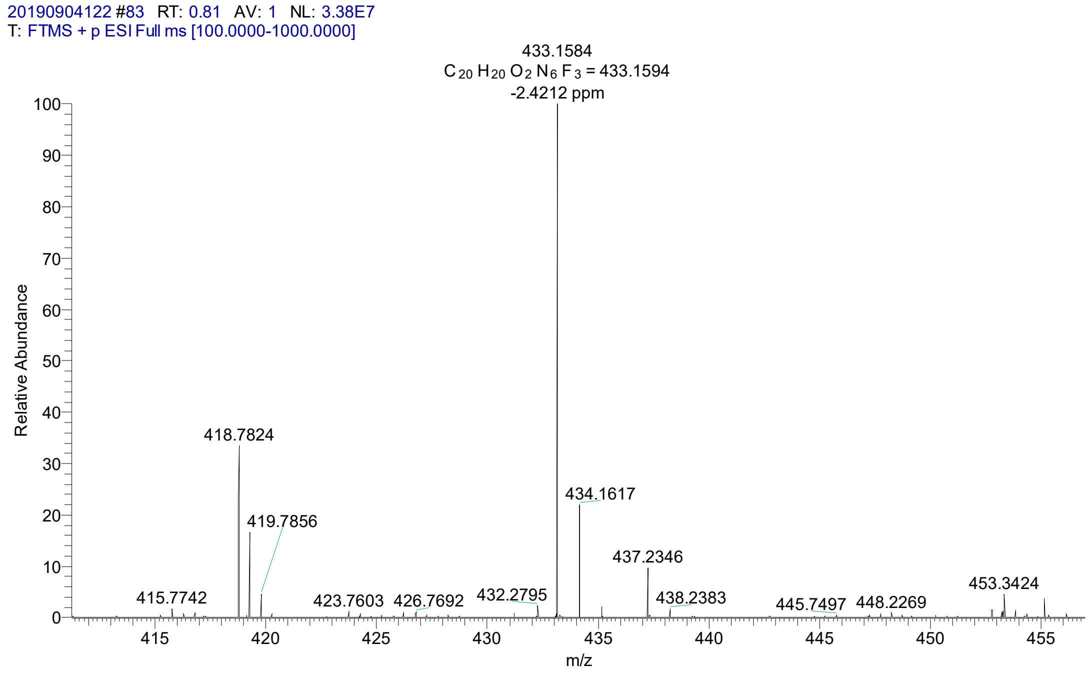

Figure S8. HRMS spectrum of target compound **7b.**

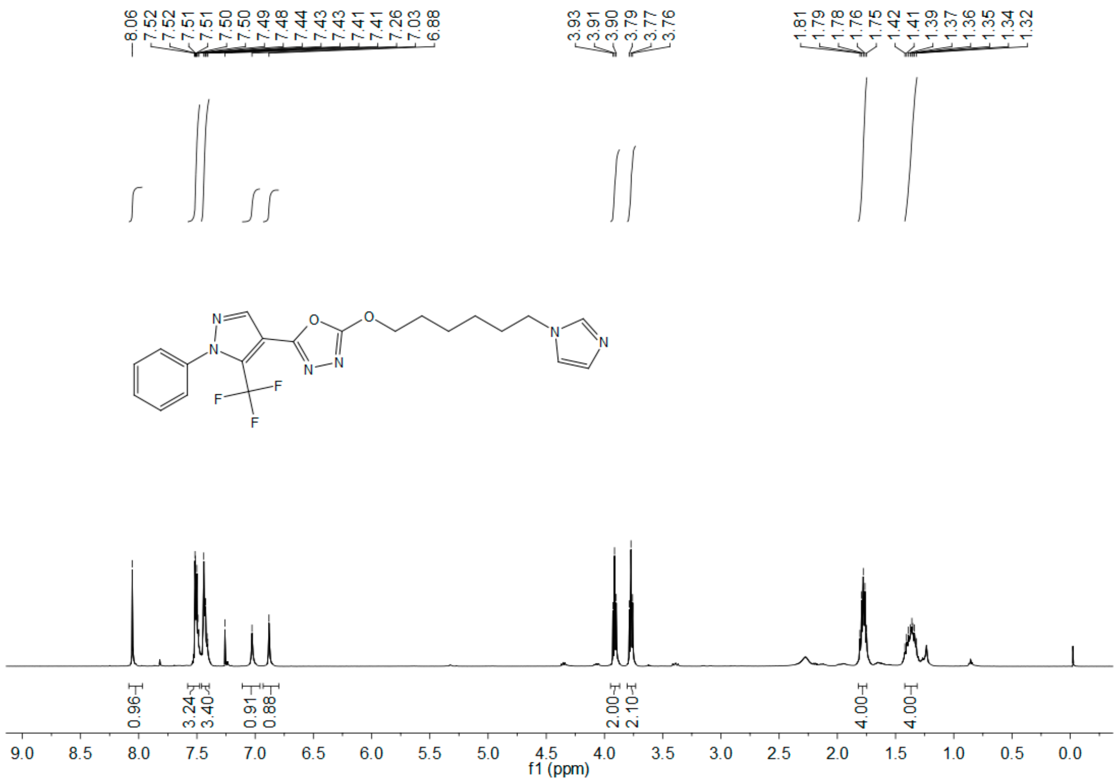

Figure S9. ^{1}H NMR spectrum of target compound **7c**

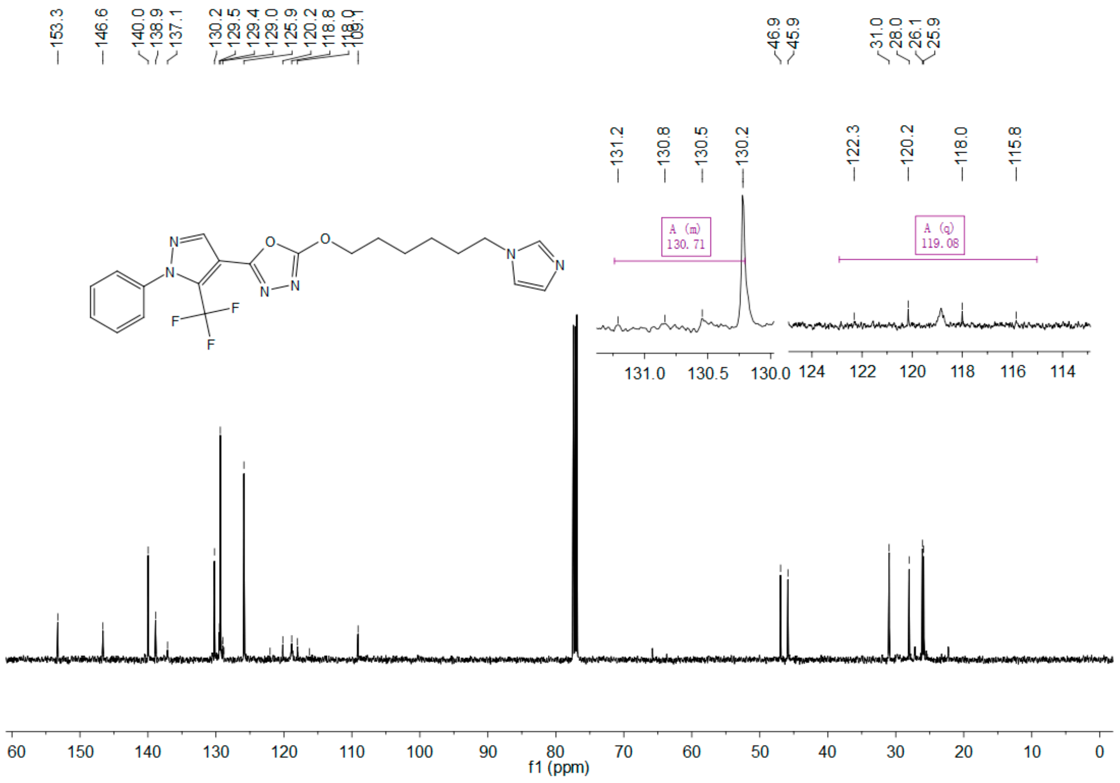

Figure S10. ^{13}C NMR spectrum of target compound **7c.**

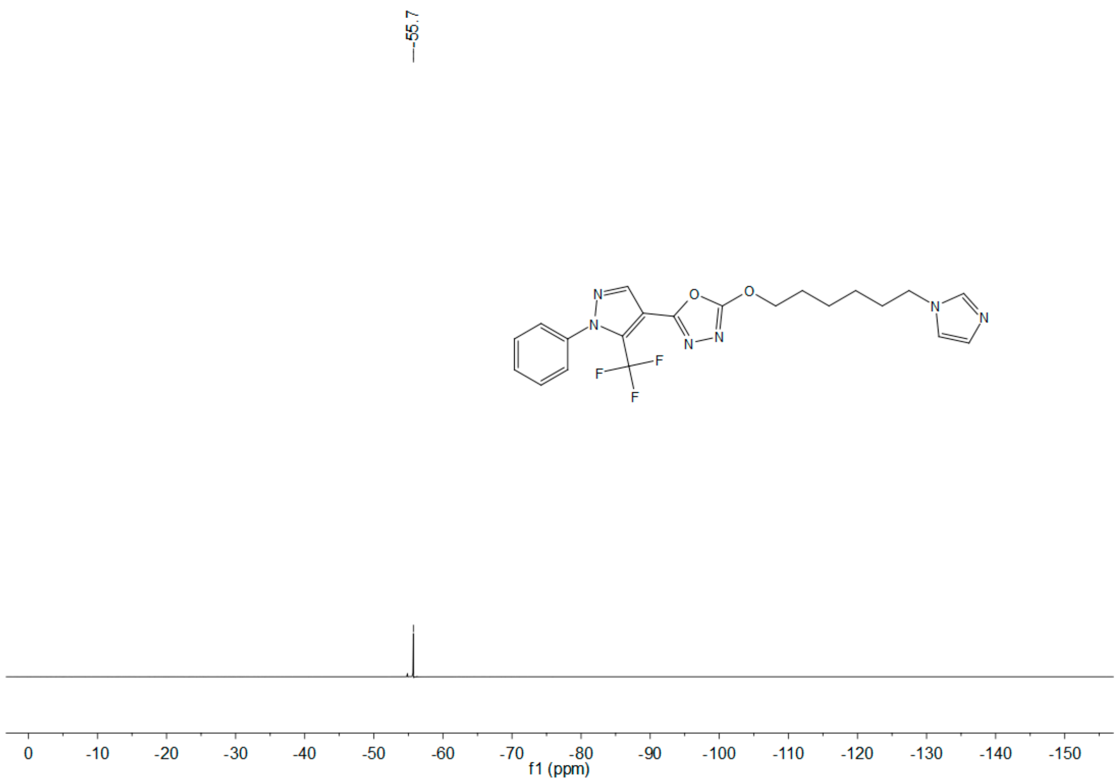

Figure S11. ^{19}F NMR spectrum of target compound **7c.**

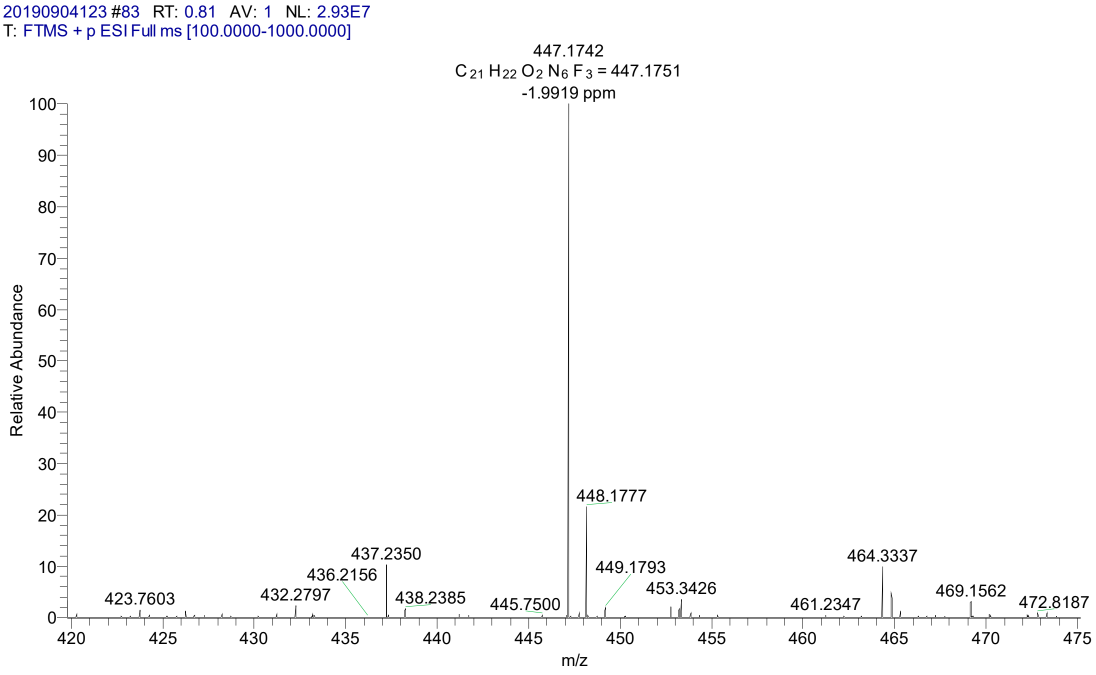

Figure S12. HRMS spectrum of target compound **7c.**

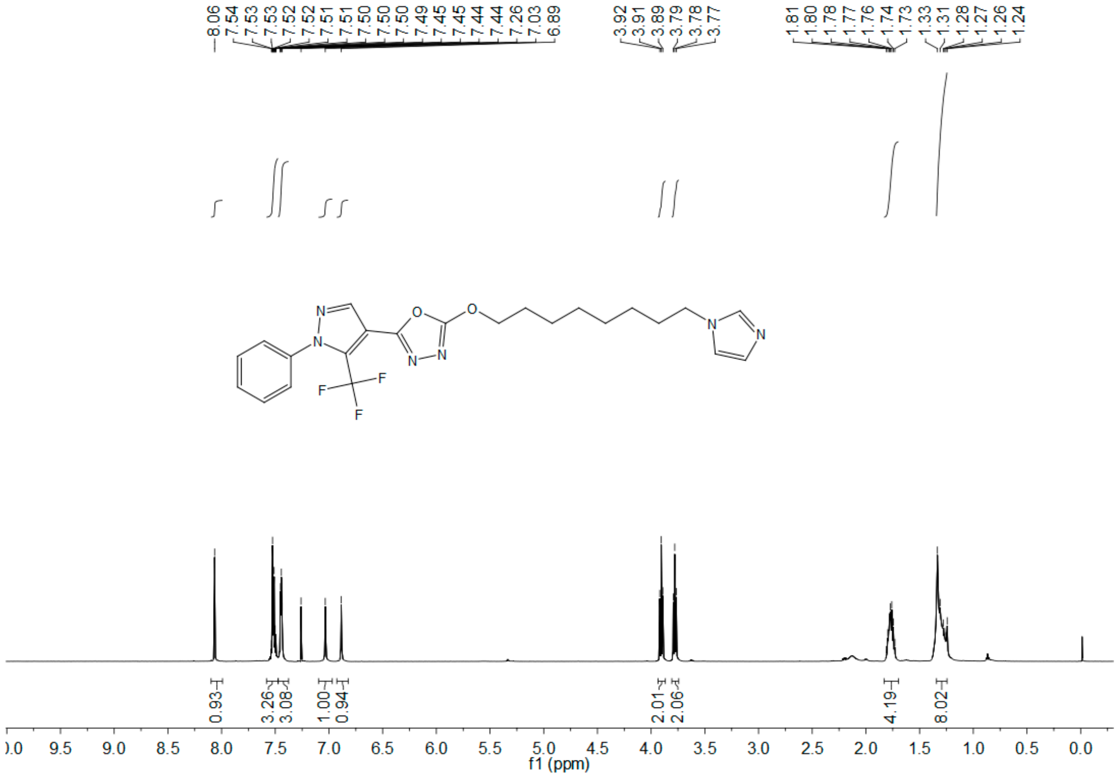

Figure S13. ^{1}H NMR spectrum of target compound **7d.**

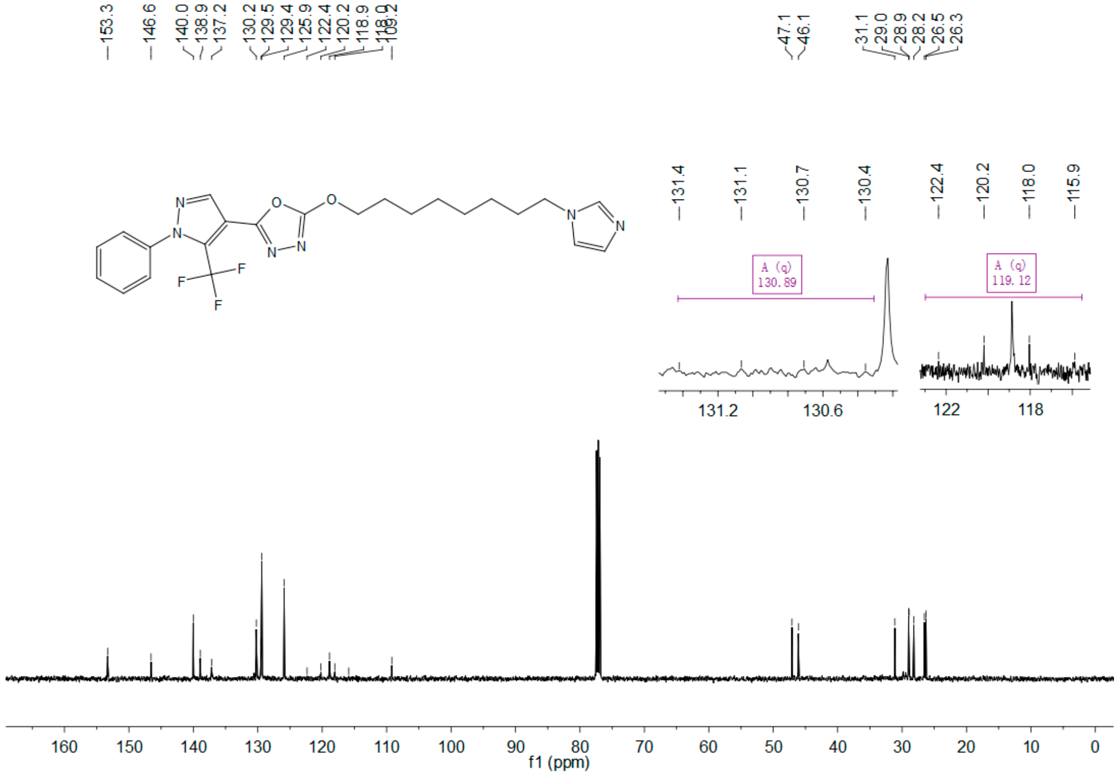

Figure S14. ^{13}C NMR spectrum of target compound **7d.**

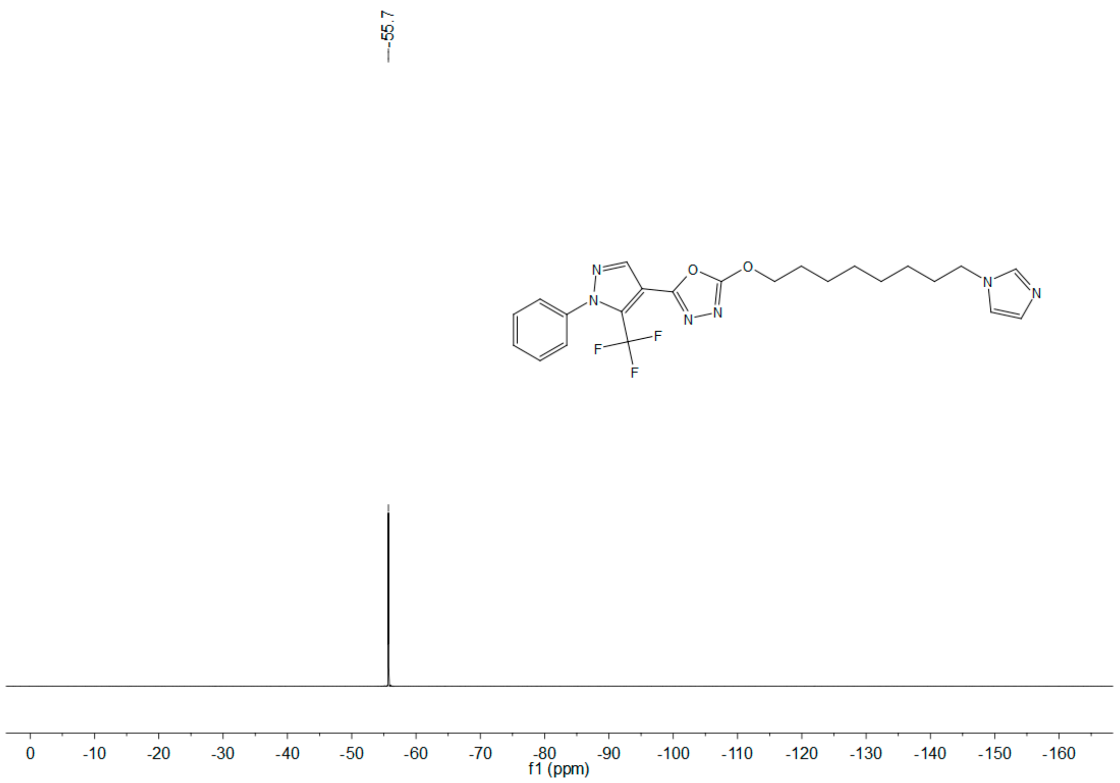

Figure S15. ^{19}F NMR spectrum of target compound **7d.**

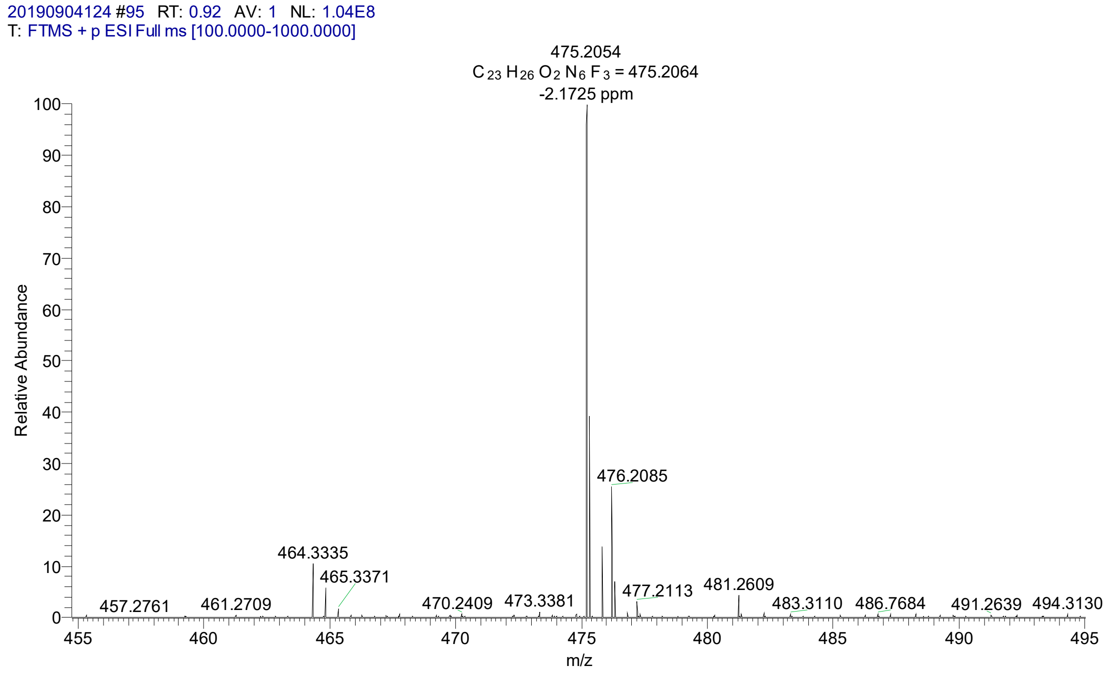

Figure S16. HRMS spectrum of target compound **7d.**

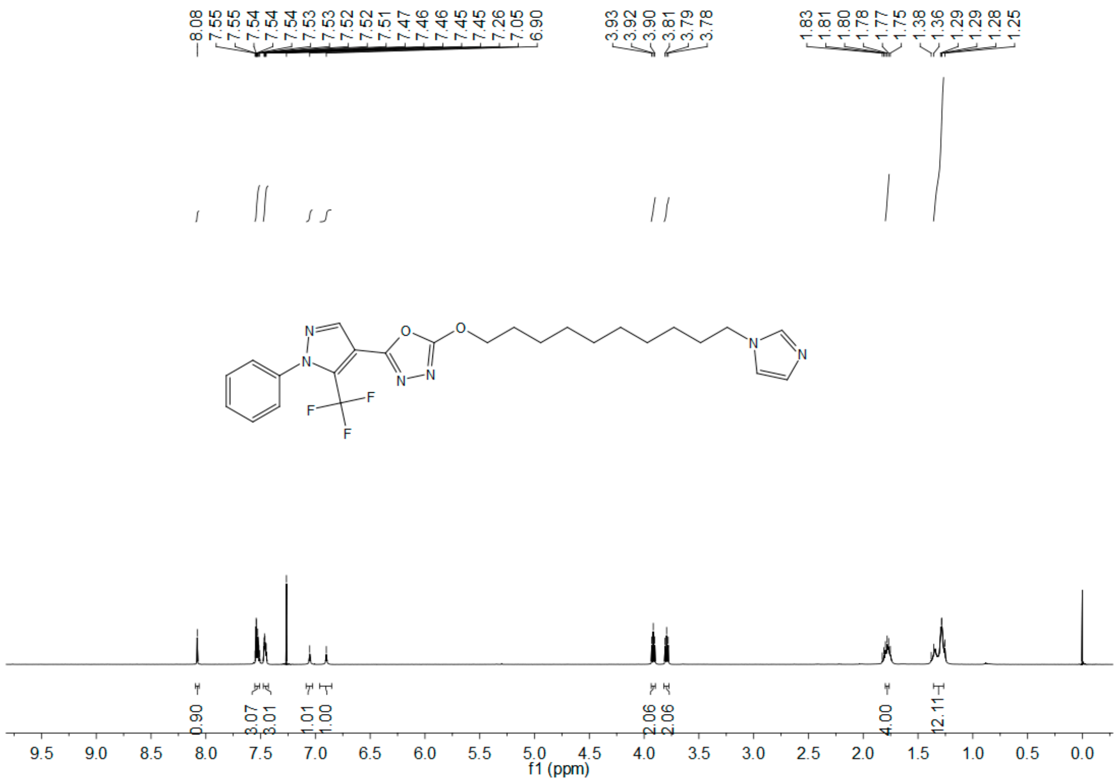

Figure S17. ^{1}H NMR spectrum of target compound **7e.**

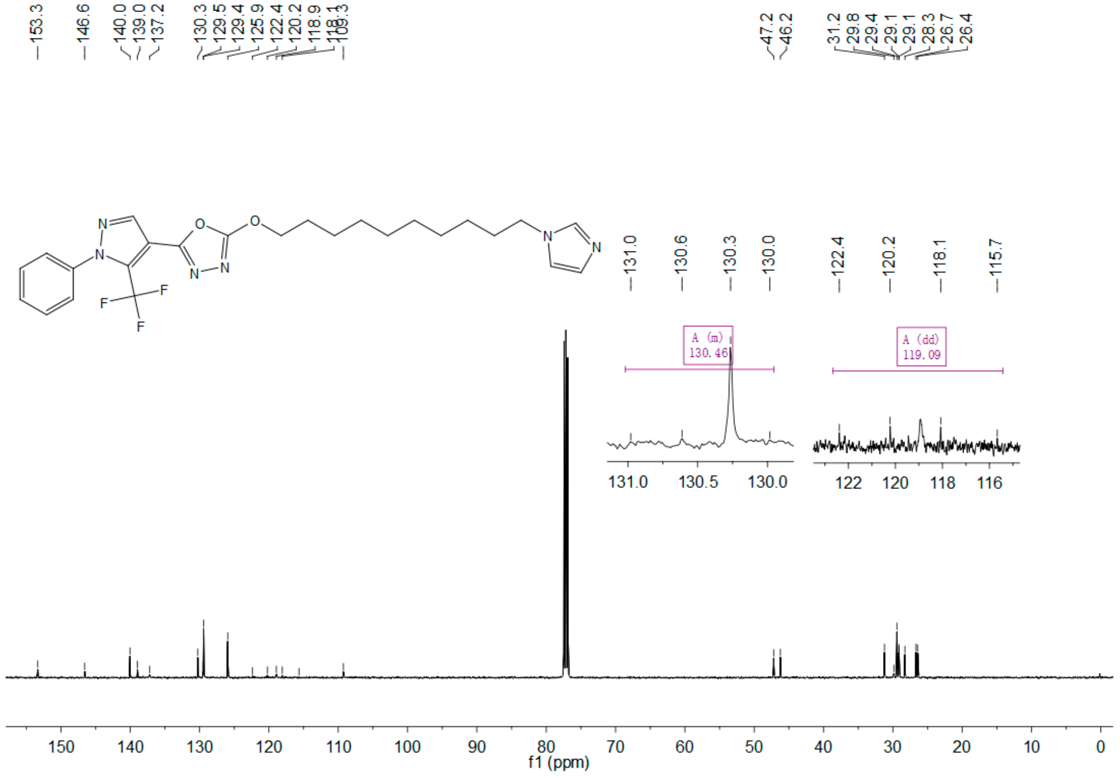

Figure S18. ^{13}C NMR spectrum of target compound **7e.**

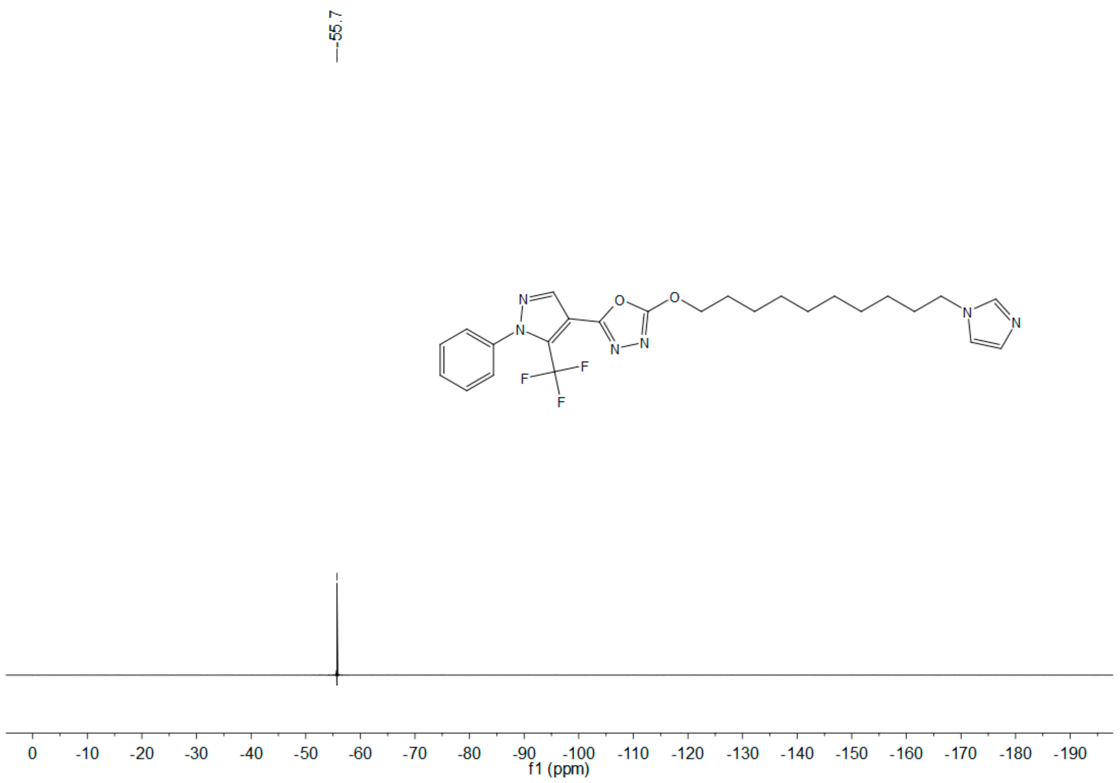

Figure S19. ^{19}F NMR spectrum of target compound **7e.**

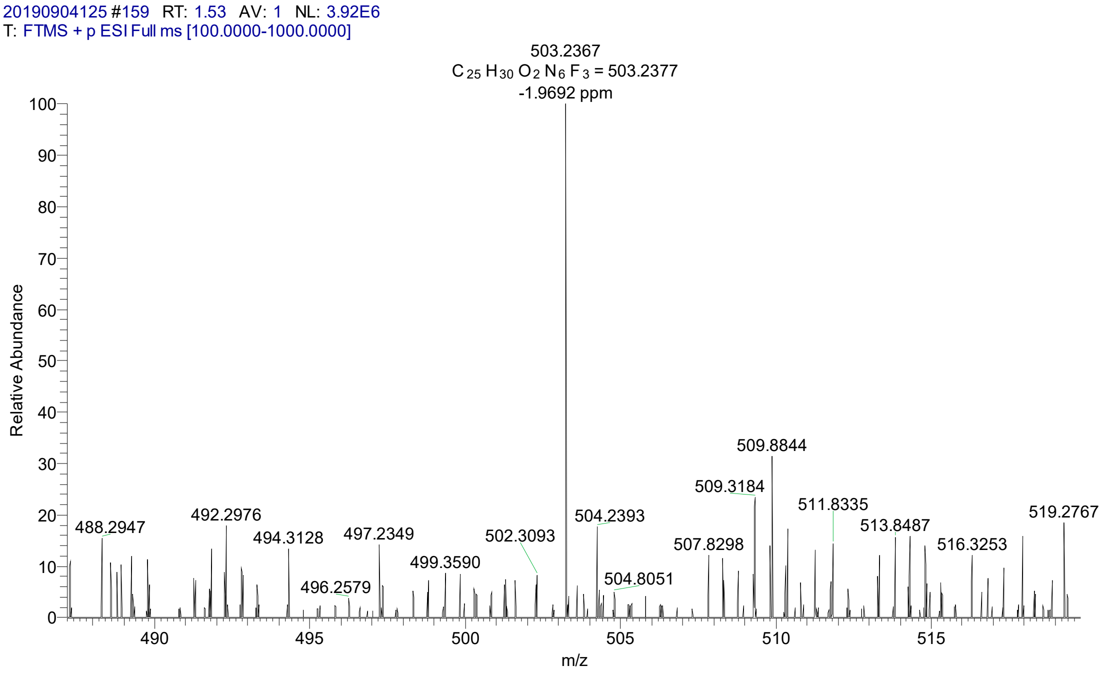

Figure S20. HRMS spectrum of target compound **7e.**

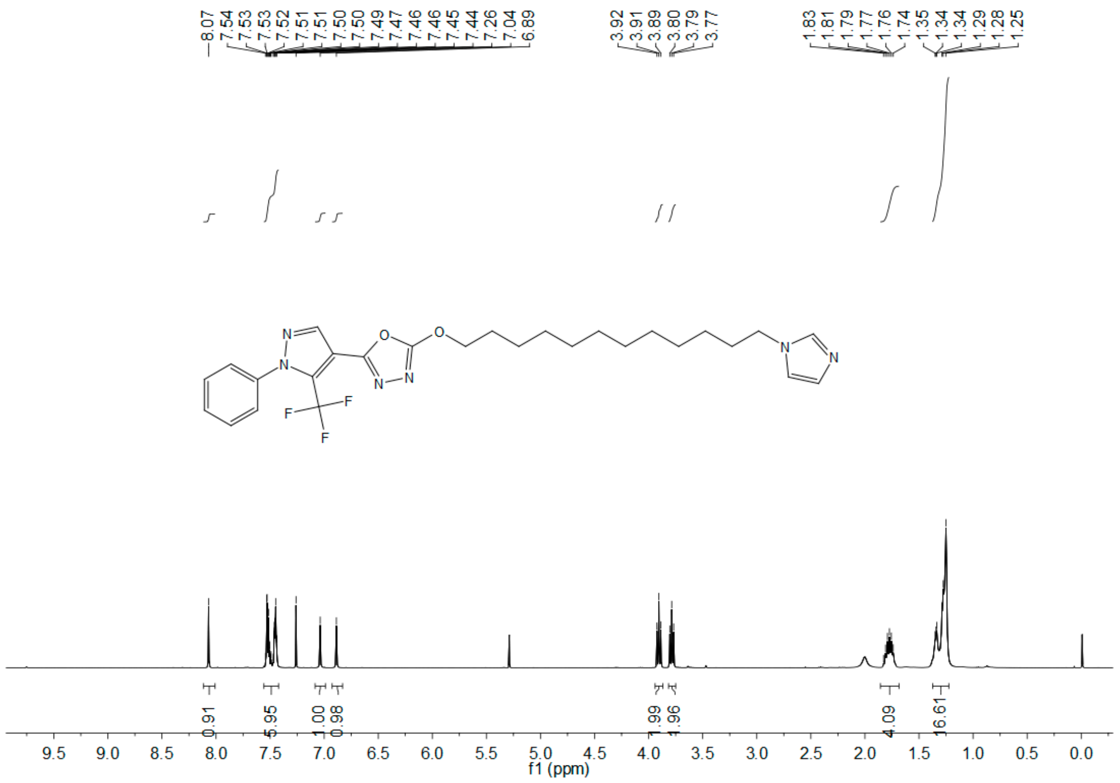

Figure S21. ^{1}H NMR spectrum of target compound **7f.**

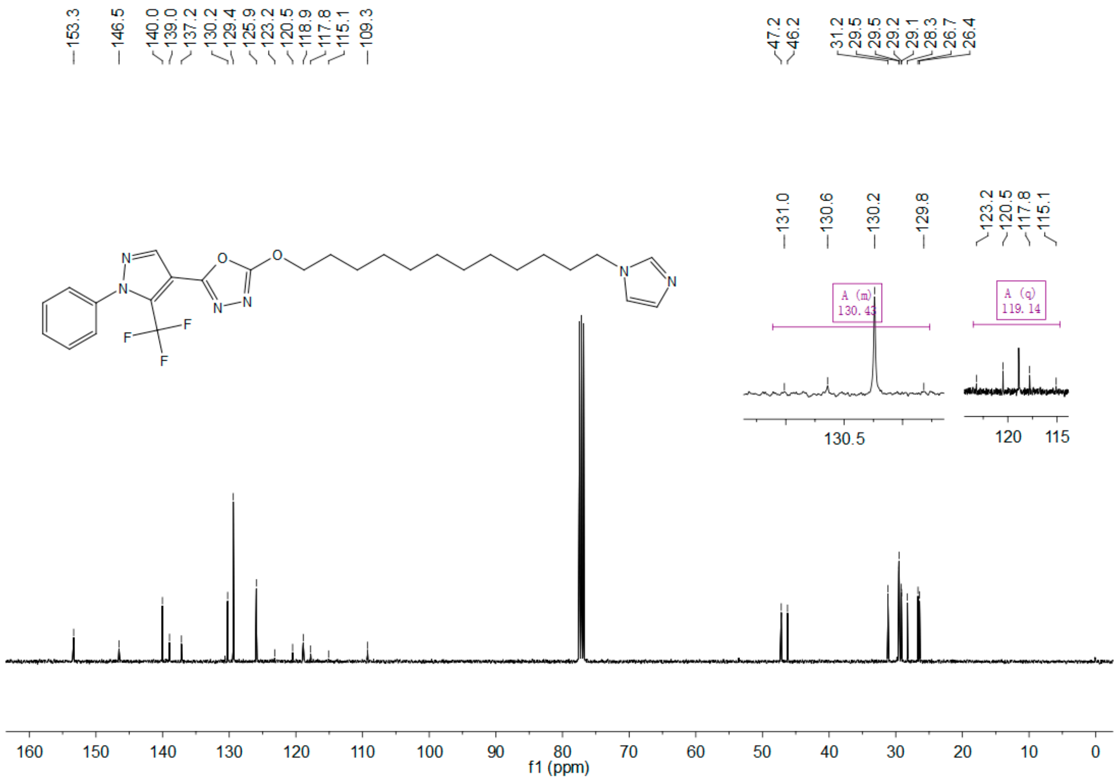

Figure S22. ^{13}C NMR spectrum of target compound **7f.**

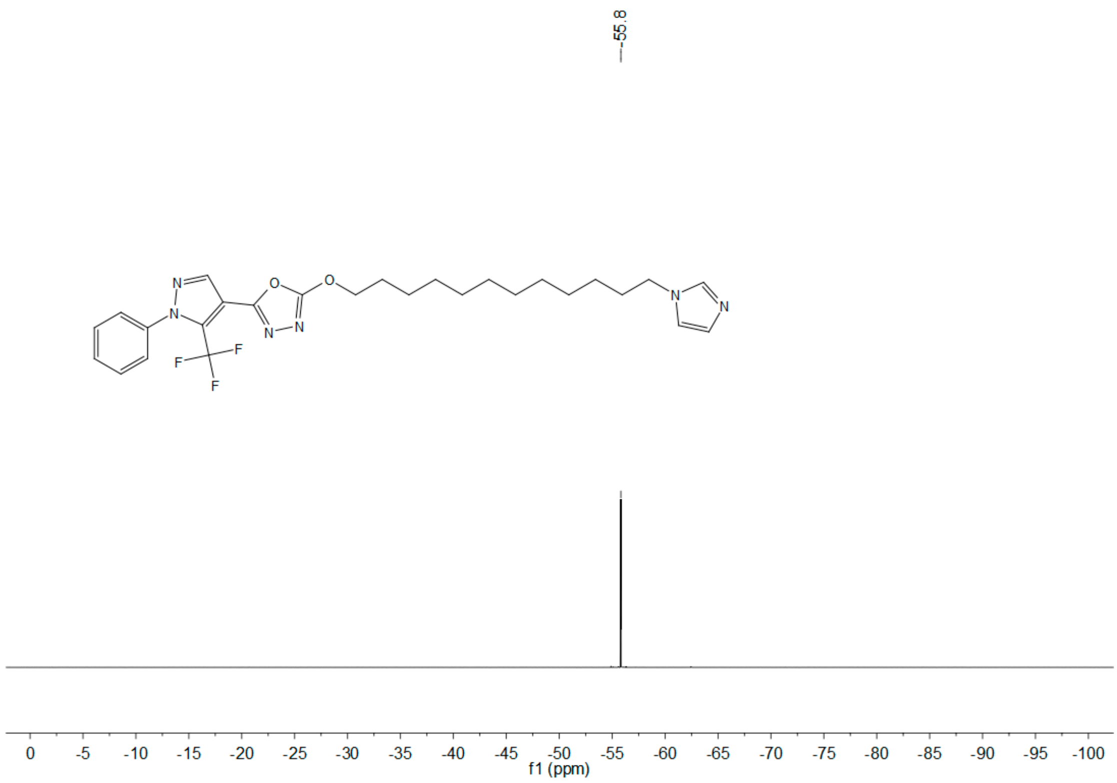

Figure S23. ^{19}F NMR spectrum of target compound **7f.**

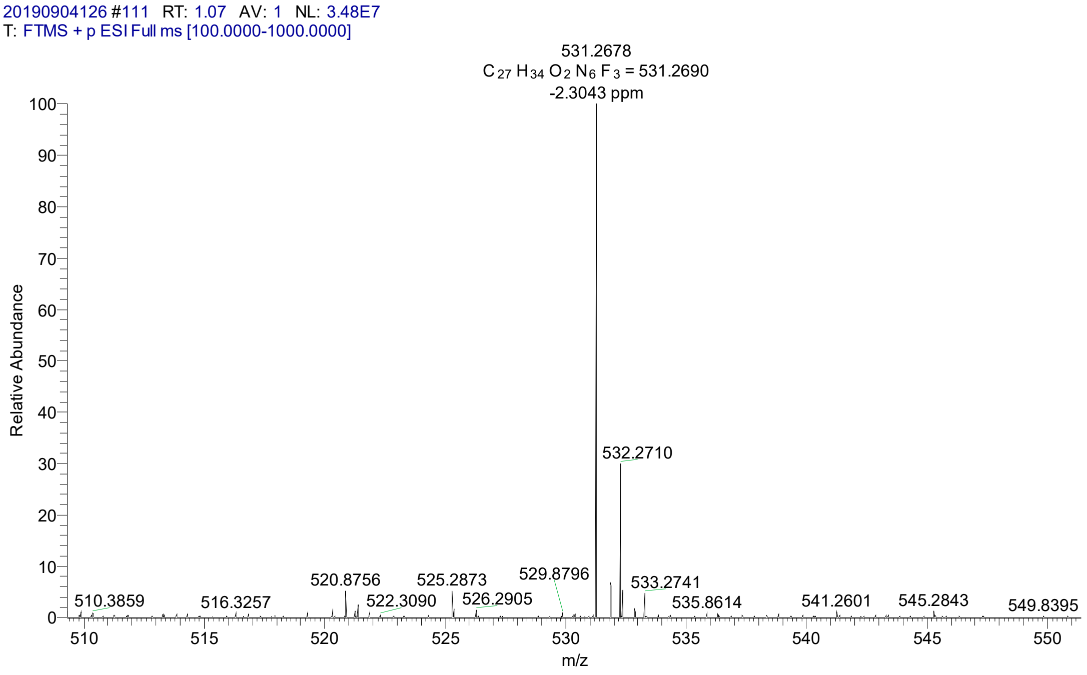

Figure S24. HRMS spectrum of target compound **7f.**

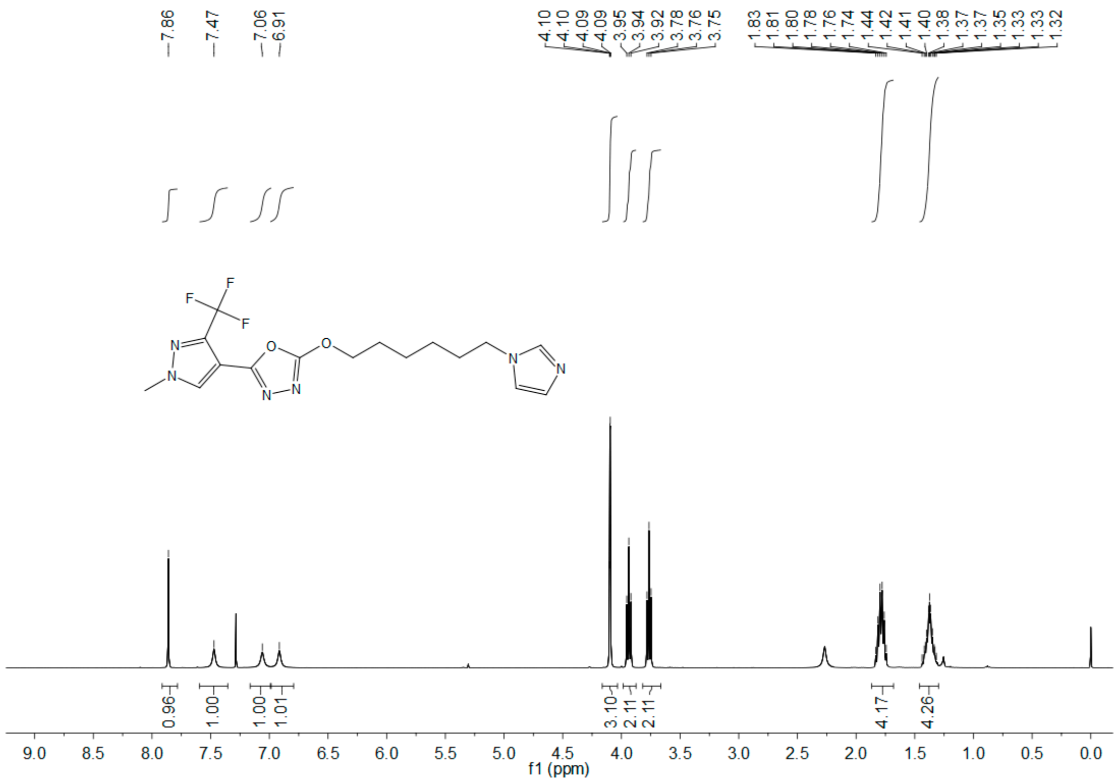

Figure S25. ^{1}H NMR spectrum of target compound **8a.**

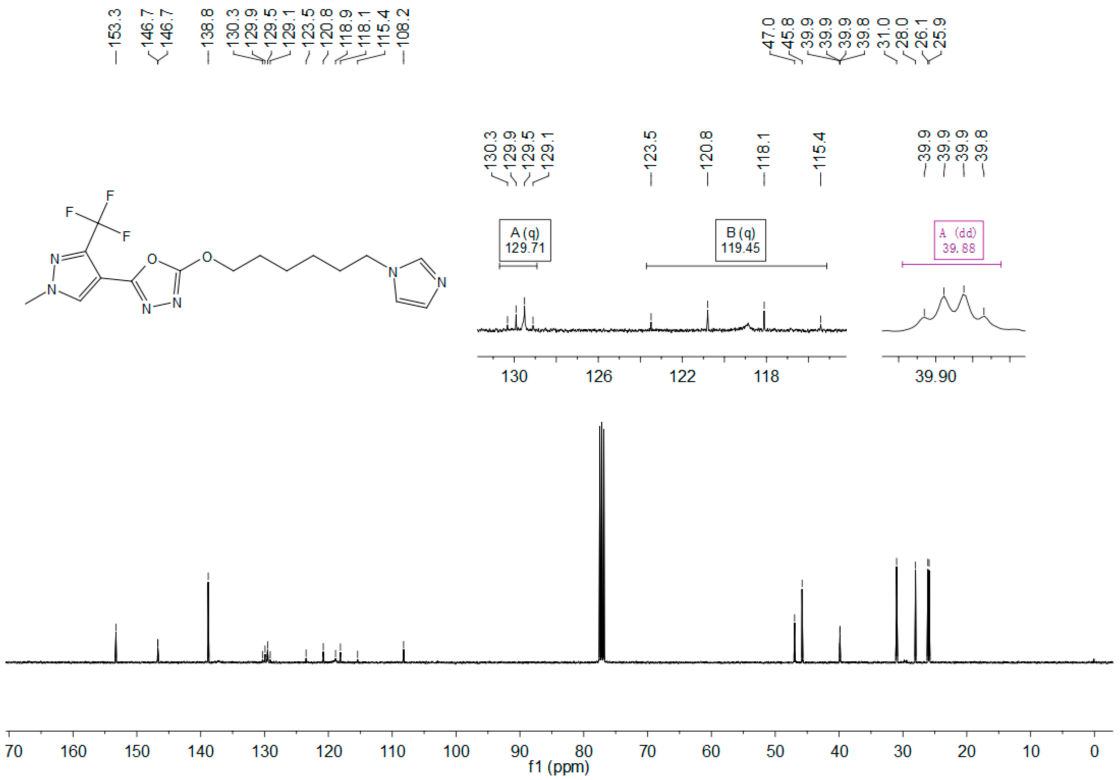

Figure S26. ^{13}C NMR spectrum of target compound **8a.**

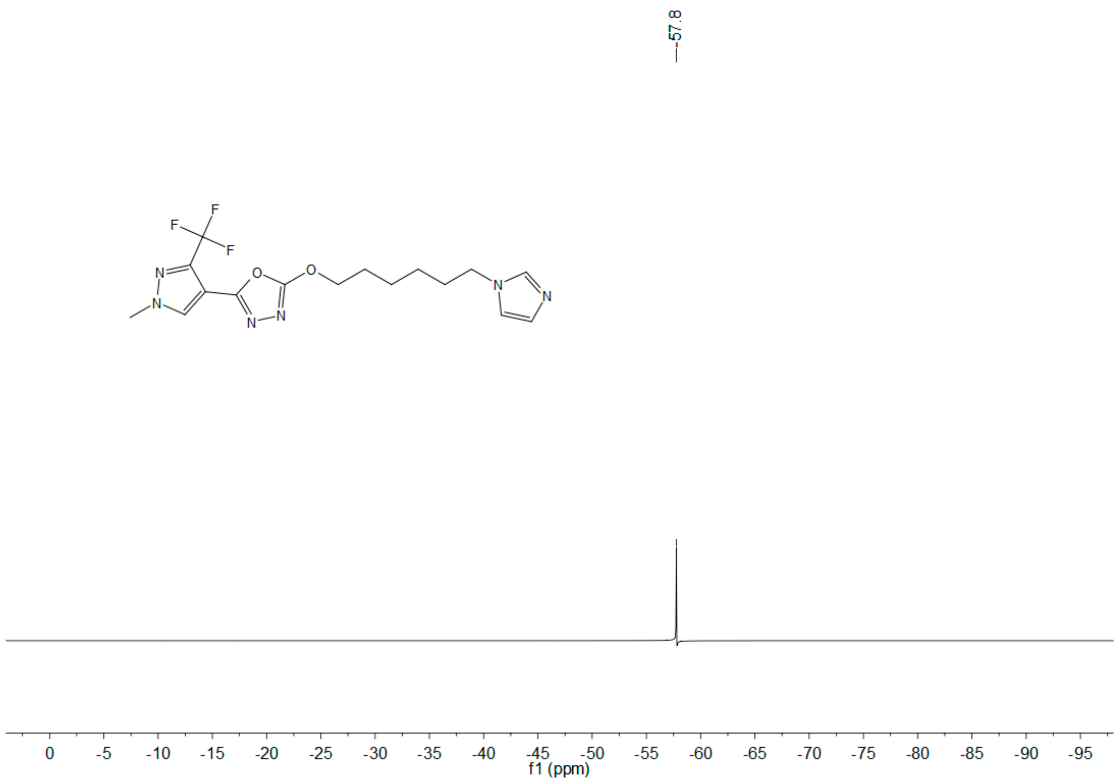

Figure S26. ^{19}F NMR spectrum of target compound **8a.**

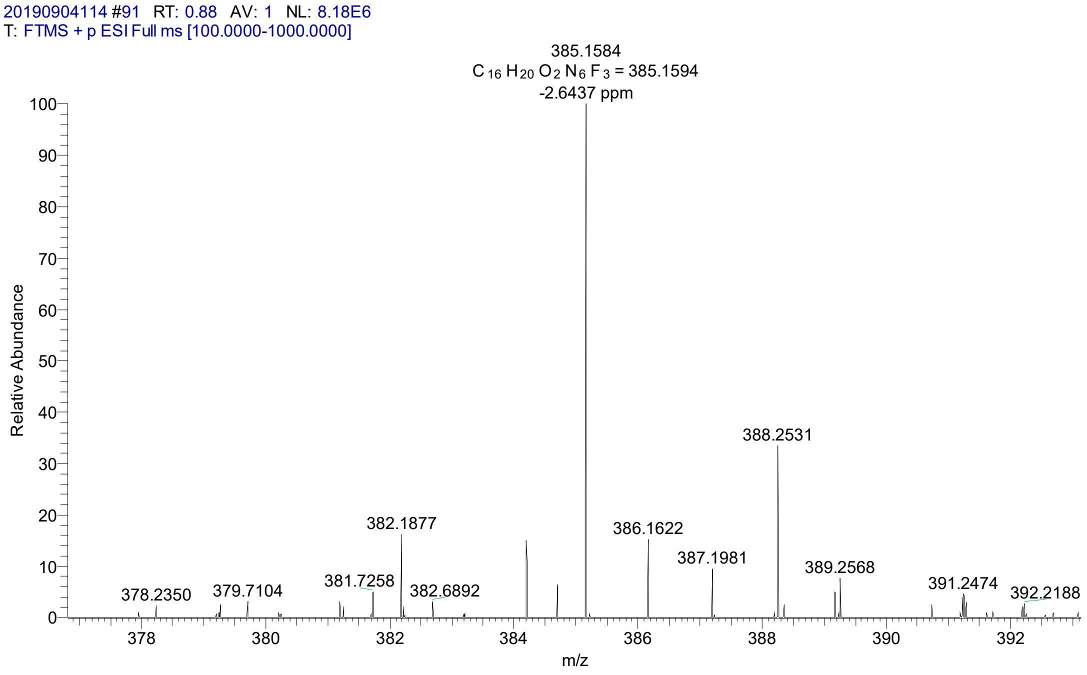

Figure S28. HRMS spectrum of target compound **8a.**

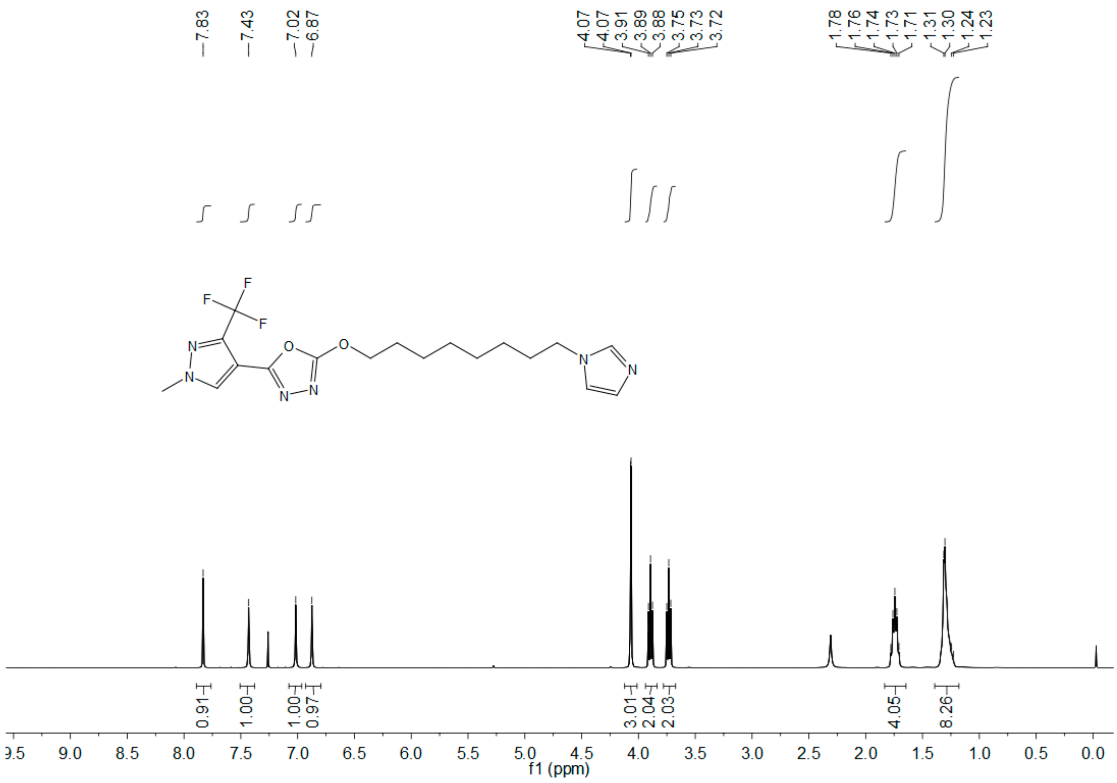

Figure S29. ^{1}H NMR spectrum of target compound **8b.**

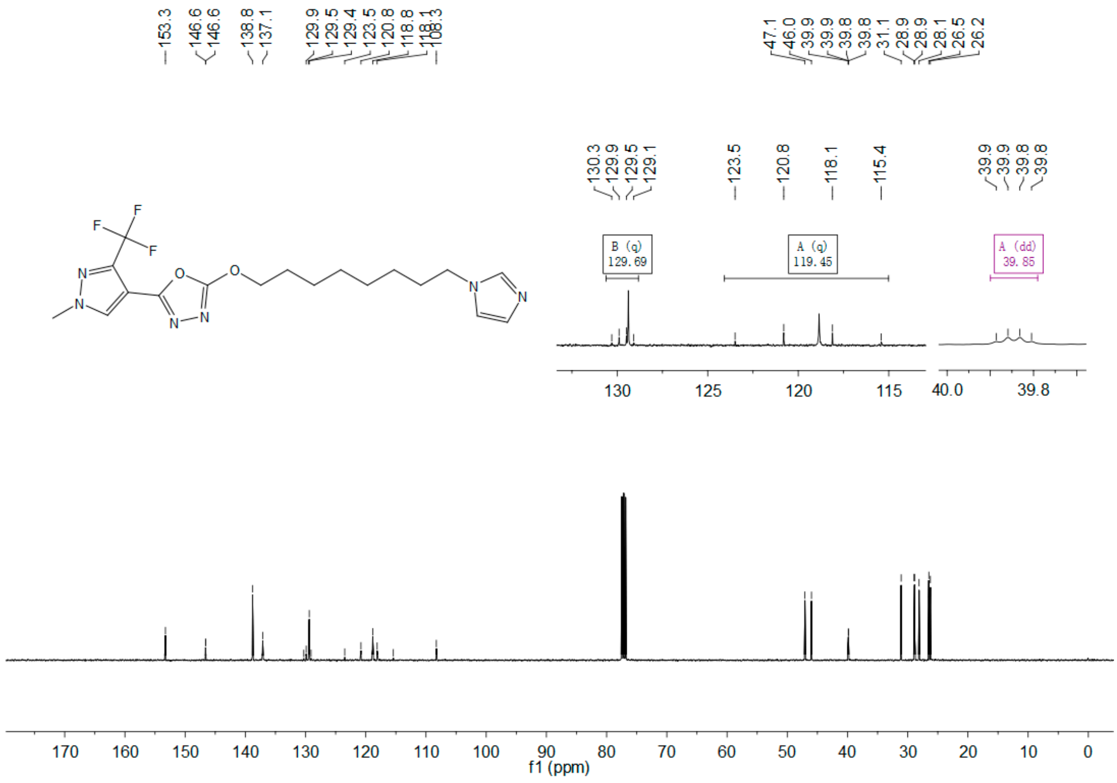

Figure S30. ^{13}C NMR spectrum of target compound **8b.**

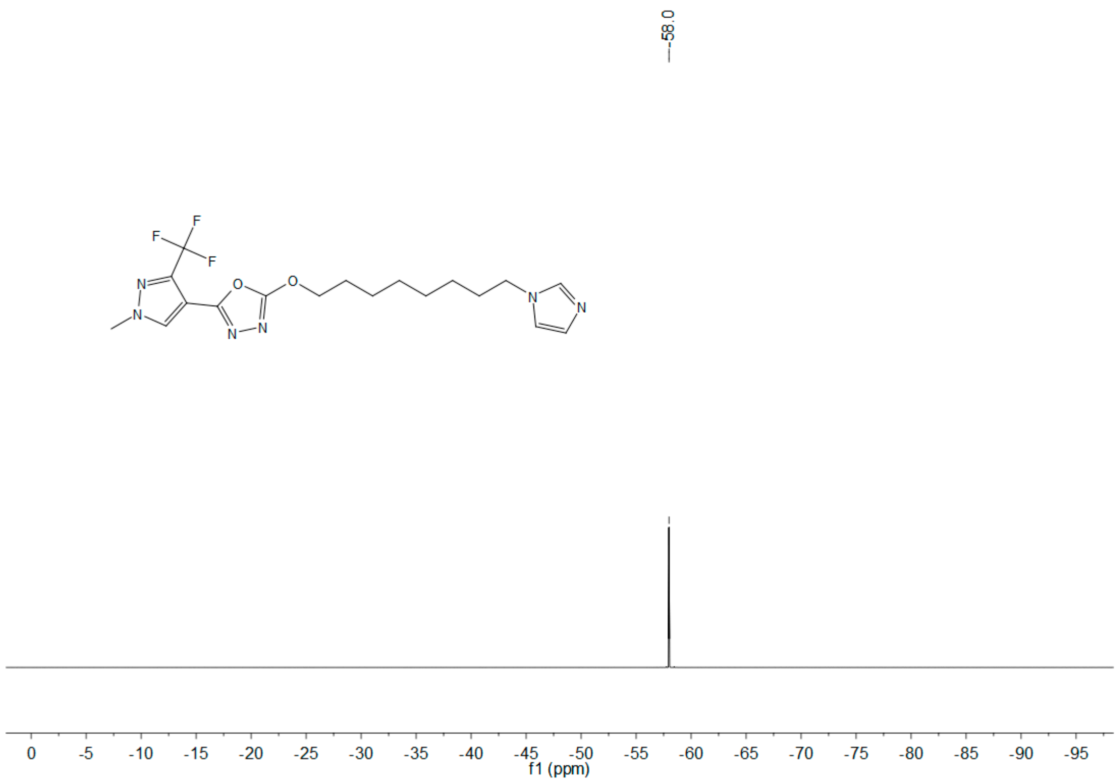

Figure S31. ^{19}F NMR spectrum of target compound **8b.**

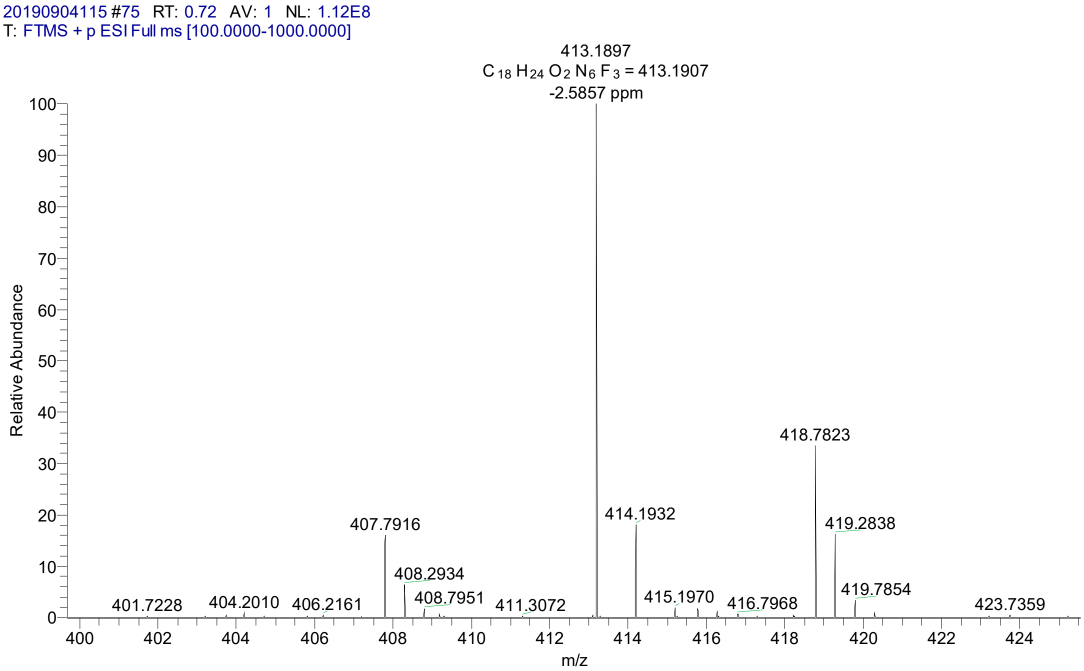

Figure S32. HRMS spectrum of target compound **8b.**

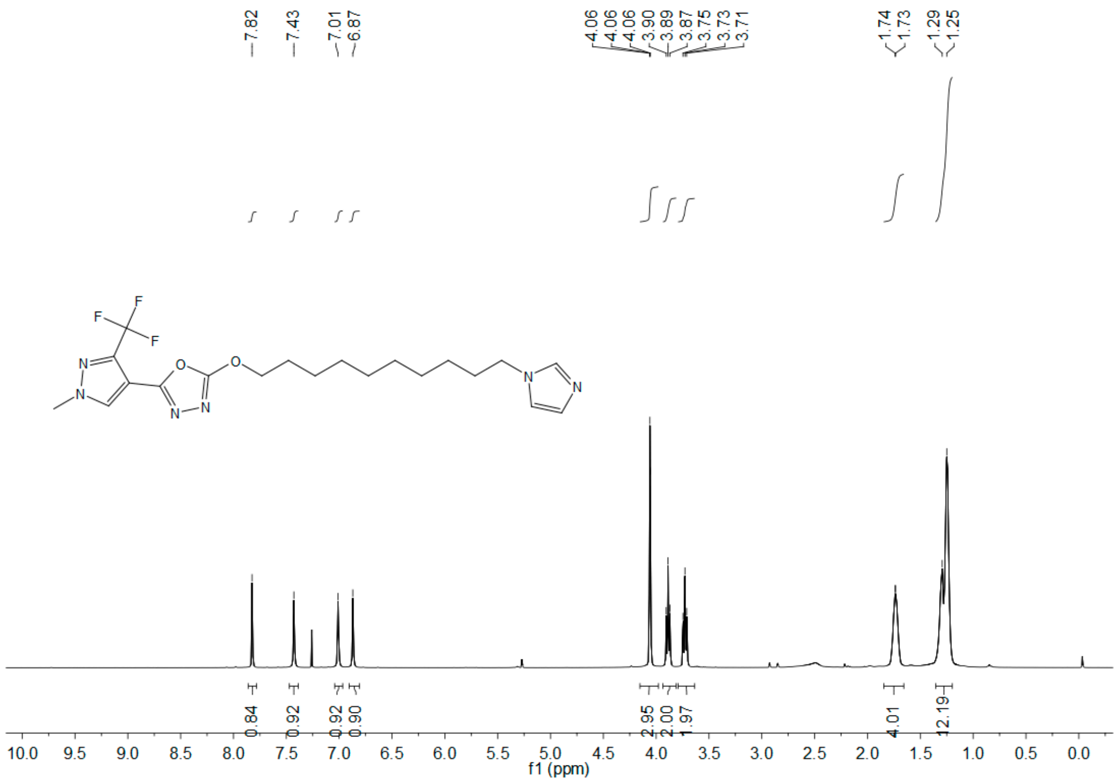

Figure S33. ^{1}H NMR spectrum of target compound **8c.**

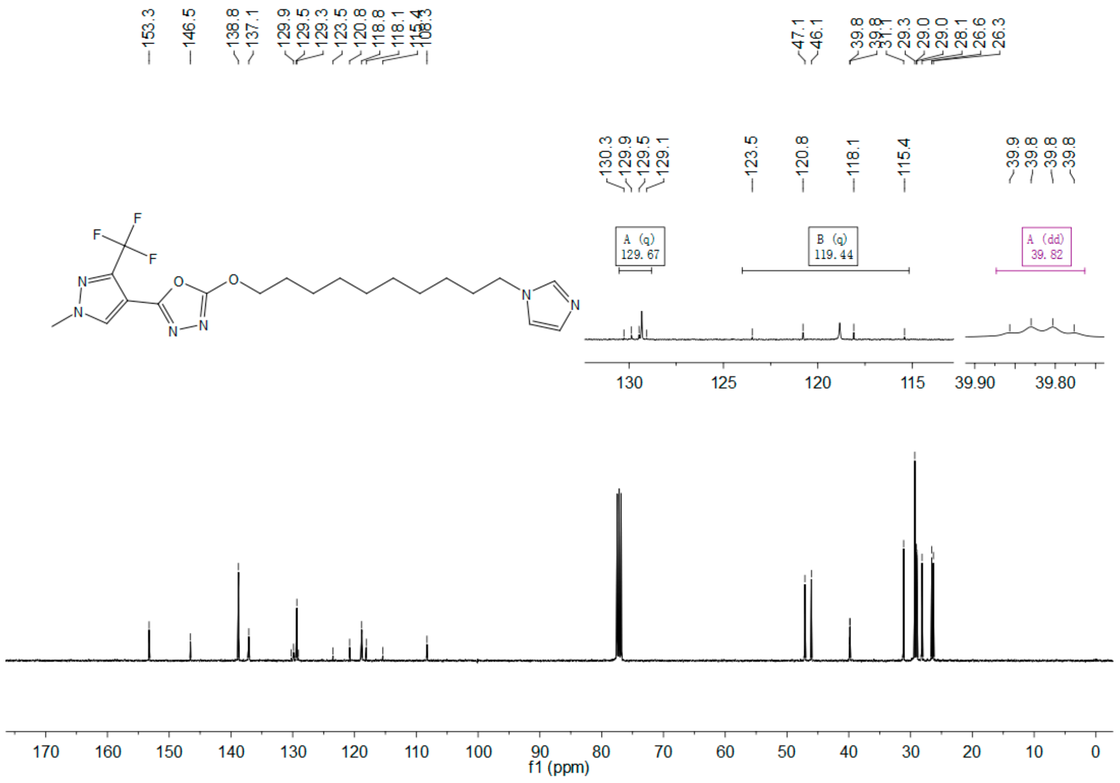

Figure S34. ^{13}C NMR spectrum of target compound **8c.**

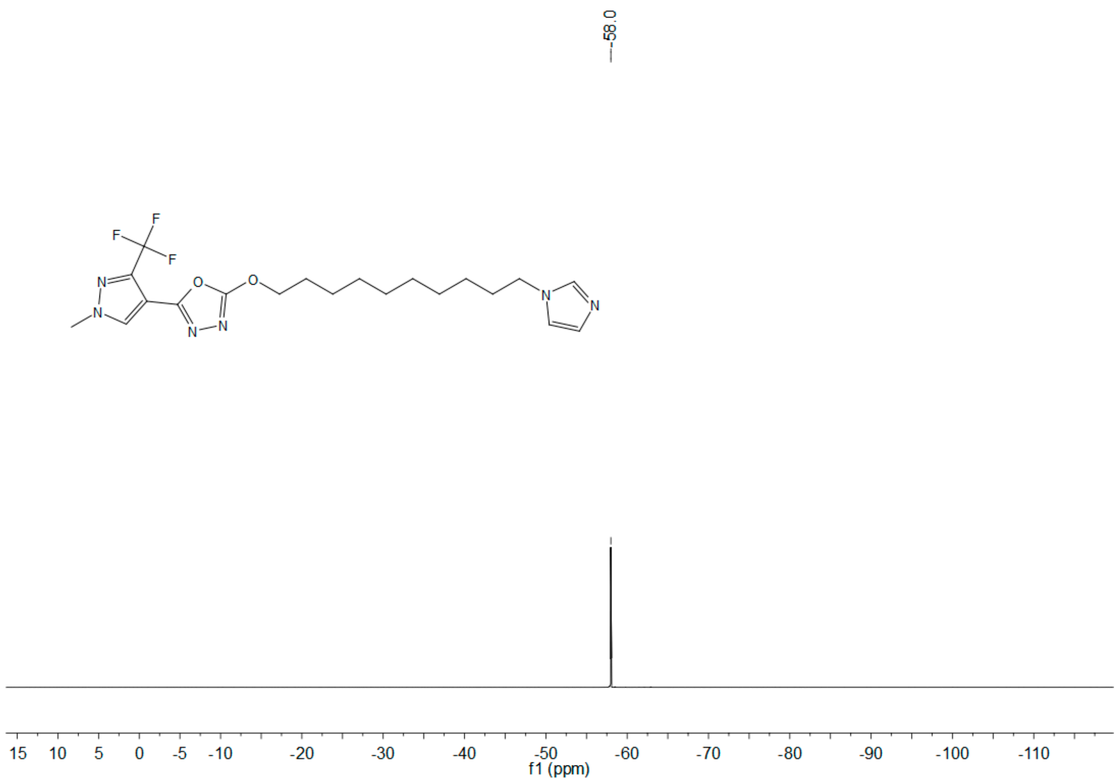

Figure S35. ^{19}F NMR spectrum of target compound **8c.**

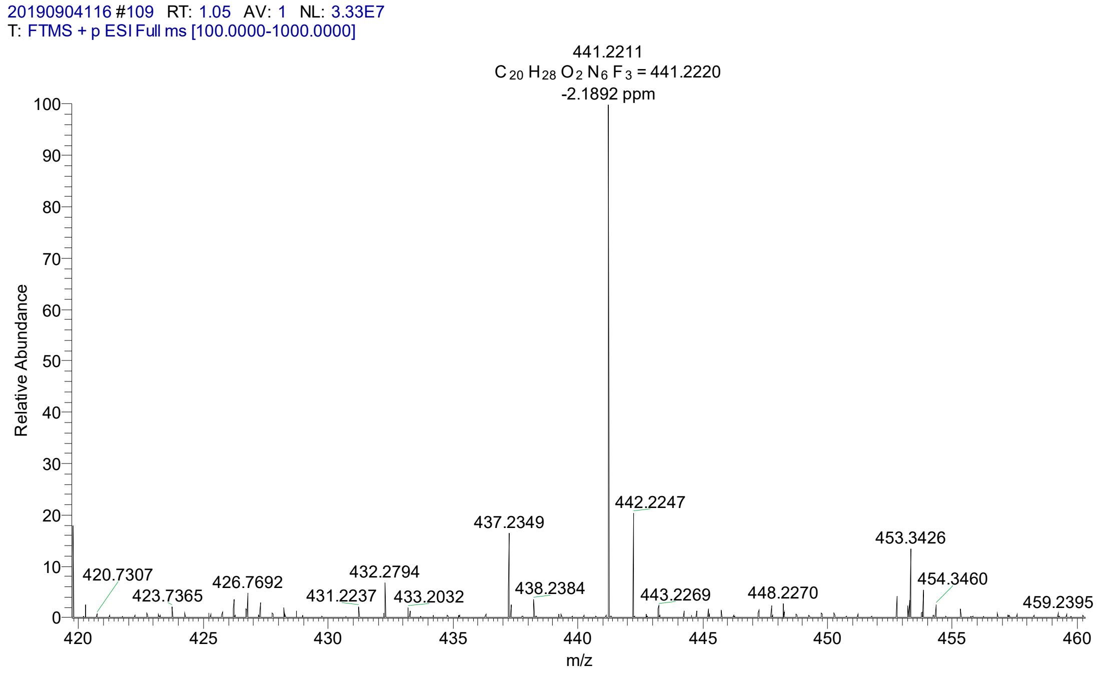

Figure S36. HRMS spectrum of target compound **8c.**

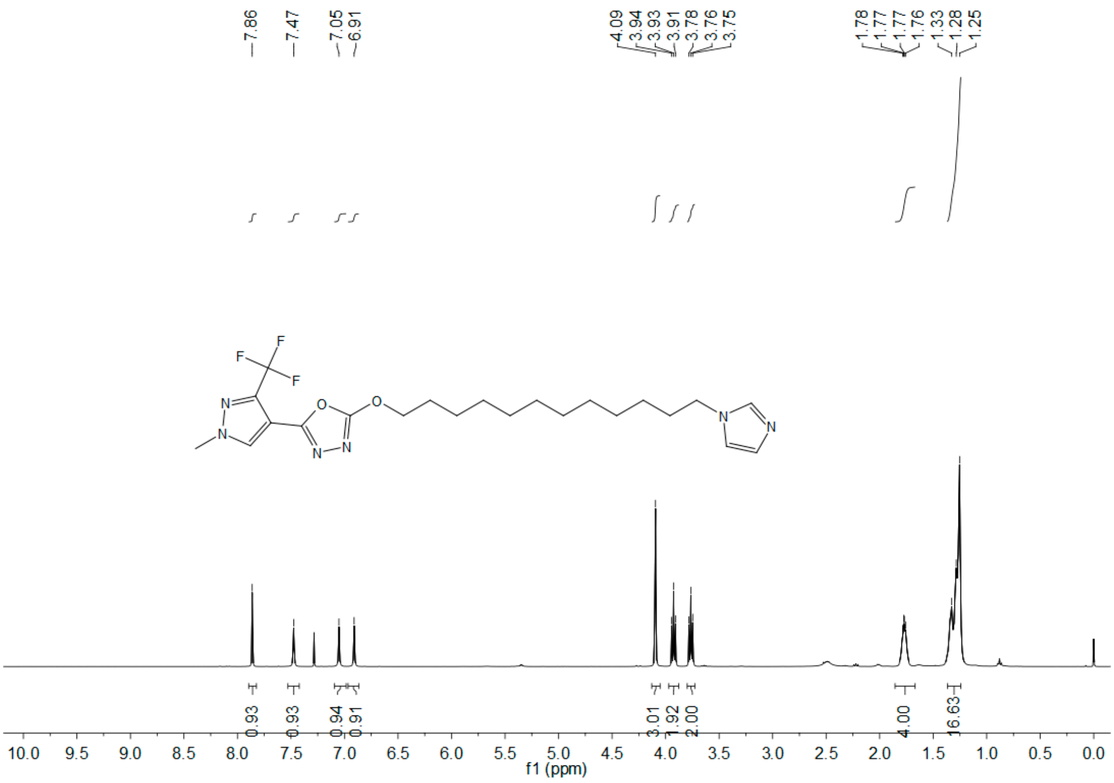

Figure S37. ^{1}H NMR spectrum of target compound **8d.**

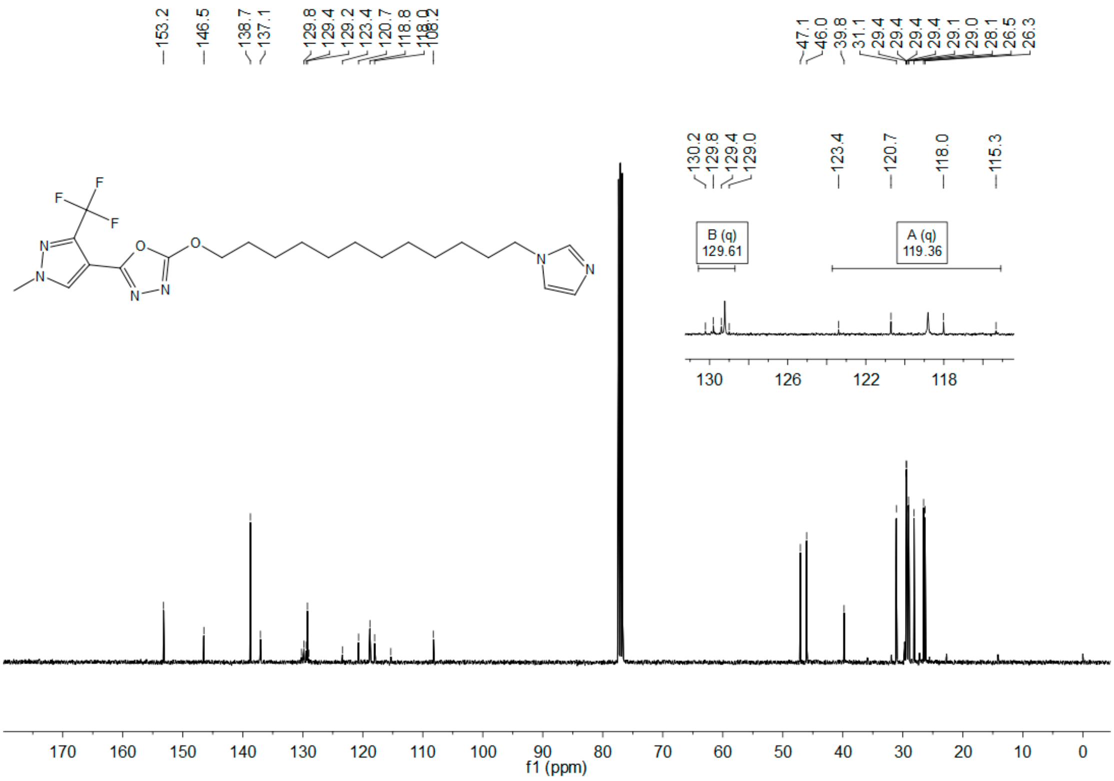

Figure S38. ^{13}C NMR spectrum of target compound **8d.**

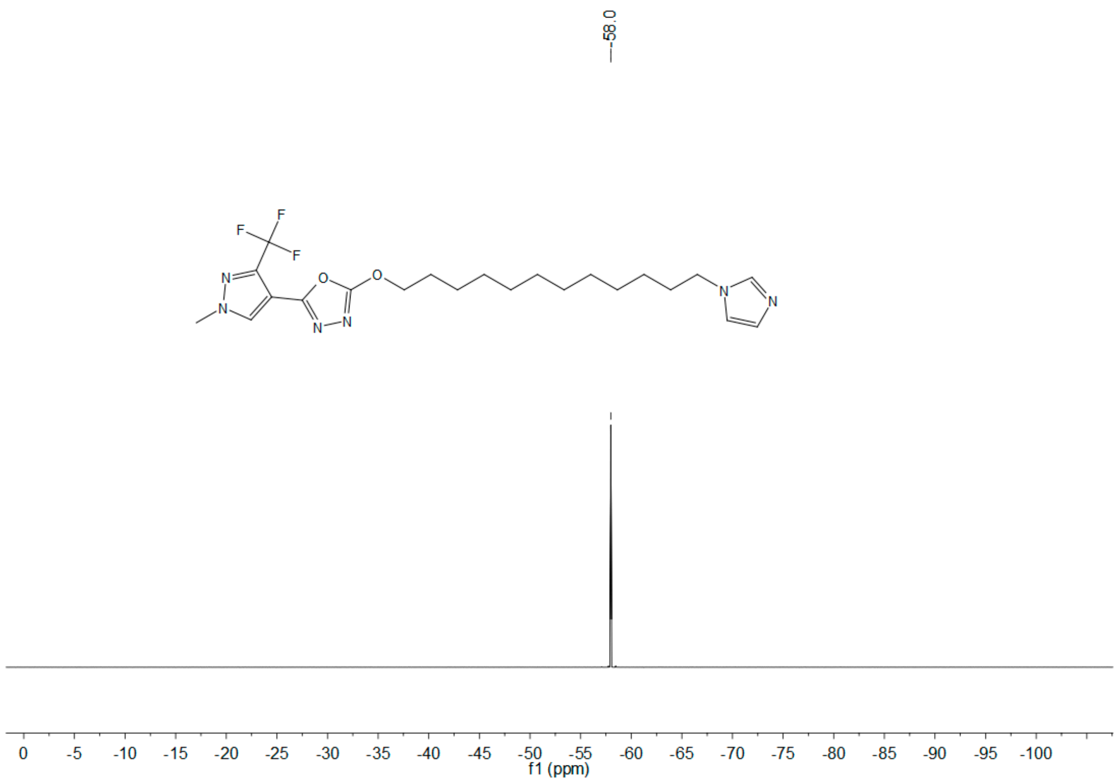

Figure S39. ^{19}F NMR spectrum of target compound **8d.**

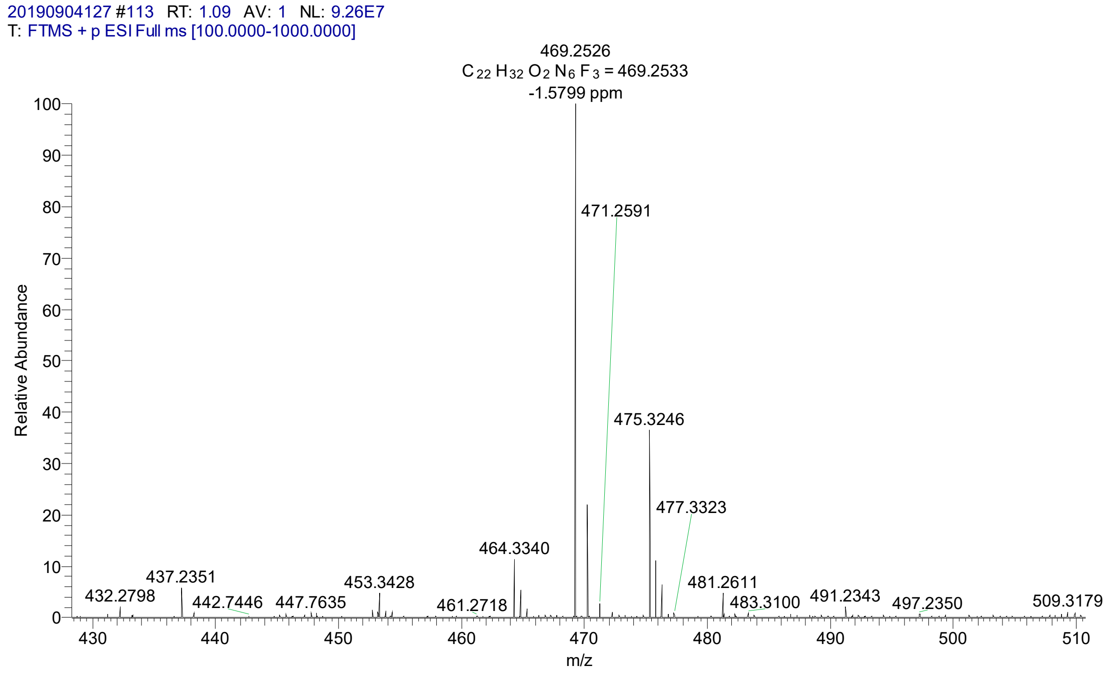

Figure S40. HRMS spectrum of target compound **8d.**

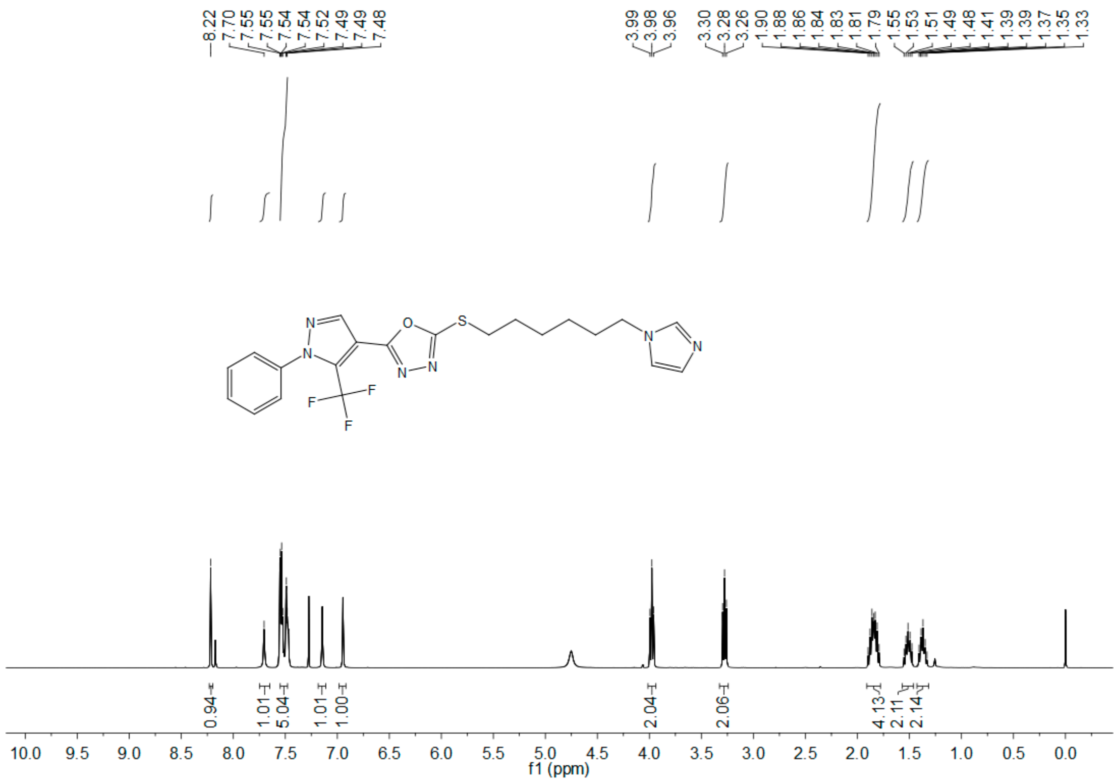

Figure S41. ^{1}H NMR spectrum of target compound **9a.**

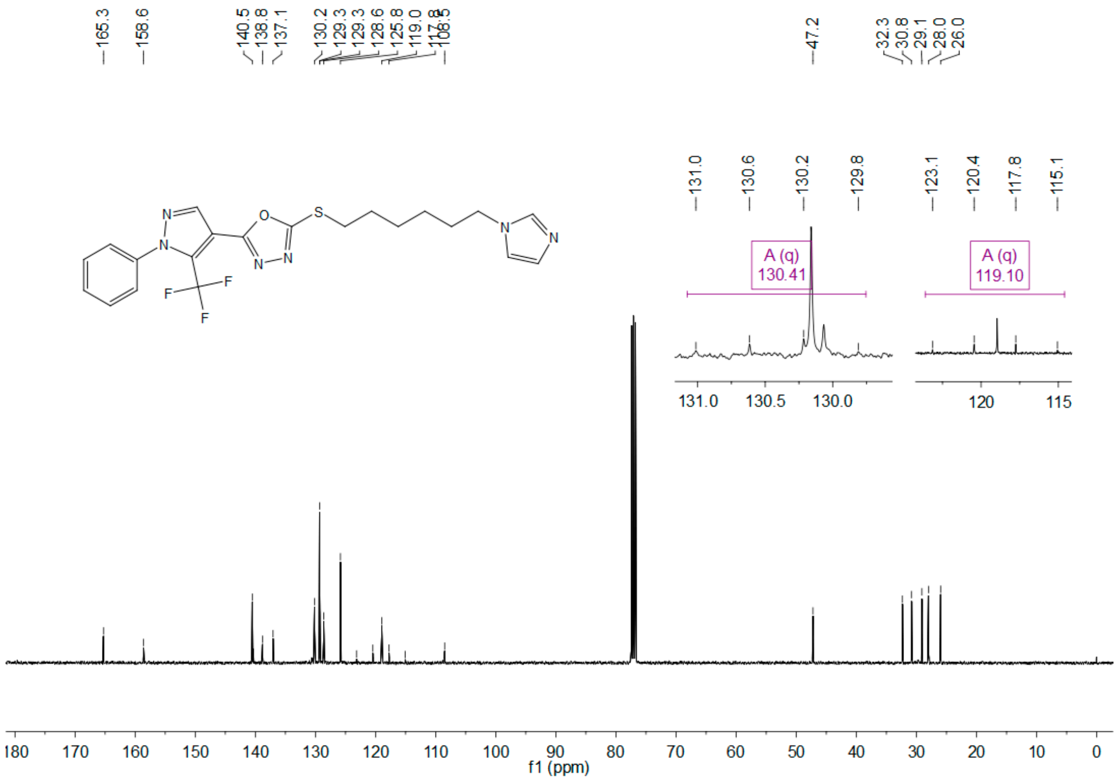

Figure S42. ^{13}C NMR spectrum of target compound **9a.**

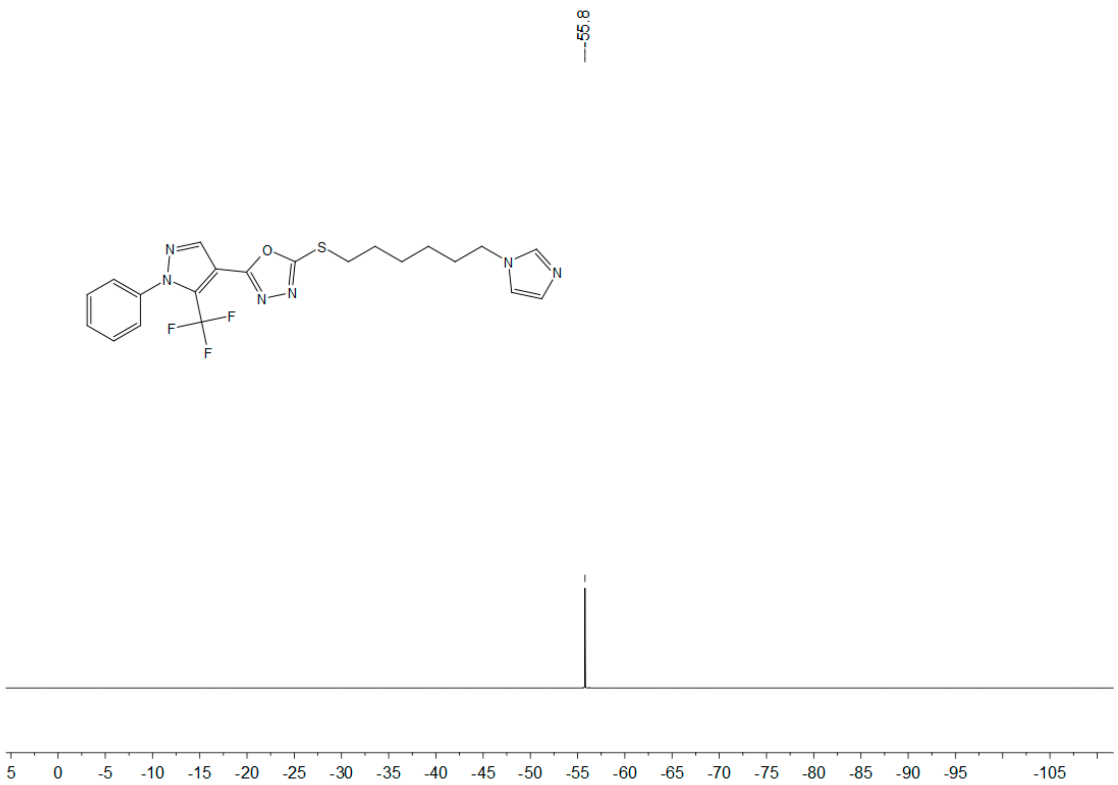

Figure S43. ^{19}F NMR spectrum of target compound **9a.**

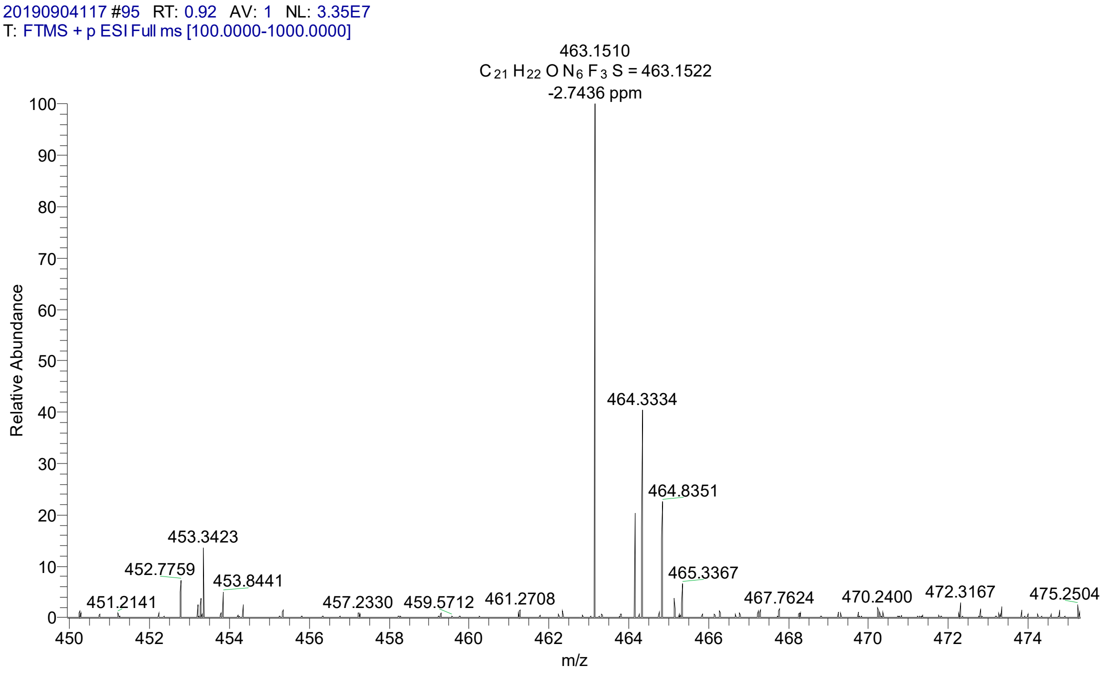

Figure S44. HRMS spectrum of target compound **9a.**

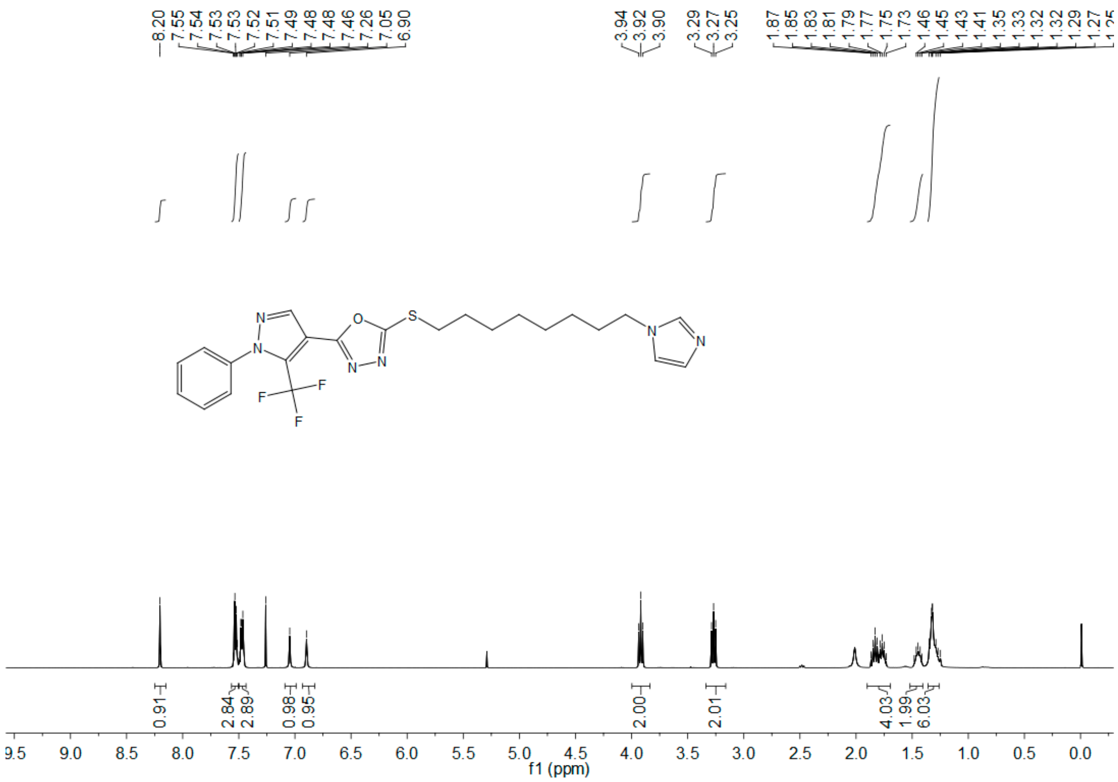

Figure S45. ^{1}H NMR spectrum of target compound **9b.**

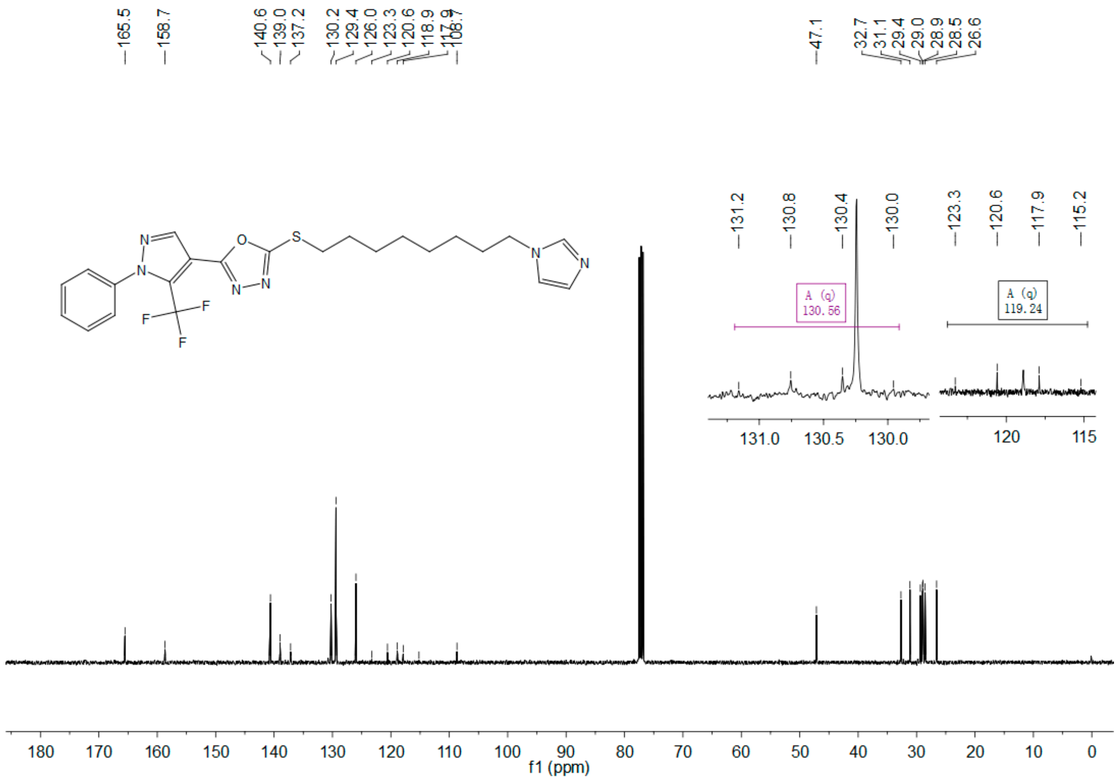

Figure S46. ^{13}C NMR spectrum of target compound **9b.**

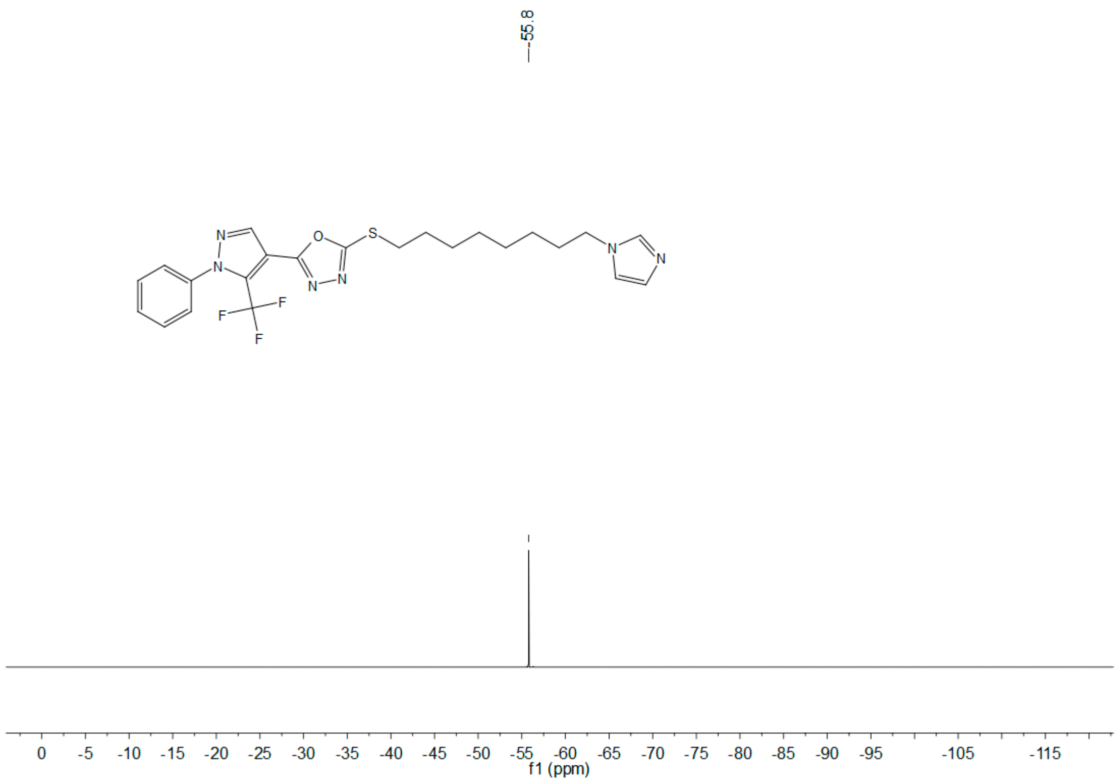

Figure S47. ^{19}F NMR spectrum of target compound **9b.**

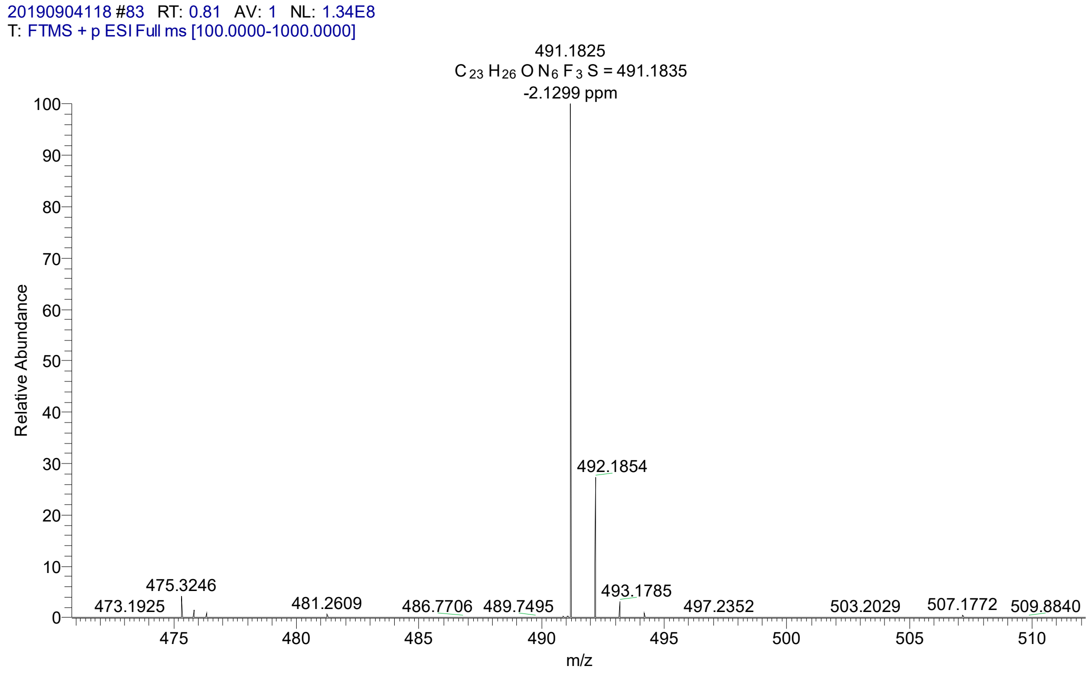

Figure S48. HRMS spectrum of target compound **9b.**

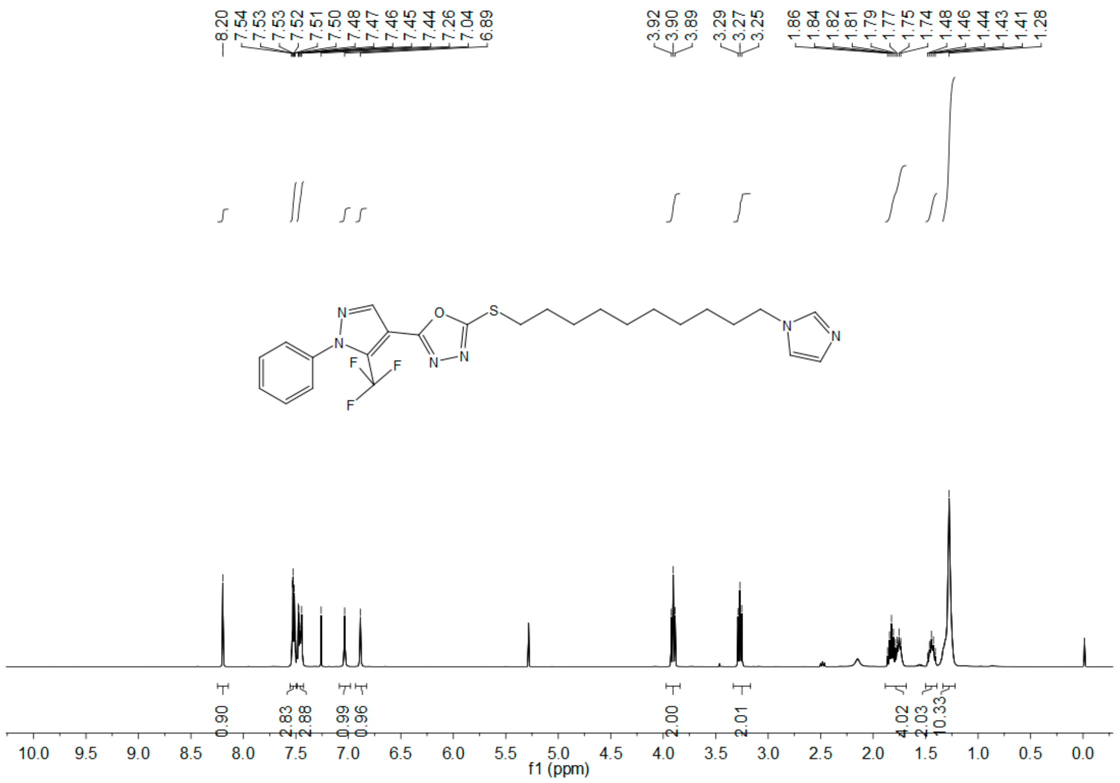

Figure S49. ^{1}H NMR spectrum of target compound **9c.**

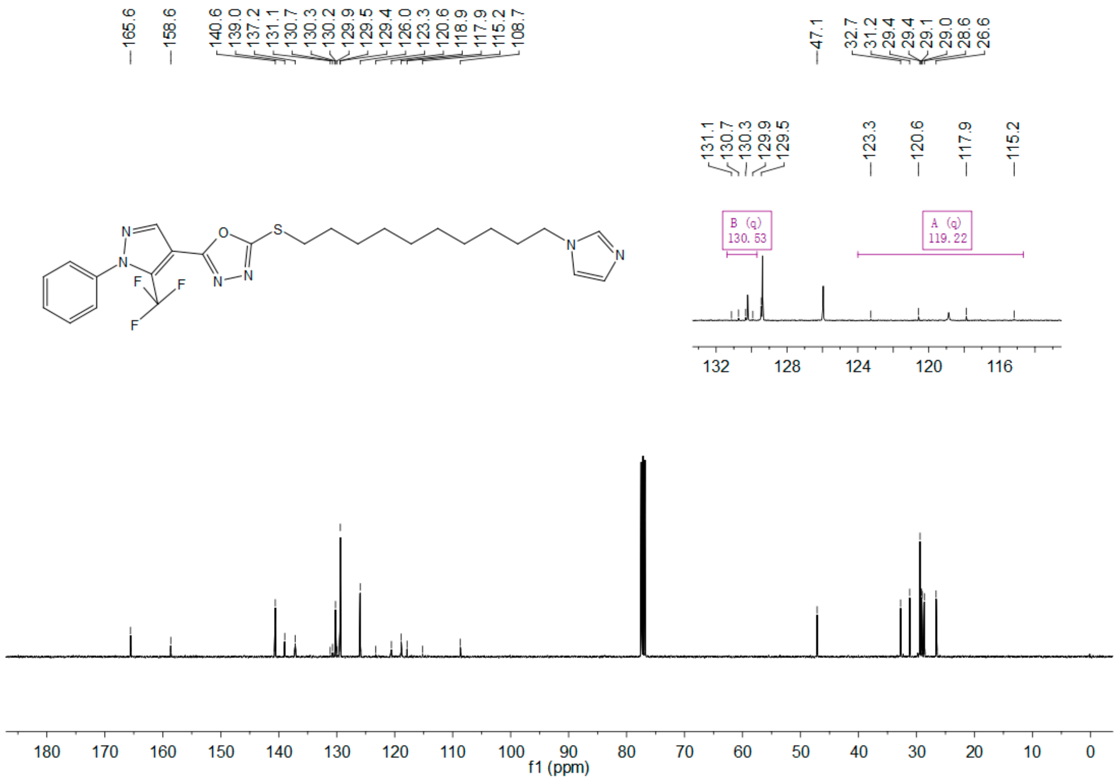

Figure S50. ^{13}C NMR spectrum of target compound **9c.**

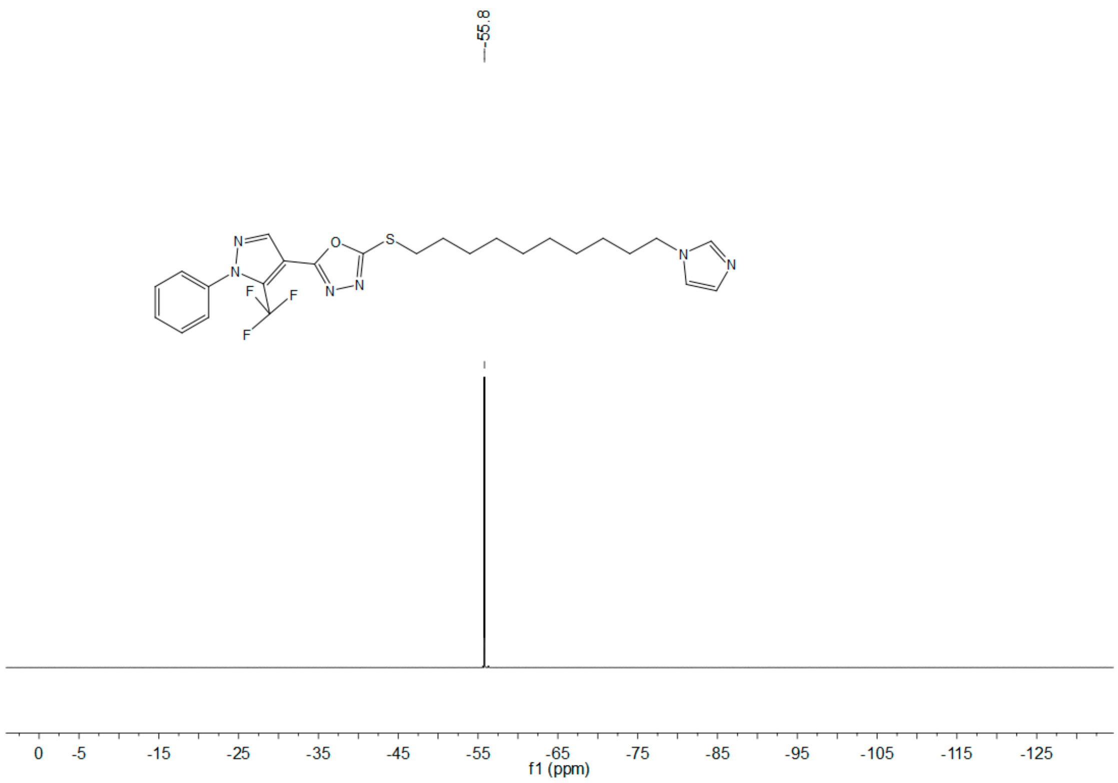

Figure S51. ^{19}F NMR spectrum of target compound **9c.**

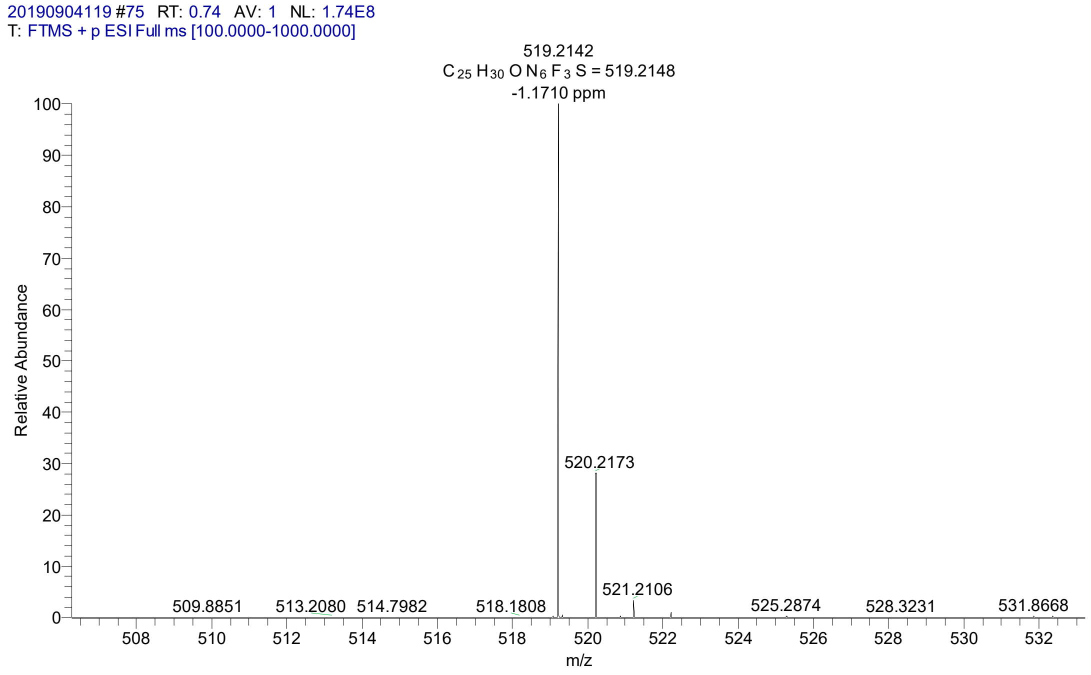

Figure S52. HRMS spectrum of target compound **9c.**

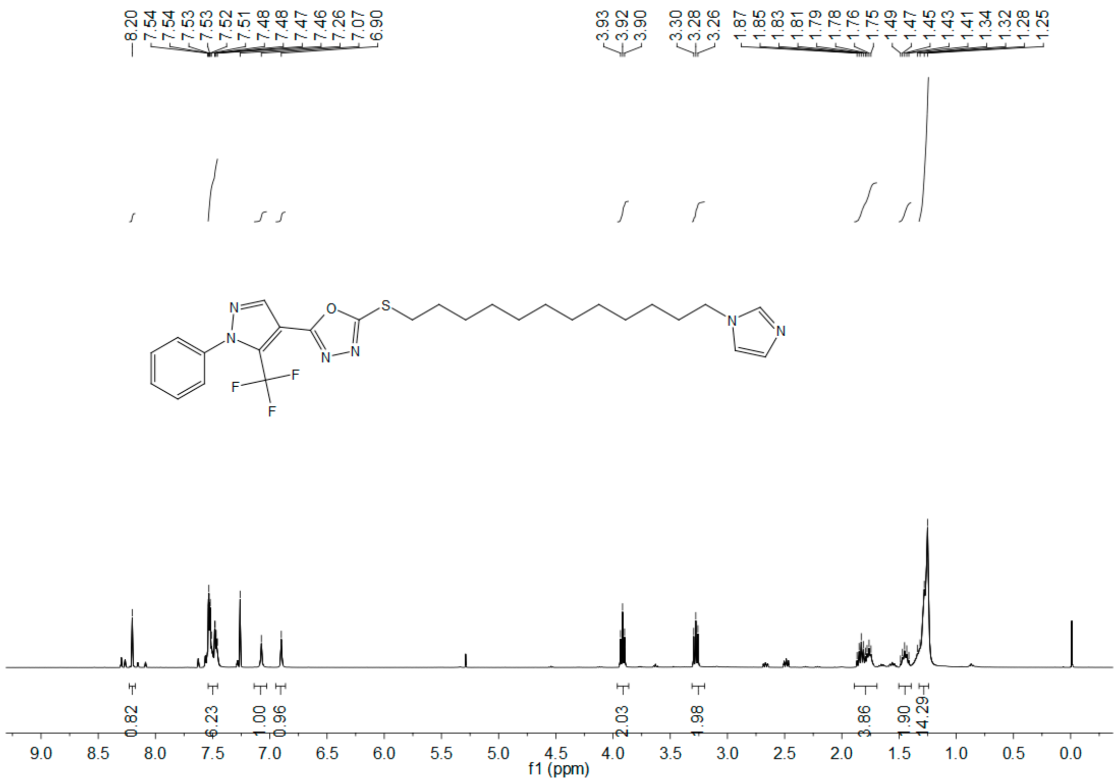

Figure S53. ^{1}H NMR spectrum of target compound **9d.**

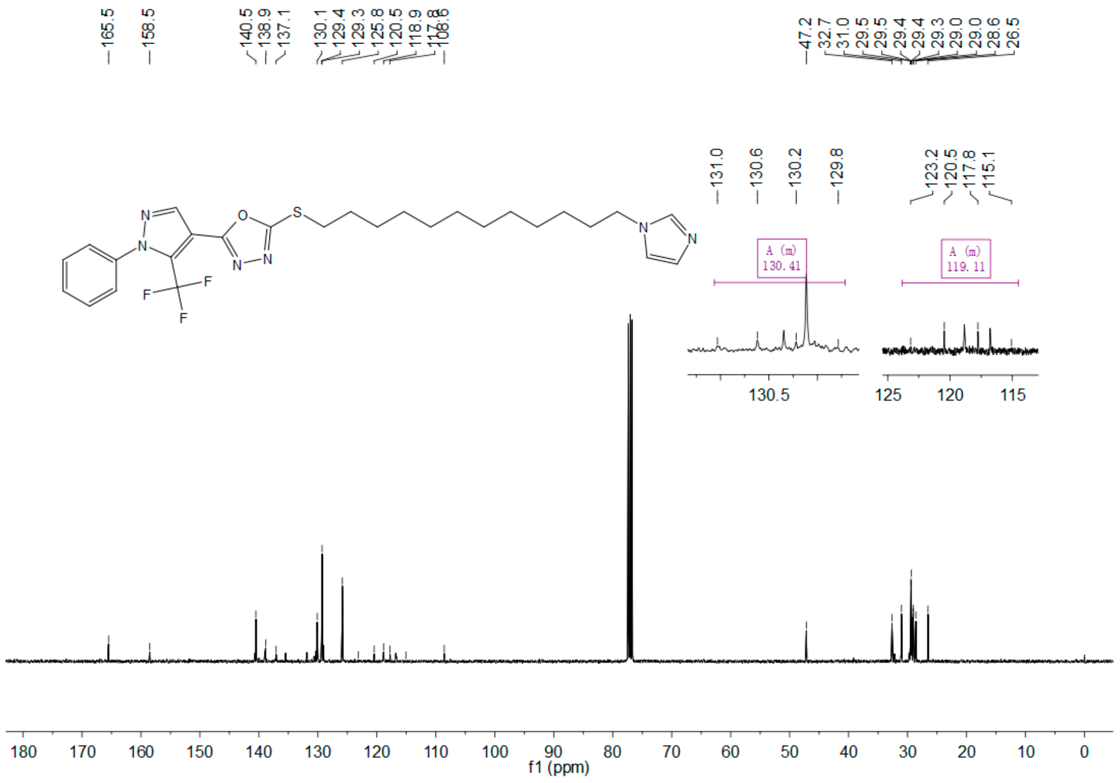

Figure S54. ^{13}C NMR spectrum of target compound **9d.**

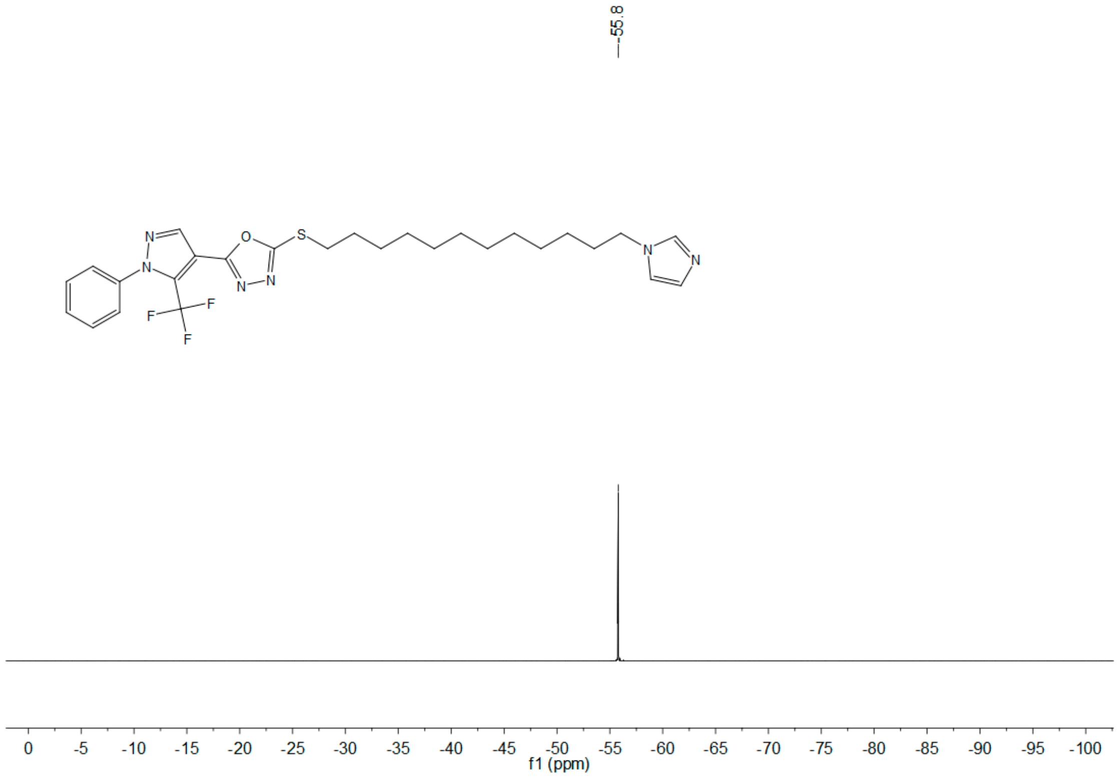

Figure S55. ^{19}F NMR spectrum of target compound **9d.**

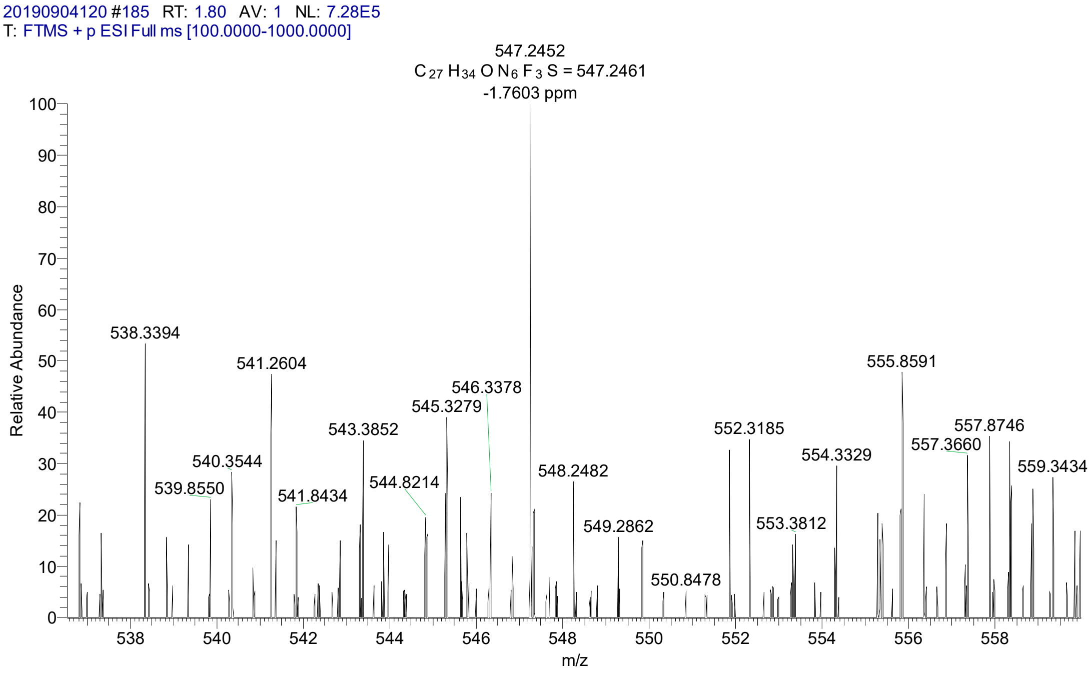

Figure S56. HRMS spectrum of target compound **9d.**

3. *In Vitro* Antibacterial Bioassay of target compounds against *Xoo*, *Xac*, and *Psa*

All the synthesized target compounds were evaluated for their antibacterial activities against *Xoo*, *Xac*, and *Psa* by the turbidimetry test *in vitro*. Dimethyl sulfoxide (DMSO) served as a blank control, and bismerthiazol (BT) and thiodiazole copper (TC) served as positive controls. Approximately 40 μL of solvent NB (1.5 g beef extract, 2.5 g peptone, 0.5 g yeast powder, 5.0 g glucose, and 500 mL distilled water; pH 7.0−7.2) containing *Xoo* (or *Xac*, or *Psa*), incubated on the phase of logarithmic growth, was added to 5 mL of solvent NB containing different concentrations of the test compounds and positive control, such as the preliminary screening experiment use a final concentrations of compounds at 100 and 50 μg/mL, respectively, whereas the EC_{50} tests were use a series concentrations including 50, 25, 12.5, 6.25, and 3.125 μg/mL or 200, 100, 50, 25, and 12.5 μg/mL (depending on the bioactivity of different compounds, the concentrations were chosen in 2 times decline trend to make sure their EC_{50} values which were belong to the ranging of the tested concentrations. The inoculated test tubes were incubated at 28 ± 1 °C and continuously shaken at 180 rpm for 24−48 h until the bacteria of blank control were incubated on the logarithmic growth phase. The growth of the cultures was monitored on a microplate reader by measuring the optical density at 595 nm (OD_{595}), and the inhibition rate *I* was calculated by the following equation:

$$\text{corrected turbidity values} = OD_{\text{bacterial wilt}} - OD_{\text{no bacterial wilt}}$$

$$I\ (\%) = (C - T)/C \times 100\%$$

C is the corrected turbidity value of bacterial growth on untreated NB (blank control), and T is the corrected turbidity value of bacterial growth on treated NB.

In addition, the antibacterial results were analyzed to give their EC_{50} values by using excel 2013 software with the logarithm of concentration [LOG_{10}(concentration)] as x axis and the probit of the relative inhibition rate [5 + NORMSINV(inhibition rate)] as y axis, where more than 3 concentrations with 0 < inhibition rate (%) < 100% were chosen to obtain the toxic regression equation y = a + bx and correlation coefficient R^2. Then, the EC_{50} values were calculated by the followed equation EC_{50} = POWER(10, (5-a)/b), and shown as the average mean ± standard deviation (SD) of three replicate data.

4. *In Vivo* Bioassay of Compound **7c** against Rice Bacterial Leaf Blight

The curative and protection activities of compound **7c** against rice bacterial leaf blight were determined by the method reported in our privious works. The bactericides BT (90% active ingredient) and TC (20% suspending agent) were served as the positive controls.

The curative activity in potted plants of compound **7c** for reducing rice bacterial leaf blight was determined under controlled conditions in a growth chamber. After sowing the rice seeds of variety "Fengyouxiangzhan" approximately 8 weeks, rice leaves were inoculated with *Xoo*, which was incubated at logarithmic growth using sterilized scissors. 24 h after inoculation, **7c** solution at 200 μg/mL was uniformly sprayed onto the rice leaves until dripping down, and distilled water was uniformly sprayed onto the negative control plants. Then, all inoculated rice plants were placed in a plant growth chamber (28 °C and 90% RH). At 14 days after spraying, the disease index of the inoculated rice leaves was measured.

Similarly, the protection activity for reducing rice bacterial leaf blight of compound **7c** was also evaluated under controlled conditions. After sowing the rice seeds of variety “Fengyouxiangzhan” approximately 8 weeks, compound **7c** solution at 200 μg/mL was uniformly sprayed onto the rice leaves until dripping down, whereas distilled water was uniformly sprayed onto the negative control plants. 24 h after spraying, *Xoo* was incubated at logarithmic growth, was inoculated on the rice leaves using sterilized scissors. All inoculated rice plants were placed in a growth chamber (28 °C and 90% RH). At 14 days after inoculation, the disease index (C or T) of the inoculated rice leaves was measured.

Further, the spot length of each leaf and the whole leaf area were measured, and then the percentage of the spot area in the whole leaf area was calculated. Second, these leaves were classed according to the following grading standards:

grade 1, the area of disease spot accounts for less than 5% of the whole leaf area;

grade 3, the area of disease spot accounts for 6−10% of the whole leaf area;

grade 5, the area of disease spot accounts for 11−20% of the whole leaf area;

grade 7, the area of disease spot accounts for 21−50% of the whole leaf area;

grade 9, the area of disease spot accounts for more than 50% of the whole leaf area.

Finally, the disease index (C or T) was calculated using the following formula: Disease index (C or T) = ∑(the number of leaves at each grade × the corresponding grade)/(the total number of leaves × the superlative grade)

The control efficiencies *I* for the curative and protection activities are calculated by the following equation:

$$\text{Control efficiency } I\ (\%) = (C - T)/C \times 100$$

In the equation, C is the disease index of the negative control and T is the disease index of the treatment group.

5. Morphological observation of *Xoo* cells by scanning electron microscopy (SEM)

Brief, compound **7c** were added into 1.5 mL *Xoo* solution which incubated at the logarithmic phase to give at concentration of 0×EC_{50} (equivalent volume of DMSO, CK), 1×EC_{50} (7.40 μg/mL), 2×EC_{50} (14.80 μg/mL), 4×EC_{50} (29.60 μg/mL), 8×EC_{50} (59.2 μg/mL), and 16×EC_{50} (118.2 μg/mL), respectively. After incubation, these samples were washed 3 times with PBS (pH = 7.2). Subsequently, the bacterial cells were fixed for 8 h at 4℃ with 2.5% glutaraldehyde, and then dehydrated with graded ethanol series and pure tert-butanol (2 times with 10 min/time). Following dehydration, freezing dried, coated with gold, and visualized using Nova Nano SEM 450.

6. Determination of purity of active compounds **7c** and **9a**

The solutions of a spot of compound **7c** and **9a** in 1.5 mL methanol were prepared carefully. Then high performance liquid chromatography (HPLC) was performed to detecte the purity of compounds **7c** and **9a** using a under the following conditions: Alltima C18 column (3.9 mm ×250 mm, 5 μm), methanol-water (60:40), 0.5mL/min, 254 nm, 35℃. Finally, the results were reported from the As indicated in **Figure S57** and **Figure S58**, the by the a Agilent 1260 Infinity II (Agilent Technologies Inc. USA).

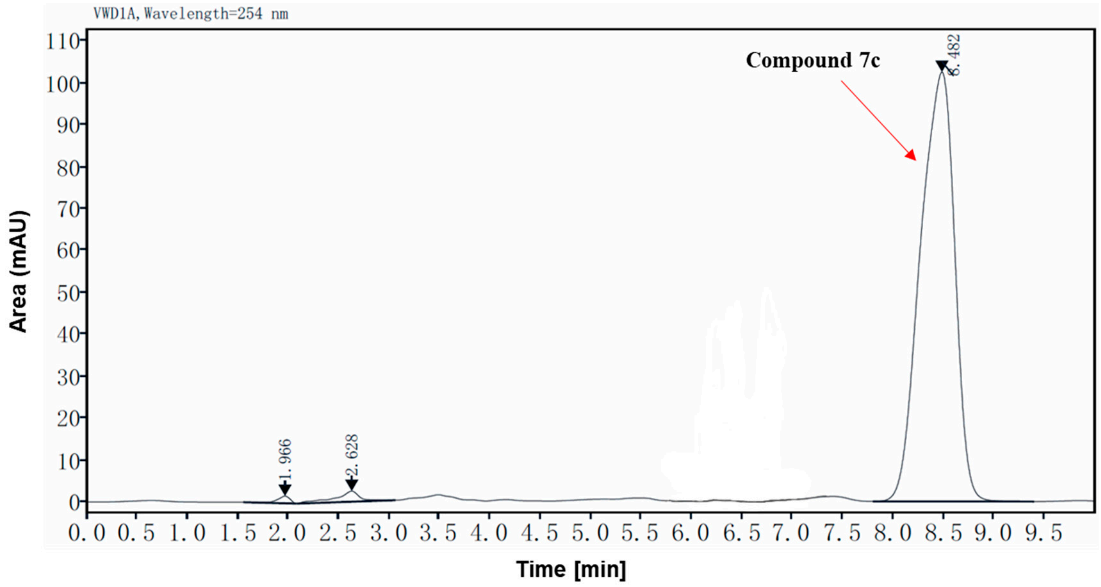

Figure S57. HPLC analysis rsults of compound **7c**

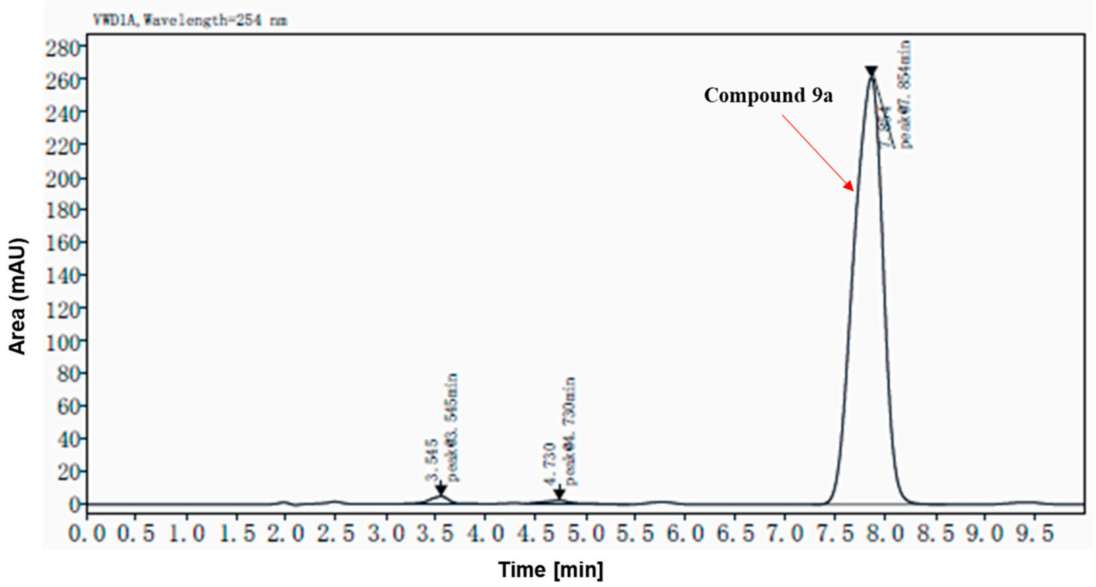

Figure S58. HPLC analysis results of compound **9a**

Table S1. The purity of active compounds **7c** and **9a** from HPLC test.

compd.	Peak Area/mAU	retention time (%)	Purity (%)
**7c**	2418.222	8.482	
	43.726	2.628	97.61%
	15.60	1.966	
	5320.213	7.854	
**9a**	47.082	4.730	97.94%
	64.982	3.545	

7. The ADME properties prediction of compounds **7c** and **9a**

For the ADME properties of compounds 7c and 9a were assessed by using the ADMETlab 2.0 software.

Table S2. The results of ADME properties prediction of compounds **7c** and **9a**

Compd.	**7c**	**9a**
LogP	3.815	4.230
LogS	-4.380	-4.569
LogD	4.039	4.284
AMES Toxicity	--	+
Eye corrosion	--	---
Eye irritation	--	---
Lipinski rule	accepted	accepted
Golden Triangle	accepted	accepted

8. The toxic regression equation and correlation coefficient (R^2) of active compounds against *Xoo*, *Xac* and *Psa*

Table S3. The toxic regression equation and correlation coefficient (R^2) of active compounds against *Xoo*, *Xac* and *Psa*

Comp.	*Xoo*			*Xac*			*Psa*		
	regression eq.	R^2	EC_{50} (μg/mL)	regression eq.	R^2	EC_{50} (μg/mL)	regression eq.	R^2	EC_{50} (μg/mL)
**7a**	y=1.243x+3.896	0.9394	7.73±1.25	y=2.295x+1.282	0.9957	41.69 ± 0.59	y=1.017x+3.363	0.9092	40.71±0.09
**7b**	y=5.902x-0.959	0.9184	10.23±0.20	y=2.057x+1.955	0.9955	30.26 ± 1.85	y=1.145x+3.337	0.9975	28.40±1.53
**7c**	y=3.483x+1.972	0.9906	7.40±1.26	y=1.793x+3.118	0.9475	11.22 ± 0.09	-	-	> 50
**7d**	y=9.032x-3.130	0.9986	7.95±0.12	-	-	> 50	-	-	> 50
**7e**	y=7.007x-1.613	0.8183	8.78±0.31	y=0.657x+3.972	0.9677	36.63±0.71	-	-	> 50
**8b**	y=6.703x-3.756	0.9981	20.25±0.41	y=1.207x+3.748	0.9731	10.91 ± 0.36	-	-	> 50
**8c**	y=6.581x-2.683	0.9990	14.71±0.22	y=1.755x+3.350	0.9807	8.72 ± 1.20	y=1.131x+3.250	0.9955	35.24±1.58
**8d**	y=1.740x+3.388	0.9647	8.44±0.54	-	-	> 50	-	-	> 50
**9a**	y=9.731x-5.904	0.9013	12.40±0.13	y=0.818x+4.398	0.9569	5.44±0.39	y=1.551x+3.280	0.8606	12.85±1.42
**9b**	y=2.462x+2.759	0.9157	8.13±0.51	y=0.877x+4.092	0.9815	10.84±0.32	-	-	> 50
**9c**	y=1.146x+3.355	0.9982	27.26±1.65	-	-	> 50	-	-	> 50
**TC**	y=4.105x-2.740	0.9973	76.81±2.22	y=1.706x+1.885	0.9500	66.98±0.49	y=3.576x-1.704	0.9335	74.98±3.49
**BT**	y=5.069x–2.625	0.8658	31.94±3.59	y=1.893x+1.776	0.9675	50.51±2.08	y=4.318x–3.893	0.9335	114.76±3.93
